# Intensive Lifestyle Intervention in General Practice to Prevent Type 2 Diabetes among 18 to 60-Year-Old South Asians: 1-Year Effects on the Weight Status and Metabolic Profile of Participants in a Randomized Controlled Trial

**DOI:** 10.1371/journal.pone.0068605

**Published:** 2013-07-22

**Authors:** Wanda M. Admiraal, Everlina M. Vlaar, Vera Nierkens, Frits Holleman, Barend J. C. Middelkoop, Karien Stronks, Irene G. M. van Valkengoed

**Affiliations:** 1 Public Health, Academic Medical Center, University of Amsterdam, Amsterdam, The Netherlands; 2 Internal Medicine, Academic Medical Center, Amsterdam, The Netherlands; 3 Public Health, Leiden University Medical Center, Leiden, The Netherlands; 4 Public Health Service, The Hague, The Netherlands; McGill University, Canada

## Abstract

**Aim:**

To study 1-year effectiveness of an intensive, culturally targeted lifestyle intervention in general practice for weight status and metabolic profile of South-Asians at risk of type 2 diabetes.

**Methods:**

536 South-Asians at risk of type 2 diabetes were randomized to an intervention (n = 283) or control (n = 253) group. The intervention, which was targeted culturally to the South-Asian population, consisted of individual lifestyle counselling, a family session, cooking classes, and supervised physical activity programme. All components of the intervention were carried out by professionals as part of their daily clinical practice. The control group received generic lifestyle advice. Change in weight status and metabolic profile were assessed after 1 year.

**Results:**

After 1 year, 201 participants were lost to follow-up. Remaining participants in intervention (n = 177) and control (n = 158) group had similar baseline characteristics. Weight loss in the intervention group was 0.2±3.3 kg, weight gain in the control group was 0.4±3.1 kg (p = 0.08). Changes in other weight-related measurements did not differ significantly between groups. Furthermore, there were no differences between groups in changes of metabolic profile. All results remained similar after repeating analyses in a multiple imputed dataset.

**Discussion:**

An intensive, culturally targeted, lifestyle intervention of 1 year did not improve weight status and metabolic profile of South-Asians at risk of type 2 diabetes. The laborious recruitment, high drop-out, and lack of effectiveness emphasise the difficulty of realising health benefits in practice and suggest that this strategy might not be the optimal approach for this population.

**Trial Registration:**

Nederlands Trial Register NTR1499

## Introduction

The prevalence of obesity and its related diseases has grown to epidemic proportions in the last few decades; at this point, type 2 diabetes mellitus has become one of the most common chronic diseases in industrialised countries [1].

Several studies have shown that the prevalence and incidence of type 2 diabetes and its associated metabolic risk factors vary between ethnic groups; in particular, people of South Asian origin living in industrialised countries are disproportionally affected [2]–[6]. Not only is the prevalence higher, type 2 diabetes also seems to develop at an earlier age among South Asians than among populations of European origin. South Asians with type 2 diabetes have a higher risk of developing disease related complications than do people of European origin [7], [8]. Therefore, the prevention of new cases of type 2 diabetes and its related diseases among South Asians is imperative and could potentially lead to an important health gain in this population.

The opportunity to reduce obesity and to prevent type 2 diabetes among high-risk individuals through intensive lifestyle intervention has been established in several efficacy trials [9]–[12]. However, studies that have attempted to translate and implement the knowledge and expertise from these efficacy trials to clinical practice have achieved more moderate results [13]–[17]. For instance, the Finnish national diabetes prevention program (FIN-D2D), a single arm study that implemented an intensive lifestyle intervention among residents with a high risk of developing type 2 diabetes in primary health care, only showed a weight reduction of approximately 1 kilogram after 1 year [16]. Despite the more moderate effects, the authors of these studies conclude that lifestyle intervention may still be effective for reducing the risk of type 2 diabetes in high-risk groups [13], [16]. Indeed, previous studies have demonstrated that, even small weight loss may be accompanied by multiple beneficial changes in cardiovascular risk factors in high-risk groups [16]. Despite being disproportionally affected by the burden of type 2 diabetes, the effectiveness of intensive lifestyle interventions among high-risk South Asian populations in industrialised countries has not been determined.

Therefore, the aim of this study was to determine the effectiveness after 1 year of an intensive lifestyle intervention, which was culturally targeted to the population, on the weight status and metabolic profile (glucose metabolism, blood pressure and lipid profile) of 18 to 60-year-old South Asians at risk of type 2 diabetes (i.e. those with impaired glucose tolerance, impaired fasting glucose, or relatively high insulin resistance) registered in general practices in The Hague, Netherlands.

## Methods

The protocol for this trial and supporting CONSORT checklist are available as supporting information; see Checklist S1 and Protocol S1.

### Ethics statement

The Institutional Review Board of the Academic Medical Center (Medical Research Ethics Committee (MREC), Academic Medical Center, Amsterdam) approved the study. All participants provided oral and written informed consent.

### Study population

All those studied were participants of the DHIAAN study, a randomized controlled trial (trial number NTR1499) in general practice. The aim was to study the effectiveness of a culturally targeted, intensive, lifestyle intervention to prevent type 2 diabetes and cardiovascular risk factors among Hindustani Surinamese The term ‘Hindustani-Surinamese’ refers to people of South Asian ancestral origin and their offspring who migrated to the Netherlands via Suriname. The Hindustani Surinamese are the descendants of the labourers from North India – Uttar Pradesh, Uttaranchal, and West Bihar – who were indentured between 1873 and 1917. The two large migration waves of Hindustani Surinamese to the Netherlands were caused mainly by the political situation in Suriname. The first wave took place at the time of the independence of Suriname in 1975, and the second wave, at the time of Desi Bouterse's coup in February 1980 [18].

The detailed methods of this study and the process of adapting the lifestyle intervention for the socio-cultural and socio-cognitive determinants of Hindustani Surinamese have been described earlier [19], [20]. In brief, 2307 Hindustani Surinamese (18–60 years old) living in The Hague, Netherlands, were screened via general practices between 18 May 2009 and 11 October 2010. In order to achieve a high response rate, we used a culturally targeted, intensive recruitment strategy that had been evaluated and proven feasible in the pilot of the DHIAAN study [20]. General practitioners sent each potential participant an invitation with a reply card that could be returned if further contact was unwanted. Invitees who had not responded received a written reminder and were contacted by telephone.

We asked all screening participants to fill out a brief questionnaire, undergo a physical exam, and provide a fasting blood sample. The 968 participants who were invited and screened between 18 May 2009 and 18 April 2010 also took an oral glucose tolerance test (75 g).

### Inclusion in the trial

We invited the screening participants to participate in the trial if they had an impaired fasting glucose (5.7–6.9 mmol/l), an impaired glucose tolerance (2-h post-load glucose of 7.8–10.9 mmol/l), a glycated haemoglobin level of 42–46 mmol/mol, and/or a value of 2.39 or more for the homeostasis model assessment of estimated insulin resistance (HOMA-IR) [19] We excluded anyone who was already involved in a lifestyle programme, was pregnant, was known to have type 2 diabetes or a chronic disease that made participation in the intervention impossible, and/or used drugs interfering with plasma glucose levels. Furthermore, we excluded participants with newly diagnosed type 2 diabetes (i.e. a fasting glucose ≥7.0 mmol/l, a 2-h post-load glucose ≥11.0 mmol/l, or a haemoglobin (Hb)-A1c level ≥48 mmol/mol) and referred them to regular clinical care. We used a computer-generated randomisation list (individual one-to-one randomisation) to randomly assign 536 people to either the intervention group or the control group [19]. In brief, we generated a randomization list using simple randomisation (unstratified, no blocking) functions, and placed sealed envelopes representing the order of allocation into a box for the recruiters. Prior to randomisation, potential trial participants were only informed that the study would compare two lifestyle interventions, simply named programme 1 or programme 2, without detailing the specific design of these interventions. Sealed envelopes were opened in order of placement for each person who consented to randomisation and participation in the trial. Participants were then informed of the procedures for the arm of the trial that they had been assigned to. The masking (de facto masking) of the intervention and control group was maintained during the trial.

### Intervention group

We offered all participants in the intervention group a lifestyle intervention. The design of this intervention was in line with the design of the proven efficacious intervention used in the Study on lifestyle intervention and impaired glucose tolerance Maastricht (SLIM), which aimed to evaluate the effect on glucose tolerance in a European Dutch population [21]. We culturally adjusted the intervention to the Hindustani Surinamese population, as cultural adaptations likely promote the effectiveness of interventions among specific ethnic populations [22]. Both surface and deep structure adaptations were used to make the intervention attractive, appropriate, and ultimately more potentially effective in our population [20], [23].

We used motivational interviewing to base the culturally appropriate, intensive, lifestyle intervention on individual lifestyle counselling. The lifestyle counselling in our study consisted of six to eight sessions (determined per individual, according to the goals specified protocol) in the first 6 months, followed by two booster sessions in the next 6 months, and this counselling aimed at both diet and physical activity. The counsellors were trained dieticians who were familiar with the Hindustani Surinamese culture and dietary habits. We offered the participants a family session with the dietician to decrease the social pressure to eat unhealthily and to increase the social support for a healthful lifestyle within the family. If desired, participants could also attend two cooking classes to improve their skills in how to adjust traditional dishes to make them more healthful.

Furthermore, we offered to supervise a 20-week physical activity programme for all participants in the intervention group. This programme, “exercise on prescription”, has been described elsewhere [24], [25]. Trained coaches monitored the participation in the physical activity programme.

### Control group

We invited the participants in the control groups to join two group sessions led by student dieticians (at baseline and after 1 year). The sessions provided generic information about type 2 diabetes and discussed current guidelines for diet and physical activity. These participants received two flyers (at 3 and 9 months) with simple generic lifestyle advice.

### Data collection

One trial visit for the intervention group and one for the control group was planned at baseline and after 1 year [19]. The invitation procedures for these visits were similar to those for the intensive screening procedures. Participants who did not respond to the invitation for the follow up visit were contacted by telephone and received a written reminder. Besides a written confirmation of their appointment, all participants received a reminder text message on the day prior to their appointment.

A trained interviewer interviewed each participant face to face during the visit. The interviewer noted the self-identified ethnicity and determined the educational level. The categories of educational level were: low – secondary education, primary education, or less; middle – low vocational training, lower secondary education, intermediate vocational training, and higher secondary education; and high – higher vocational training or university. Trained research staff, who were blinded to the allocation of participants to the intervention or control groups, used a standardised protocol for physical examinations. They measured weight on a mechanical scale (Seca 761, Hamburg, Germany) to the nearest 500 g. Height, waist circumference, and hip circumference were measured to the nearest 0.01 m. Body fat was measured to the nearest 0.1% by means of bioelectrical impedance analysis (OMRON BF500, Hoofddorp, Netherlands). All anthropometric measurements were obtained twice and the means were used for analysis. Blood pressure was measured in the seated position (OMRON M5-1, Hoofddorp, Netherlands), up to five times. We calculated the mean from the first two measurements with less than 5 mm Hg difference in both systolic blood pressure and diastolic blood pressure [19].

Both at baseline and after 1 year, all participants provided a fasting blood sample and were offered an oral glucose tolerance test. Measurements of fasting plasma glucose (hexokinase, Roche Diagnostics [RD]), 2-h post-load glucose [75 gram] (hexokinase, [RD]), insulin (immunoassay, sandwich principle, [RD]), HbA1c (high-performance liquid chromatography), total cholesterol, high-density lipoprotein cholesterol, low-density lipoprotein cholesterol, and triglycerides (enzymatic colorimetric method for all cholesterol and triglyceride measurements, [RD]) were carried out according to a standardised protocol at the General Practice Laboratory Foundation, Etten-Leur, Netherlands. HOMA-IR was calculated as (fasting plasma glucose * fasting plasma insulin)/22.5 [26].

### Main outcomes

At the time of registration of the trial, the intended primary outcome was the 3-year incidence of type 2 diabetes. However, as the initial response rate was lower than expected, the recruitment period had to be extended to 2 years. Due to a fixed end date of the study (grant restrictions), the follow-up time was reduced to 2 years. As this period was too short to properly investigate differences in incidence of type 2 diabetes between the control and intervention group, we changed the primary outcome to the more proximal outcomes, namely changes in weight and other weight-related measurements (body mass index, waist circumference, and fat mass) after 1 year. Secondary outcomes were changes in glucose metabolism, blood pressure, and lipid profile after 1 year. The changes of the primary outcomes of the trial were approved by the Ethics Committee of the Academic Medical Center [19].

### Statistical analysis

**Figure 1:**
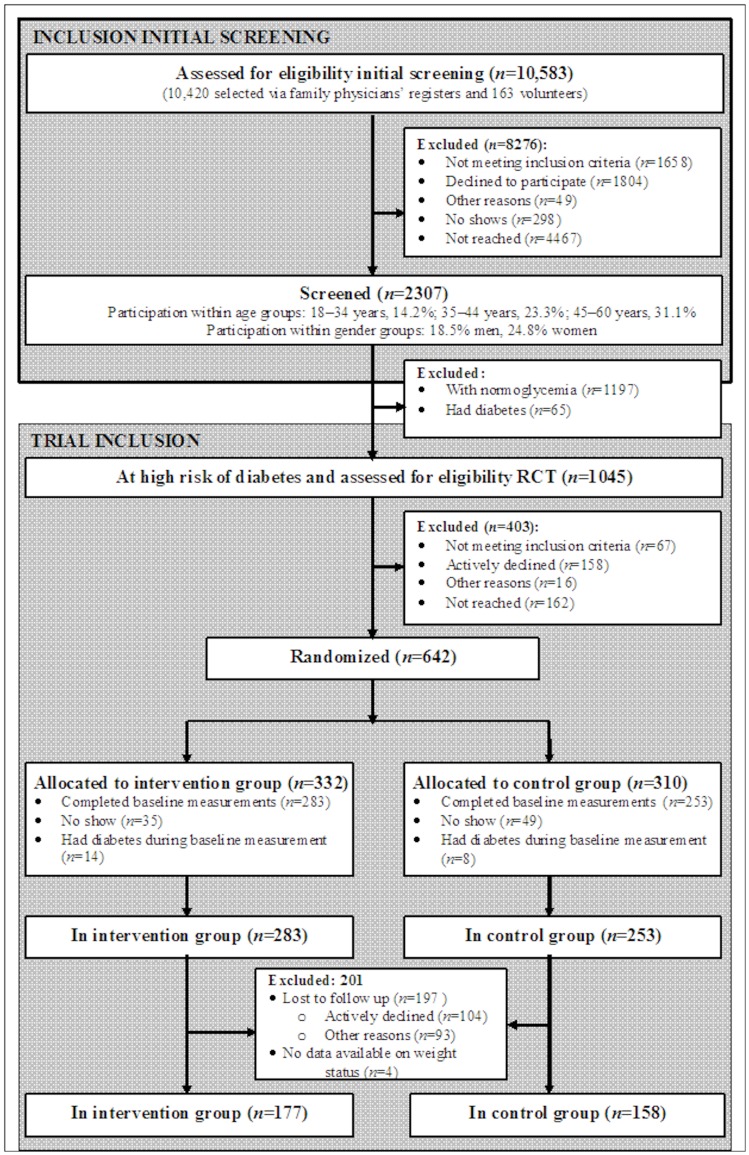
Flow diagram. Normoglycemia was defined as having a fasting glucose <5.7 mmol/l, a 2-h post-load glucose of <7.8, a glycated haemoglobin level of <42 mmol/mol, and a value <2.39 or more for the homeostasis model assessment of estimated insulin resistance. Those with known or newly diagnosed diabetes (i.e. a fasting glucose ≥7.0 mmol/l, a 2-h post-load glucose ≥11.0 mmol/l, and/or a haemoglobin (Hb)-A1c level ≥48 mmol/mol) were excluded from the study.

In the current analysis, we included all those who participated both in the baseline measurement and the follow-up measurement after 1 year, but we excluded anyone without weight data (Figure 1). Of the 536 participants, 197 were lost to follow-up (of which 104 actively declined), and 4 did not have follow-up data available for the weight status. This left 335 participants (177 in the intervention group and 158 in the control group) with data available after a median follow-up time of 380 days (IQR 359–420 days). To evaluate potential selection, we compared baseline characteristics of those with follow-up data available after 1 year with characteristics of those who were lost to follow-up. Apart from being younger (43.3 years versus 45.1 years), those lost to follow-up and those who did have follow-up data after 1 year had similar baseline characteristics (Supplementary Material; Table S1). Furthermore, we found that the odds of being lost to follow-up were similar in the intervention and control group, both in a univariate analysis and in an analysis with adjustment for sex, age, baseline BMI and baseline HbA1c (adjusted OR for the intervention group versus the control group: 0.99 [95%CI 0.70–1.43]).

We also determined whether those who remained in the intervention and control groups had similar baseline characteristics. We described the characteristics with means (with standard deviations) or percentages, and we tested for differences between groups using independent sample *t*-tests and chi-square tests.

Then we described the 1-year changes in weight status, other anthropometric measurements, blood pressure, lipid profile, and glucose metabolism. We used paired *t*-tests to compare these outcomes at baseline and after 1 year in both the intervention group and the control group. In order to determine the effectiveness of the intervention, we compared the changes in these measurements between those assigned to the intervention group and the control group at baseline. We used independent sample *t*-tests to study the differences in continuous measures and chi-square tests to compare the differences in categorical measures. We verified the consistency of the findings across age and sex groups. Additionally, we repeated the main analysis comparing the changes in the intervention versus the changes in the control group with non-parametric tests.

We carried out several additional analyses. First, to determine whether potential dependencies influenced our results between participants belonging to the same general practice, we used a two-level linear regression model in which participants (level 1) were nested within general practices (level 2). To allow for dependencies, we incorporated a random intercept (level 2) into the model. We then determined whether the estimates for the fixed effects in these analyses were similar to the results of the original analyses, and we graphically investigated the random components of the model. Given that there were only 14 people with family members in the study who had follow-up data available, we refrained from carrying out a similar multilevel analysis with the family data.

Moreover, in order to verify the robustness of our results after the loss to follow-up, we repeated the main analyses after using multiple imputation to replace the missing values for the changes after 1 year in weight status, blood pressure, lipid profile, and glucose metabolism [27]. In these analyses, all participants were included, as opposed to only those who had follow-up data available. The imputation involved creating five different datasets in SPSS 19.0 using Fully Conditional Specification procedures (with logistic regression for categorical variables and linear regression for continuous variables). All available data were included in the imputation model. Thereafter, the multiple datasets were analysed as described above, and pooled estimates were computed. We then determined whether the results of the analyses with the multiply imputed data differed from our results of the analyses with complete-case data.

Finally, we conducted several explanatory analyses to further examine potential factors associated with the effects of the intervention after 1 year within the intervention group. First, we described the participation in the different components of the intervention. Second, we compared differences in the change in weight status and the metabolic profile between those attending lifestyle counselling (≥2 sessions attended on top of an intake visit) and those not attending, and between those maintaining participation (≥6 sessions attended on top of an intake visit) and those not maintaining participation. We also distinguished between those who had participated in at least 2 components or in all components of the intervention with those who had not. We based these categories on the goals specified per session in the protocol of the intervention. [19].

We used SPSS 19.0 (SPSS, Chicago, Illinois) for all analyses.

### Power calculation

The original power calculation has already been described [19]. In addition to the power calculation, we performed a post-hoc power calculation study to demonstrate differences in the main outcome (i.e. weight loss) using the numbers of participants who participated both in the baseline measurement and the follow-up measurement after 1 year and had complete data on weight status. As already mentioned, 177 participants in the intervention group and 158 participants in the control group had complete data available both at baseline and after 1 year. Previous studies have found a difference in weight reduction of 1.5–3 kg (SD of 4) after 1 year [12], [28]. Assuming an alpha of 5%, we had a power of 81% to demonstrate a minimum difference in weight reduction of 1.25 kg.

## Results

The mean age of the total population was 45.1 (SD 9.9) years, 50.7% were men, and 11.5% had a low level of education (Table 1). The mean age, sex, and level of education did not differ between the intervention and control groups. The intervention group appeared to have a somewhat higher mean body weight and BMI at baseline than the control group, although these differences were not statistically significant. The control and intervention groups had similar blood pressures, lipid profiles, and glucose metabolisms (Table 1).

**Table 1:** Baseline characteristics of participants with baseline and follow-up measurements.

Characteristic	Total group (n = 335)	Intervention group (n = 177)	Control group (n = 158)
**Age in years**	44.9 (10.1)	44.7 (10.6)	45.0 (9.5)
**Men in n (%)**	170 (50.7)	89 (50.3)	81 (51.3)
**Educational level in n (%)** a			
Low	39 (11.6)	18 (10.4)	20 (12.8)
Middle	226 (67.5)	119 (67.1)	107 (67.9)
High	69 (20.7)	39 (22.0)	30 (19.2)
**Weight status**			
Body weight in kg	75.1 (13.5)	76.3 (14.1)	73.7 (12.7)
Body mass index in kg/m^2^	27.7 (3.9)	28.1 (3.8)	27.2 (3.8)
Overweight: BMI ≥23 kg/m^2^, in n (%)^b^	304 (90.7)	166 (93.8)	138 (87.3)
Obesity: BMI ≥27.5 kg/m^2^, in n (%)^b^	155 (46.3)	86 (48.6)	69 (43.7)
Waist circumference in cm	93 (10)	94 (11)	92 (10)
Hip circumference in cm	98 (9)	99 (9)	98 (8)
Fat mass in %	36.2 (9.3)	36.6 (9.2)	36.0 (9.3)
**Glucose metabolism**			
HbA1c in mmol/mol	39 (4)	39 (4)	38 (4)
HbA1c in %	5.7 (0.4)	5.7 (0.4)	5.6 (0.4)
Fasting plasma glucose in mmol/l	5.3 (0.5)	5.3 (0.5)	5.3 (0.5)
2-h post-load glucose in mmol/l	6.1 (1.7)	6.3 (1.6)	5.9 (1.8)
Fasting plasma insulin in pmol/l	14 (9)	15 (9)	14 (8)
**Blood pressure**			
Systolic pressure in mm Hg	130 (18)	130 (16)	129 (19)
Diastolic pressure in mm Hg	83 (11)	84 (10)	83 (11)
**Lipid profile**			
Total cholesterol in mmol/l	5.01 (0.96)	5.05 (0.89)	5.00 (1.04)
HDL cholesterol in mmol/l	1.27 (0.31)	1.26 (0.30)	1.29 (0.34)
LDL cholesterol in mmol/l	3.19 (0.89)	3.26 (0.83)	3.12 (0.95)
Triglycerides in mmol/l	1.34 (0.98)	1.26 (0.65)	1.25 (0.63)

BMI  =  Body mass index, HbA1c  =  haemoglobin A1c, HDL  =  high-density lipoprotein, LDL  =  low-density lipoprotein.

Data in parentheses are standard deviations following means or percentages following n.

a Educational level is the highest education achieved and is classified as: low  =  secondary education, primary education, or less; middle  =  low vocational training, lower secondary education, intermediate vocational training, and higher secondary education; and high  =  higher vocational training or university.

b Cut-off values for population of South Asian origin.

The intervention group had a mean weight loss of 0.2 kg after 1 year (p = 0.32), whereas those in the control group gained 0.4 kg (p = 0.14; Table 2). There were small changes in waist circumference, fat mass, and blood pressure in both groups. Although the mean level of the fasting blood glucose was lower after 1 year in both groups, there was no change in mean HbA1c level in either group.

**Table 2:** Changes after 1 year between the control group and the intervention group.

	Intervention group (n = 177)			Control group (n = 158)			Between group +/−	
	Baseline Mean (SD)	Follow-up Mean (SD)	Change	Baseline Mean (SD)	Follow-up Mean (SD)	Change	Difference (95%CI)^b^	P value^b^
**Weight status**								
Body weight in kg	76.3 (13.5)	76.1 (13.7)	−0.2 (3.3)	73.7 (12.7)	74.1 (12.9)	+0.4 (3.1)	0.6 (−0.1, 1.3)	0.08
BMI in kg/m^2^	28.1 (3.9)	28.0 (4.0)	−0.1 (1.2)	27.2 (3.8)	27.4 (3.8)	+0.1 (1.1)	0.2 (−0.04, 0.5)	0.09
Waist circumference in cm	94 (11)	95 (10)	+1 (5) c	92 (10)	94 (13)	+2 (10) c	1 (−1, 2)	0.50
Hip circumference in cm	99 (9)	99 (8)	0	98 (8)	98 (8)	0	0 (−0.2, 2)	0.13
Fat mass in %	36.6 (9.2)	35.7 (9.1)	−0.9 (3.4) c	36.0 (9.3)	35.1 (9.3)	−0.9 (2.7) c	0 (−0.6, 0.7)	0.84
Weight change in %	N/A	−0.2 (4.2)	N/A	N/A	0.6 (4.0)	N/A	0.8 (−0.1, 1.6)	0.10
**Change in weight** a								
No change in %	N/A	32.2	N/A	N/A	38.9	N/A	6.7 (−3.6, 17.0)	0.12
Any weight loss in %	N/A	37.3	N/A	N/A	19.1	N/A	18.2 (8.6, 27.8)	<0.01
>5% weight loss in %	N/A	10.7	N/A	N/A	8.2	N/A	2.5 (−3.8, 8.8)	0.28
Weight gain in %	N/A	30.5	N/A	N/A	41.8	N/A	11.3 (0.9, 21.5)	0.02
**Glucose metabolism**								
HbA1c in mmol/mol	39 (4)	39 (4)	0 (3)	39 (4)	39 (4)	0 (3)	0 (−1, 1)	0.99
HbA1c in %	5.7 (0.4)	5.7 (0.4)	0 (0.4)	5.7 (0.4)	5.7 (0.4)	0 (0.3)	0 (−0.1, 0.1)	0.99
Fasting plasma glucose in mmol/l	5.3 (0.5)	4.9 (0.9)	−0.5 (0.9) c	5.3 (0.5)	4.8 (0.8)	−0.5 (0.8) c	0.0 (−0.14, 0.22)	0.66
2-h post-load glucose in mmol/l	6.3 (1.6)	6.3 (1.9)	0.0 (1.9)	5.9 (1.8)	6.0 (1.8)	+0.1 (1.9)	0.1 (−0.35, 0.55)	0.44
Fasting plasma insulin in pmol/l	15 (9)	13 (8)	−2 (7) c	14 (8)	13 (12)	−1 (11)	1 (−1, 4)	0.12
HOMA-IR^d^	3.6 (2.4)	2.7 (1.7)	−0.8 (2.0)^c^	3.3 (2.0)	2.8 (2.0)	−0.5 (2.0)^c^	0.3 (−0.2, 0.7)	0.22
**Blood pressure**								
Systolic pressure in mm Hg	130 (16)	132 (19)	+2 c	129 (19)	129 (0)	0	2 (−1, 4)	0.18
Diastolic pressure in mm Hg	84 (10)	82 (10)	−2 c	83 (11)	81 (10)	−2 c	0 (−1, 2)	0.51
Lipids								
Total cholesterol in mmol/l	5.05 (0.89)	5.17 (0.97)	+0.12 (0.78) c	5.00 (1.04)	5.07 (1.07)	+0.08 (0.96) c	0.04 (−0.15, 0.23)	0.67
HDL cholesterol in mmol/l	1.26 (0.30)	1.36 (0.33)	+0.10 (0.16) c	1.29 (0.34)	1.36 (0.35)	+0.07 (0.22) c	0.03 (−0.02, 0.70)	0.24
LDL cholesterol in mmol/l	3.26 (0.83)	3.28 (0.87)	+0.02 (0.69)	3.12 (0.95)	3.15 (1.02)	+0.03 (0.87)	0.01 (−0.17, 0.18)	0.97
Triglycerides in mmol/l	1.26 (0.65)	1.26 (0.62)	0.00 (0.53)	1.25 (0.63)	1.21 (0.91)	−0.04 (0.56)	0.04 (−0.09, 0.16)	0.55

BMI  =  Body mass index, HbA1c  =  haemoglobin A1c, HDL  =  high-density lipoprotein, LDL  =  low-density lipoprotein, N/A  =  not applicable, 95%CI  = 95% confidence interval, HOMA-IR  =  homeostasis model assessment- insulin resistance.

Data in parentheses are standard deviations following means.

a Any weight loss  =  weight loss >1 kg; weight gain  =  weight gain >1 kg.

b The differences with corresponding 95%CI's in changes over time between control group and intervention group were determined with independent sample *t*-tests for continuous measures and chi-square tests for categorical measures.

c P<0.05 for differences between baseline and follow-up measurements of metabolic characteristics determined with paired *t*-tests.

d HOMA-IR was calculated as (fasting plasma glucose * fasting plasma insulin)/22.5 [26].

In accordance with these results, we found no differences between the intervention group and the control group in changes in weight-related measurements, glucose metabolism, blood pressure, and lipid profile after 1 year (Table 2). For example, the difference between the two groups in change of weight after 1 year was 0.6 kg (95%CI −0.1, 1.3), and the difference in HbA1c change was 0 (95%CI −1, 1) mmol/mol (0 [−0.1, 1.1] %). These findings were consistent across sex and age groups. The analyses with non-parametric tests showed comparable results (data not shown). Moreover, there was no evidence that potential dependencies between participants belonging to the same general practice influenced our results. There was no significant variation at the level of general practices.

Repeating the analyses in the multiply imputed dataset did not alter our results; both the absolute changes within the intervention and control groups, and the differences between the groups with their associated p values remained similar (data not shown).

Finally, the explanatory analyses in the intervention group showed that 81% had participated in the individual lifestyle counselling sessions, with a median number of 5 sessions (IQR 1−9) per person (on top of an initial intake visit). Moreover, 26% participated in a supplemental family session, 26% in the cooking classes and 22% in the supervised exercise sessions. We did not find differences in the change of any metabolic risk factors between those who attended or maintained lifestyle counselling and those who did not (Table 3 and Table 4). In addition, we did not find differences between those who participated in at least 2 components or in all components of the intervention and those who did not (data not shown).

**Table 3:** By treatment analysis: difference in obesity and metabolic characteristics between those attending the lifestyle counselling (≥2 sessions attended) and those not attending within the intervention group.

	Intervention group nonattenders (n = 46)			Intervention group attenders a (n = 131)			Between group +−	
	Baseline Mean (SD)	Follow-up Mean (SD)	Change	Baseline Mean (SD)	Follow-up Mean (SD)	Change	Difference (95%CI)	P value b
**Weight status**								
Body weight kg	77.2 (13.7)	77.3 (14.4)	+0.1 (3.0)	76.0 (14.2)	75.7 (13.4)	−0.3 (3.4)	0.4 (−0.6, 1.6)	0.41
BMI in kg/m^2^	28.4 (4.1)	28.4 (4.2)	0.0 (1.1)	28.0 (4.1)	27.9 (3.9)	−0.1 (1.2)	0.1 (−0.3, 0.5)	0.54
Waist circumference in cm	94 (12)	96 (11)	+2 (5) c	94 (11)	95 (10)	+1	1 (−0.2, 3)	0.09
Hip in circumference	100 (9)	100 (8)	0 (3)	99 (9)	99 (8)	0 (4)	0 (−1, 2)	0.35
Fat mass in %	35.4 (10.1)	34.3 (9.9)	−1.1 (3.5) c	37.0 (8.9)	36.1 (8.9)	−0.9 (3.3) c	0.2 (−0.9, 1.4)	0.63
Weight change in %	N/A	0.1 (3.8)	N/A	N/A	−0.3 (4.3)	N/A	0.4 (−1.1, 1.7)	0.66
**Glucose metabolism**								
HbA1c in mmol/mol	39 (4)	40 (4)	+1 (4)	39 (4)	39 (4)	0 (3.0)	1 (−0.3, 2)	0.15
HbA1c in %	5.7 (0.4)	5.8 (0.4)	+0.1 (0.4)	5.7 (0.4)	5.7 (0.4)	0 (0.3)	0.1 (−0.1, 0.2)	0.15
Fasting plasma glucose in mmol/l	5.3 (0.6)	5.0 (1.0)	−0.3 (1.0)	5.3 (0.5)	4.8 (0.8)	−0.5 (0.8) c	0.2 (−0.01, 0.6)	0.07
2-h post-load glucose in mmol/l	6.4 (1.7)	6.6 (2.3)	+0.2 (1.9)	6.2 (1.6)	6.2 (1.8)	−0.0 (2.0)	0.2 (−0.5, 1.0)	0.51
Fasting plasma insulin in pmol/l	17 (11)	14 (8)	−3 (8) c	14 (9)	13 (8)	−1 (7) c	2 (−1, 4)	0.35
**Blood pressure**								
Systolic pressure in mm Hg	131 (20)	133 (31)	+2 (14)	129 (16)	131 (18)	+2 (11)	0 (−4, 4)	0.88
Diastolic pressure in mm Hg	83 (13)	82 (12)	−1 (8)	84 (9)	81 (10)	−3 (8) c	2 (−1, 4)	0.32
**Lipid profile**								
Total cholesterol in mmol/l	5.11 (0.88)	5.00 (0.84)	−0.11 (0.81)	5.02 (0.89)	5.22 (1.00)	+0.20 (0.76) c	0.31 (0.06, 0.61)	0.02
HDL cholesterol in mmol/l	1.20 (0.26)	1.29 (0.31)	+0.9 (0.16) c	1.28 (0.31)	1.38 (0.33)	+0.10 (0.16) c	0.01 (−0.05, 0.07)	0.66
LDL cholesterol in mmol/l	3.34 (0.83)	3.12 (0.70)	−0.21 (0.75)	3.23 (0.84)	3.34 (0.91)	+0.11 (0.65)	0.32 (0.08, 0.56)	0.02
Triglycerides in mmol/l	1.37 (0.69)	1.25 (0.64)	−0.01 (0.49)	1.23 (0.63)	1.23 (0.63)	+0.00 (0.54)	0.01 (−0.16, 0.21)	0.83

BMI  =  Body mass index, HbA1c  =  haemoglobin A1c, HDL  =  high-density lipoprotein, LDL  =  low-density lipoprotein, N/A  =  not applicable, SD  =  standard deviation, 95%CI  = 95% confidence interval.

a Attendance is defined as having had two or more sessions of lifestyle counselling on top of an initial intake visit.

b The differences with corresponding 95%CI's in changes of metabolic characteristics over time between those attending and not attending the lifestyle counselling were determined with independent sample *t*-tests for continuous measures and chi-square tests for categorical measures.

c P<0.05 for differences between baseline and follow-up measurements of metabolic characteristics determined with paired *t*-tests.

**Table 4:** By treatment analysis: difference in obesity and metabolic characteristics between those maintaining participation (≥6 sessions attended) in the lifestyle counselling versus those not maintaining participation within the intervention group.

	Intervention group nonattenders (n = 91)			Intervention group attenders a (n = 86)			Between group +−	
	Baseline Mean (SD)	Follow-up Mean (SD)	Change	Baseline Mean (SD)	Follow-up Mean (SD)	Change	Difference (95%CI)	P value b
**Weight status**								
Body weight kg	76.2 (14.0)	76.3 (14.5)	+0.1 (3.3)	76.4 (14.2)	75.8 (12.8)	−0.6 (3.2)	0.7 (−0.3, 1.7)	0.16
BMI in kg/m^2^	28.1 (4.0)	28.1 (4.0)	0 (1.2)	28.0 (4.2)	27.8 (3.9)	−0.2 (1.1)	0.2 (−0.1, 0.6)	0.21
Waist circumfe rence in cm	95 (12)	96 (11)	+1 (5) c	93 (10)	94 (10)	+1 (5)	0 (−0.5, 3)	0.15
Hip in circumference	99 (9)	100 (9)	+1 (4)	99 (9)	99 (8)	0 (5)	1 (−0.6, 2)	0.29
Fat mass in %	36.8 (9.4)	35.9 (9.3)	−0.8 (3.5) c	36.4 (9.1)	35.4 (9.0)	−1.0 (3.2) c	0.2 (−0.8, 1.2)	0.71
Weight change in %	N/A	0.1 (4.4)	N/A	N/A	−0.5 (3.9)	N/A	0.6 (−0.6, 1.8)	0.33
**Glucose metabolism**								
HbA1c in mmol/mol	39 (5)	40 (4)	1 (3) c	39 (5)	39 (3)	0 (3)	1 (0.3, 2)	0.02
HbA1c in %	5.7 (0.5)	5.8 (0.4)	+0.1 (0.3)	5.7 (0.5)	5.7 (0.3)	0 (0.3)	0.1 (0, 0.2)	0.02
Fasting plasma glucose in mmol/l	5.3 (0.6)	5.0 (0.9)	−0.3 (0.9) c	5.3 (0.5)	4.7 (0.8)	−0.6 (0.8)	0.3 (−0.04,0.5)	0.10
2-h post-load glu cose in mmol/l	6.4 (1.7)	6.5 (2.0)	0.1 (2.0)	6.1 (1.6)	6.0 (1.8)	−0.1 (1.9)	0.2 (−0.5, 0.8)	0.66
Fasting plasma insulin in pmol/l	15 (10)	13 (8)	−2 (7) c	15 (9)	12 (8)	−2 (7) c	0 (−1, 3)	0.46
**Blood pressure**								
Systolic pressure in mm Hg	129 (18)	131 (19)	+2 (12)	131 (16)	133(19)	+2 (12)	0 (−3, 4)	0.92
Diastolic pressure in mm Hg	83 (11)	81 (11)	−2 (7)	84 (9)	81 (10)	−3 (8) c	1 (−1, 4)	0.26
**Lipid profile**								
Total cholesterol in mmol/l	5.02 (0.89)	5.12 (0.94)	+0.10 (0.78)	5.07 (0.89)	5.22 (0.99)	+0.15 (0.77)	0.05 (−0.18, 0.30)	0.63
HDL cholesterol in mmol/l	1.22 (0.26)	1.30 (0.29)	+0.08 (0.15) c	1.31 (0.32)	1.42 (0.35)	+0.11 (0.16) c	0.03 (−0.02, 0.08)	0.26
LDL cholesterol in mmol/l	3.24 (0.80)	3.26 (0.75)	+0.02 (0.71)	3.28 (0.88)	3.31 (0.98)	+0.04 (0.67)	0.02 (−0.19, 0.23)	0.88
Triglycerides in mmol/l	1.37 (0.75)	1.35 (0.68)	−0.03 (0.58) c	1.14 (0.50)	1.17 (0.54)	+0.03 (0.46)	0.06 (−0.11, 0.21)	0.51

BMI  =  Body mass index, HbA1c  =  haemoglobin A1c, HDL  =  high-density lipoprotein, LDL  =  low-density lipoprotein, 95%CI  = 95% confidence interval.

a Maintenance is defined as having attended 6 sessions or more for lifestyle counselling (on top of an initial intake visit).

b The differences with corresponding 95%CI's in changes of metabolic characteristics over time between those maintaining and not maintaining participation in the lifestyle counselling, determined with independent sample *t*-tests for continuous measures and chi-square tests for categorical measures.

c P<0.05 for differences between baseline and follow-up measurements of metabolic characteristics determined with paired *t*-test.

## Discussion

### Main findings

We investigated the effectiveness of an intensive, culturally targeted lifestyle intervention in general practice among 18 to 60-year-old Hindustani Surinamese, a population of South Asian origin with a high risk of type 2 diabetes, living in the Netherlands. We found that the lifestyle intervention did not effectively change the weight status, glucose metabolism, blood pressure, or lipid profile in the target population. After 1 year of follow-up, the metabolic risk factors of the intervention and control groups were similar. The attendance and maintenance of the intervention did not explain the lack of change in weight and metabolic profile in the intervention group.

### Limitations of the study

This study has both strengths and limitations that we wish to mention before discussing our main findings. One strength of the study is that it is unique in terms of population (South Asians in industrialised countries), age (young population), and the culturally targeted design of the intervention [20]. Furthermore, no studies have reported investigating the potential effectiveness of an intensive lifestyle intervention in practice in this population, while South Asians in industrialised countries are at high risk of type 2 diabetes and are therefore an important target group.

In spite of the intensive invitation procedures, there was a high loss to follow-up (197 of the 536 participants). This drop-out emphasizes the difficulty of realising health gain by means of a lifestyle intervention in general practice. At the same time, we consider this a limitation of our study. Although those lost to follow-up and those with follow-up data available after 1 year had similar baseline characteristics and loss to follow-up was not associated with group allocation, we cannot rule out selection bias in our study. We attempted to account for the effects of the loss to follow-up by repeating our analyses in a multiply imputed dataset. These analyses did not change our interpretation of the results.

Another limitation was that we measured weight to the nearest 500 g, which influenced the precision of the reported weight and weight change after 1 year. However, this applies equally to the measurements taken in the intervention group and the control group, so that it is unlikely that this limitation has majorly influenced the reported difference in change of weight between the two groups.

### Discussion of the main findings

We found that a culturally targeted intensive lifestyle intervention in general practice did not effectively change the metabolic risk factors among our Hindustani Surinamese study population after 1 year of follow-up. Our results are in line with results from studies that have investigated the effectiveness in clinical practice of lifestyle interventions for the prevention of type 2 diabetes among other ethnic populations [13]–[16]. Although the effects in clinical practice have been smaller than in the large efficacy trials, some clinical studies have reported changes in metabolic risk factors. For example, Vermunt et al., who studied the overall effect of a lifestyle intervention on type 2 diabetes risk reduction in Dutch primary care, found that the changes over time between those receiving the lifestyle intervention and those receiving conventional care were small, and most of them were not significant [13]. In addition, Saaristo et al., who implemented an intensive lifestyle intervention in primary health care among residents with a high risk of developing type 2 diabetes, showed a weight reduction of approximately 1 kilogram after 1 year [16].

The results of our study pinpoint that, despite the potential efficacy among South Asians [17], realising a health benefit by means of an intensive lifestyle intervention among a high risk South Asian population in general practice is extremely difficult. This was somewhat unexpected. As South Asians are known to have a high a-priori risk of developing type 2 diabetes and have a familial disposition toward type 2 diabetes [3], we expected a high level of awareness and motivation to make behavioural changes, especially during a lifestyle intervention trial. Indeed, participants in focus group interviews carried out before our study (unpublished) pointed out the necessity of such an intervention for this population. In addition, the intervention in our study was targeted extensively to the Hindustani-Surinamese population[20], and the dieticians who performed the individual lifestyle counselling were all trained according to recommended protocols and instructed to monitor the participation of all those included in the study. For these reasons, despite the modest results of previous intervention studies in clinical practice but given the culturally targeted study design and the ethnic background of our population, we find it remarkable that we found neither changes in metabolic profile over time within the control and intervention groups, nor a difference in change over time between these groups. Given this discrepancy, we believe that understanding the effects of motivational factors and behavioural changes in the light of the lack of effect of our intervention, as well as a detailed process evaluation of the experiences in practice, will be of the utmost importance.

We do wish to mention that, although the lifestyle intervention was not effective in our overall population, we did find that participants who achieved a relatively large weight loss after 1 year (i.e. those within the highest quartile of weight loss), regardless whether they had been assigned to the control group or the intervention group, showed more improvement in lipid profile and glucose metabolism than participants in the lowest quartile of weight loss (supplementary material; Table S2). This emphasises that, if high-risk individuals in this population lose weight, there is a potential health gain.

At the same time, the low initial response rate and laborious recruitment [19], the high drop–out rate, and the lack of effect of the lifestyle intervention on weight change and other metabolic parameters raise the question whether our strategy is the optimal approach to prevent type 2 diabetes in our target population. Apart from fighting already existing metabolic disturbances with lifestyle interventions (i.e. in a high-risk approach), we might achieve additional health gain at by also focusing on prevention strategies that tackle the high risk of type 2 diabetes among South Asians in an even earlier stage [29].

All in all, we found that an intensive, culturally targeted lifestyle intervention did not result in improvement of weight status and metabolic profile after 1 year for South Asian individuals with a high risk of developing type 2 diabetes. Our results show that realising a health benefit by means of an intensive lifestyle intervention among a high risk South Asian population in general practice is extremely difficult and that additional preventive strategies may have to be considered in order to achieve the intended health gain.

## Supporting Information

Checklist S1 **Consort statement.**
(DOC)

Protocol S1 **Description of full study protocol in English.**
(PDF)

Protocol S2 **Full trial protocol after changes in design of study (in Dutch, 2012).**
(DOC)

Protocol S3 **Full trial protocol before start study (in Dutch, 2008).**
(DOC)

Protocol S4 **Copy trial protocol approval by ethics committee before changes to the protocol part 1.**
(PDF)

Protocol S5 **Copy trial protocol approval by ethics committee before changes to the protocol part 2.**
(PDF)

Protocol S6 **Copy trial protocol approval by ethics committee after changes to the protocol part 1.**
(PDF)

Protocol S7 **Copy trial protocol approval by ethics committee after changes to the protocol part 2.**
(PDF)

Table S1 **Difference in baseline characteristics between participants with and without follow-up data available after 1** **year.**
(DOC)

Table S2 **Comparison of change in metabolic parameters between those with high and low weight loss, defined by the highest versus lowest quartiles of weight change.**
(DOC)
