# Supplementary material for: Intensive Lifestyle Intervention in General Practice to Prevent Type 2 Diabetes among 18 to 60-Year-Old South Asians: 1-Year Effects on the Weight Status and Metabolic Profile of Participants in a Randomized Controlled Trial
Source: PLoS One. 2013 Jul 22;8(7):e68605. doi: 10.1371/journal.pone.0068605 (PMC3718785; doi:10.1371/journal.pone.0068605)
Supplement: Checklist S1 — Consort statement. (DOC) [file pone.0068605.s001.doc]

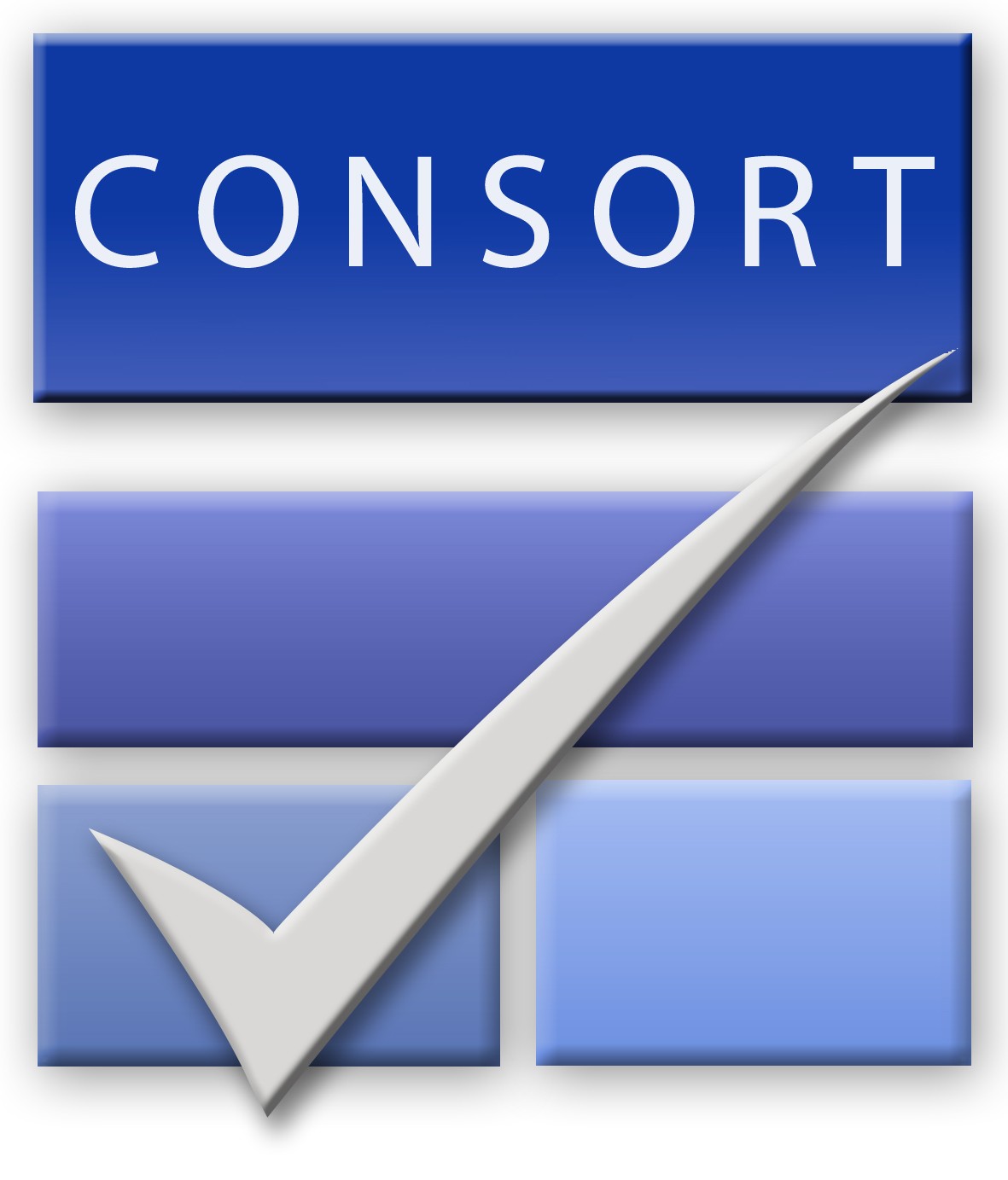
CONSORT 2010 checklist of information to include when reporting a randomised trial*

| Section/Topic | Item No | Checklist item | Reported in: |
| --- | --- | --- | --- |
| Title and abstract | | | |
|  | 1a | Identification as a randomised trial in the title | Title |
|  | 1b | Structured summary of trial design, methods, results, and conclusions (for specific guidance see CONSORT for abstracts) | Abstract |
| Introduction | | | |
| Background and objectives | 2a | Scientific background and explanation of rationale | Introduction |
|  | 2b | Specific objectives or hypotheses | Introduction |
| Methods | | | |
| Trial design | 3a | Description of trial design (such as parallel, factorial) including allocation ratio | Methods: sections ‘Study population’ + Inclusion in the trial |
|  | 3b | Important changes to methods after trial commencement (such as eligibility criteria), with reasons | Methods: Section ‘Main outcomes’ |
| Participants | 4a | Eligibility criteria for participants | Methods: section ‘Inclusion in the trial’ |
|  | 4b | Settings and locations where the data were collected | Methods: section ‘Study population’ |
| Interventions | 5 | The interventions for each group with sufficient details to allow replication, including how and when they were actually administered | Methods: Sections ‘Intervention group’ + ‘Control group’ + ‘Data collection |
| Outcomes | 6a | Completely defined pre-specified primary and secondary outcome measures, including how and when they were assessed | Methods: section ‘Main Outcomes’ |
|  | 6b | Any changes to trial outcomes after the trial commenced, with reasons | Methods: section ‘Main Outcomes’ |
| Sample size | 7a | How sample size was determined | Methods: section ‘Power calculation’ |
|  | 7b | When applicable, explanation of any interim analyses and stopping guidelines | N/A |
| Randomisation: |  |  |  |
| Sequence generation | 8a | Method used to generate the random allocation sequence | Methods: Section ‘Inclusion in the trial’ |
|  | 8b | Type of randomisation; details of any restriction (such as blocking and block size) | Methods: Section ‘Inclusion in the trial’ |
| Allocation concealment mechanism | 9 | Mechanism used to implement the random allocation sequence (such as sequentially numbered containers), describing any steps taken to conceal the sequence until interventions were assigned | Methods: Section ‘Inclusion in the trial’ |
| Implementation | 10 | Who generated the random allocation sequence, who enrolled participants, and who assigned participants to interventions | Methods: Section ‘Inclusion in the trial’ |
| Blinding | 11a | If done, who was blinded after assignment to interventions (for example, participants, care providers, those assessing outcomes) and how | Methods: Section ‘Data collection’ |
|  | 11b | If relevant, description of the similarity of interventions | N/A |
| Statistical methods | 12a | Statistical methods used to compare groups for primary and secondary outcomes | Methods: Section ‘Statistical analysis. |
|  | 12b | Methods for additional analyses, such as subgroup analyses and adjusted analyses | Methods: Section ‘Statistical analysis. |
| Results | | | |
| Participant flow (a diagram is strongly recommended) | 13a | For each group, the numbers of participants who were randomly assigned, received intended treatment, and were analysed for the primary outcome | Methods: Section Inclusion in the trial, Figure 1 |
|  | 13b | For each group, losses and exclusions after randomisation, together with reasons | Methods: Section ‘Statistical analysis. |
| Recruitment | 14a | Dates defining the periods of recruitment and follow-up | Methods: Section ‘Data Collection’ |
|  | 14b | Why the trial ended or was stopped | N/A |
| Baseline data | 15 | A table showing baseline demographic and clinical characteristics for each group | Results: Table 1 |
| Numbers analysed | 16 | For each group, number of participants (denominator) included in each analysis and whether the analysis was by original assigned groups | Methods: section Statistical analysis. Results: All Tables |
| Outcomes and estimation | 17a | For each primary and secondary outcome, results for each group, and the estimated effect size and its precision (such as 95% confidence interval) | Results:Table 2, Table 3a and Table 3b |
|  | 17b | For binary outcomes, presentation of both absolute and relative effect sizes is recommended | Results:Table 2, Table 3a and Table 3b |
| Ancillary analyses | 18 | Results of any other analyses performed, including subgroup analyses and adjusted analyses, distinguishing pre-specified from exploratory | Results:Table 2, Table 3a and Table 3b.  Supplementary Data: Table S1 and Table S2 |
| Harms | 19 | All important harms or unintended effects in each group (for specific guidance see CONSORT for harms) | N/A |
| Discussion | | | |
| Limitations | 20 | Trial limitations, addressing sources of potential bias, imprecision, and, if relevant, multiplicity of analyses | Discussion: section ‘Limitations of the study’ |
| Generalisability | 21 | Generalisability (external validity, applicability) of the trial findings | Discussion |
| Interpretation | 22 | Interpretation consistent with results, balancing benefits and harms, and considering other relevant evidence | Discussion |
| Other information | | |  |
| Registration | 23 | Registration number and name of trial registry | Abstract |
| Protocol | 24 | Where the full trial protocol can be accessed, if available | Abstract |
| Funding | 25 | Sources of funding and other support (such as supply of drugs), role of funders | Not in manuscript upon request PLOS one |

*We strongly recommend reading this statement in conjunction with the CONSORT 2010 Explanation and Elaboration for important clarifications on all the items. If relevant, we also recommend reading CONSORT extensions for cluster randomised trials, non-inferiority and equivalence trials, non-pharmacological treatments, herbal interventions, and pragmatic trials. Additional extensions are forthcoming: for those and for up to date references relevant to this checklist, see [www.consort-statement.org](http://www.consort-statement.org/).
