# Supplementary material for: Intensive Lifestyle Intervention in General Practice to Prevent Type 2 Diabetes among 18 to 60-Year-Old South Asians: 1-Year Effects on the Weight Status and Metabolic Profile of Participants in a Randomized Controlled Trial
Source: PLoS One. 2013 Jul 22;8(7):e68605. doi: 10.1371/journal.pone.0068605 (PMC3718785; doi:10.1371/journal.pone.0068605)
Supplement: Protocol S1 — Description of full study protocol in English. (PDF) [file pone.0068605.s002.pdf]

Vlaar et al. BMC Public Health 2012, 12:371
http://www.biomedcentral.com/1471-2458/12/371

BMC Public Health

STUDY PROTOCOL Open Access

# Feasibility and effectiveness of a targeted diabetes prevention program for 18 to 60-year-old South Asian migrants: design and methods of the DH!AAN study

Everlina MA Vlaar[1*], Irene GM van Valkengoed[1], Vera Nierkens[1], Mary Nicolaou[1], Barend JC Middelkoop[2,3] and Karien Stronks[1]

## Abstract

**Background:** South Asian migrants are at particularly high risk of type 2 diabetes. Previous studies have shown that intensive lifestyle interventions may prevent the onset of diabetes. Such interventions have not been culturally adapted and evaluated among South Asians in industrialized countries. Therefore, we have set up a randomized controlled trial to study the effectiveness of a targeted lifestyle intervention for the risk of type 2 diabetes and cardiovascular risk factors among 18 to 60-year-old Hindustani Surinamese (South Asians) in The Hague, the Netherlands. Here we present the study design and describe the characteristics of those recruited.

**Methods:** Between May 18, 2009 and October 11, 2010, we screened 2307 Hindustani Surinamese (18–60 years old) living in The Hague. We sent invitations to participate to those who had an impaired fasting glucose of 5.6-6.9 mmol/l, an impaired glucose tolerance of 7.8-11.0 mmol/L, a glycated hemoglobin level of 6.0% or more and/or a value of 2.39 or more for the homeostasis model assessment of estimated insulin resistance. In total, 536 people (56.1% of those eligible) participated. People with a higher level of education and a family history of type 2 diabetes were more likely to participate. The control and intervention groups were similar with regard to important background characteristics. The intervention group will receive a culturally targeted intervention consisting of dietary counseling using motivational interviewing and a supervised physical activity program. The control group will receive generic lifestyle advice. To determine the effectiveness, a physical examination (anthropometrics, cardiorespiratory test, lipid profile, and measures of oral glucose tolerance, glycated hemoglobin, and insulin) and interview (physical activity, diet, quality of life, and intermediate outcomes) were carried out at baseline and will be repeated at 1 year and 2 years. The process and the costs will be evaluated.

**Discussion:** This trial will provide insight into the feasibility and effectiveness of a targeted, intensive, lifestyle intervention for the risk of type 2 diabetes and cardiovascular risk factors among 18 to 60-year-old South Asians.

**Trial registration:** Dutch Trial Register: NTR1499

* Correspondence: E.M.Vlaar@amc.uva.nl
[1]Department of Public Health, Academic Medical Center, University of Amsterdam, Amsterdam, The Netherlands
Full list of author information is available at the end of the article

BioMed Central

© 2012 Vlaar et al.; licensee BioMed Central Ltd. This is an Open Access article distributed under the terms of the Creative Commons Attribution License (http://creativecommons.org/licenses/by/2.0), which permits unrestricted use, distribution, and reproduction in any medium, provided the original work is properly cited.

## Background

Type 2 diabetes mellitus (DM) is one of the most common chronic diseases in the industrialized countries [1]. The burden of DM is expected to increase due to factors such as aging, urbanization, and the increasing prevalence of obesity and physical inactivity [1].

South Asian migrants and their offspring living in industrialized countries (henceforth "South Asians") are at particularly high risk of DM [1–4]. In the Netherlands for example, the prevalence among the Hindustani Surinamese, who are of South Asian origin, is about four times as great as that among the ethnic Dutch in the same age group [4]. South Asians are not only younger at presentation, they are also at high risk of developing complications [3–6]. Hence, prevention of new DM cases and DM-related morbidity among South Asians potentially leads to an important health gain [7].

Previous studies have shown that intensive lifestyle interventions prevent the onset of DM among older populations with pre-diabetes [8–10]. Yet, while it seems particularly relevant, such intensive lifestyle interventions have not been implemented among South Asians in industrialized countries. There are two reasons. First, information about the feasibility and effectiveness of such interventions is lacking. This is particularly important, as South Asians are less likely to participate in intervention programs than their European counterparts [7,11,12]. Second, these interventions have not culturally targeted South Asians, while cultural adaptations likely have great influence on the effectiveness of interventions among specific populations [13,14].

Therefore, we want to study the effectiveness of a targeted, intensive, lifestyle intervention concerning the risk of DM and cardiovascular risk factors among 18 to 60-year-old Hindustani Surinamese (South Asians) in The Hague, the Netherlands. We will also evaluate the process and costs of the intervention to determine whether the targeted intervention program was feasible and implemented as planned. This paper a) outlines the protocol for the recruitment and a 2-year randomized controlled trial (RCT) and b) describes an analysis of the participation in the RCT and the baseline characteristics of the participants.

## Methods/design

In this paper, we describe the protocol of the RCT. The original protocol was adjusted during the study period because the recruitment period was extended, but the end date of the study was fixed. The changes in the original protocol are described in the Online Supplement (Additional file 1).

### Study design

This study was set up as an RCT with one intervention group and one control group (Figure 1). We invited potential participants for a screening to determine their risk of DM. If DM was suspected, the person concerned was referred to the family physician for further diagnostic testing and treatment. Those who appeared to be at "high risk of DM" were invited to participate in the RCT. We used a computer-generated randomization list to randomly assign invitees who gave their informed

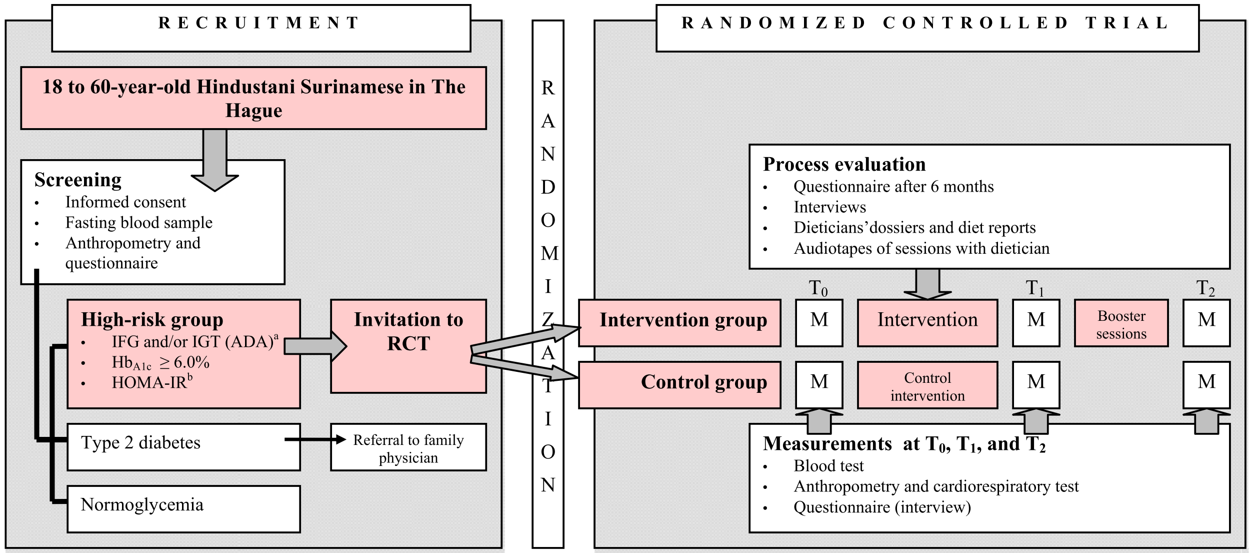


**Figure 1 Design of the DH!AAN study.** Legend: [a]FPG of 100–125 mg/dl (5.6–6.9 mmol/l) and/or 2-h postload glucose of 140–199 mg/dl (7.8–11.0 mmol/l), [b]HOMA-IR ≥ 2.39. ADA, American Diabetes Association; FPG, fasting plasma glucose; IFG, impaired fasting glucose; IGT, impaired glucose tolerance; $Hb_{A1C}$, glycated hemoglobin; HOMA-IR, homeostasis model assessment of estimated insulin resistance; M, measurements; RCT, randomized controlled trial.

consent to the intensive lifestyle intervention or the control group. Family members ($n = 38$), that is, participants belonging to the same household, defined as having the same postal code and house number, were assigned to the same program.

The main goal was to compare the risk of DM and several cardiovascular risk factors in the intervention group and the control group over time. The primary outcomes are changes in weight, mean blood glucose concentration, and behavioral change [diet and physical activity (PA)]. The secondary outcomes are cardiovascular risk factors, the use of primary health care and intermediate behavioral outcomes. Therefore, measurements were carried out at baseline and will be repeated after 1 and 2 years. The Medical Ethics Committee of the Academic Medical Center of Amsterdam, The Netherlands, has approved the study.

## Recruitment of the study population

### *Study population and invitation procedure*

The study population consisted of Hindustani Surinamese persons. The term "Hindustani Surinamese" refers to people of South Asian ancestral origin and their offspring who migrated to the Netherlands via Suriname. The Hindustani Surinamese are the descendants of the labourers from North India - Uttar Pradesh, Uttaranchal, and West Bihar – who were indentured between 1873 and 1917. The two large migration waves of Hindustani Surinamese to the Netherlands were caused mainly by the political situation in Suriname. The first wave took place at the time of the independence of Suriname in 1975, and the second wave, at the time of Desi Bouterse's coup in February 1980 [15].

We selected 10,583 Hindustani Surinamese (South Asians), aged 18–60 years, from 48 family physician lists in The Hague by means of name analysis. We chose the age range of 18 to 60 years because of the high prevalence of DM among 35 to 44-year-old South Asians in the Netherlands [4]. The researcher, the family physician or the practice nurse, and a trained research assistant of Hindustani Surinamese origin verified the names. We excluded people known to have DM and pregnant women (Figure 2). We chose to recruit via the family physician because of evidence that ethnic minority groups in the Netherlands in general appear to have confidence in their family physician ([16,17] and unpublished data). Moreover, we assumed that working through health care providers would facilitate future implementation.

The family physicians sent each potential participant an invitation letter with a reply card that could be returned if further contact was unwanted. Family members and other volunteers ($n = 163$) could also make an appointment for the screening. We culturally tailored the invitation by using the colors of the Surinamese flag and a Hindustani eye in the logo of the study, by citing a key figure encouraging participation, and by using the study name 'DH!AAN' – an acronym that means "attention" in Sarnami Hindustani, the Surinamese variant of Hindi. Moreover, we used plain language in the letter and leaflet, and we adjusted the risk information about DM to the Hindustani Surinamese population.

We sent a written reminder to invitees who had not responded to the invitation within 2 weeks and invited them to make an appointment. The reminder also said that if no response was received (no appointment or reply card) within 1 week, the invitee would be contacted by telephone. The study team phoned those who had not responded on at least three occasions (attempts varying in the afternoon and in the evening). If no telephone number was available (as was the case for 24.1% of the potential participants) or potential participants could not be reached, we sent a second written reminder asking people to contact us. This intensive recruitment strategy was tested during the pilot study and proved to be feasible and to achieve a higher response rate than only written invitations and reminders (unpublished data).

### *Initial screening*

The initial screening took place between May 18, 2009 and November 11, 2010. All participants ($n = 2307$) in the screening were asked to donate a fasting blood sample. We asked them to answer a brief questionnaire, which asked about their self-identified ethnicity (including the country of birth of the participants and of their parents), education level, their own DM status and that of first- and second-degree relatives, and previous medical diagnoses. Trained research staff carried out physical examinations in a standardized protocol in which they measured weight, height, and blood pressure (Table 1). All measurements were obtained twice.

The participants who were invited and screened between May 18, 2009 and April 18, 2010 ($n = 968$) took an oral glucose tolerance test (OGTT; 75 g). The physical examination for these participants also included measurements of body fat and waist and hip circumference. Moreover, four blood samples were collected for storage (Table 1).

### *Inclusion and exclusion criteria*

If the person's fasting glucose was impaired [a fasting plasma glucose (FPG) level of 5.6-6.9 mmol/l], glucose tolerance was impaired (2-h postload of 7.8-11.0 mmol/l), the hemoglobin $(Hb)_{A1c}$ was 6.0% or more, and/or the value for the homeostasis model assessment of estimated insulin resistance (HOMA-IR) was 2.39 or more [18–21], then the person was considered "at high risk of DM" and

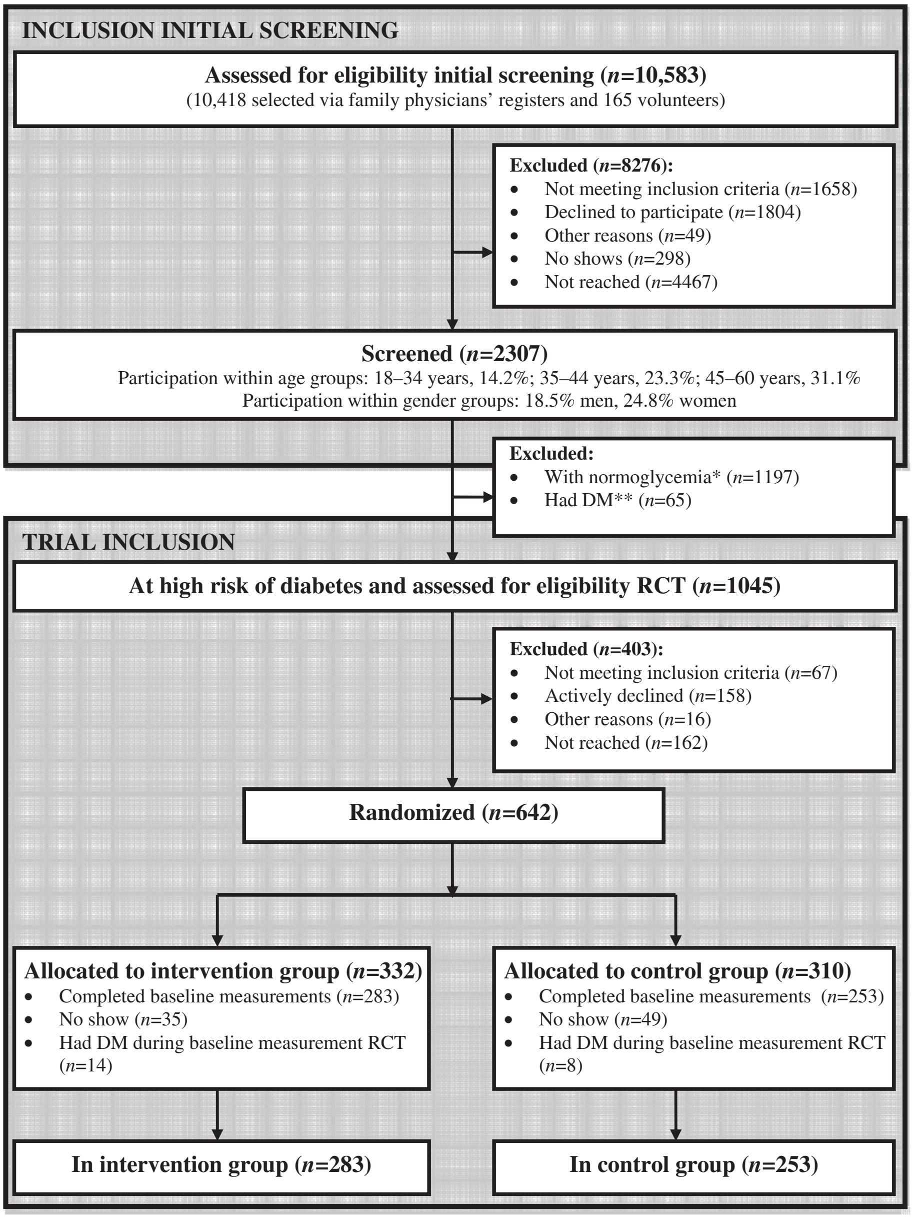


**Figure 2 Flow diagram from eligibility assessment to inclusion in the trial.** Legend: *Including n = 129 classified as normoglycemia during initial screening due to original protocol and n = 7 classified as normoglycemia during the baseline measurement and prior to randomization due to original protocol (Additional file 1); **Including n = 2 classified as DM during the baseline measurement and prior to randomization due to original protocol (Additional file 1); "High risk of DM" refers to having a fasting plasma glucose of 100–125 mg/dl (5.6 – 6.9 mmol/l) and/or 2-h postload glucose of 140–199 mg/dl (7.8–11.0 mmol/l); DM, type 2 diabetes mellitus; RCT, randomized controlled trial.

was eligible for the RCT. The threshold for HOMA-IR is based on the median value for HOMA-IR of the first 349 participants in the screening and is comparable to the median among people with DM of 2.4, as Matthews et al. (1985) find ([21] and unpublished data).

We asked potential participants for their consent (oral and written) and excluded anyone who was already involved in a lifestyle program, was pregnant, had a chronic disease that made participation in the intervention impossible, and/or used drugs interfering with plasma glucose levels. We evaluated this condition on a case-by-case basis.

### *Response and participation*

**Participation in the initial screening** Of all those who received an invitation, 2307 (21.8%) participated in the screening (Figure 2): 18.5% of the men and 24.8% of the

**Table 1 Measurements among participants in the RCT**

| Measurements | Method or sample used | Screening - recruitment period | RCT | | | | | |
|---|---|---|---|---|---|---|---|---|
| | | | Baseline ($T_0$) | 6 Months | 1 year ($T_1$) | | 2 years ($T_2$) | |
| | | | | | SC | HV | SC | HV |
| **I. Laboratory assessments** | | | | | | | | |
| 1.Fasting plasma glucose, $Hb_{A1c}$, insulin | Fasting venous blood sample | x | x | | x | d | x | d |
| 2.2-h Postload glucose | 2-h postload glucose | b | x | | x | | x | |
| 3.Triglycerides, HDL, LDL, cholesterol, creatinine, hs-CRP | Fasting venous blood sample | | x | | x | | x | |
| 4.Albumin and creatinine (urine) | Fasting urine sample | x | x | | x | x | x | x |
| 5.Blood sample storage (serum, EDTA) | Fasting venous blood sample | b | c | | | | x | |
| **II.Anthropometry** | | | | | | | | |
| 6.Weight, height, blood pressure (Omron M5-1) | SOP: twice, blood pressure measured 5 times | x | x | | x | x | x | x |
| 7. Waist and hip circumferences, fat percentage (Omron 500) | SOP: twice | b | x | | x | | x | |
| **III. Behavior** | | | | | | | | |
| 8.PA (SQUASH [22] adapted to SA [23]) | Questionnaire (interview) | | x | | x | x | x | x |
| 9.Diet (based on guideline for healthful diet) | Questionnaire (interview) | x | x | | x | x | x | x |
| 10.Smoking [24] | Questionnaire (interview) | x | x | | x | x | x | x |
| 11.Alcohol [25] | Questionnaire (interview) | x | x | | x | x | x | x |
| **IV. Other measurements[a]** | | | | | | | | |
| 12.Ethnicity, household information | Questionnaire | x | | | | | | |
| 13.Education, income, insurance, religion [26] | Questionnaire (interview) | education | x | | x | x | x | x |
| 14.Physical health (cardiovascular risk profile; [27]) | Questionnaire (interview) | f | g | | g | g | g | g |
| 15.Cardio respiratory test (Chester STEP test; [28,29]) | SOP | | x | | x | | x | |
| 16.Perceived quality of life (SF-15; [30,31]) | Questionnaire (interview) | | x | | x | x | x | x |
| 17.Distress (part of 4DSQ; [32]) | Questionnaire (interview) | | x | | x | x | x | x |
| 18.Risk perception ([33] and our own questionnaire) | Questionnaire (interview) | | x | x | x | x | x | x |
| 19.Attitude, knowledge, social support, self-efficacy, and stages of change in PA and diet | Questionnaire (interview) | | x | | x | x | x | x |
| 20.Perception of one's own body | Questionnaire (interview) | | x | | x | x | x | x |
| **V. Process** | | | | | | | | |
| 21.Dose received (participation in intervention components, frequency, leaflet, satisfaction) | Questionnaire (interview) | | | x | | | | |
| **VI. Costs** | | | | | | | | |
| 1.Use of health care | Questionnaire (interview) | | x | | x | x | x | x |
| 2.Costs of screening and lifestyle intervention | Questionnaire (interview) | h | x | | x | x | x | x |

Legend: [a]Questionnaire developed for the study; b – participants until April 19th, 2010 (n = 968); c – participants since April 19th, 2010; d – only $Hb_{A1c}$ measurement; f – heart disease, hypertension, high cholesterol, artritis and diabetes; g – heart failure and stroke; h – only costs screening; *SC*, screening; *HV*, home visit; *SOP*, standard operating procedures; *PA*,physical activity; *HDL*, high-density lipoprotein; *hs-CRP*, highly sensitive C-reactive protein; *LDL*, low-density lipoprotein; *PA*, physical activity; *SA*, South Asian(s); *SQUASH,* Short Questionnaire to Assess Health-Enhancing Physical Activity; *4DSQ*, Four-Dimensional Symptom questionnaire.

women. The participation rate was higher among those aged 45–60 years (31.1%) than among those aged 18–34 years (14.2%) or 35–44 years (23.3%). The net participation rate was 25.8% (2307 of those eligible (n = 8925)).

**Participation in the randomized controlled trial** In total, 536 people (283 intervention group; 253 control group) were included in the RCT (Figure 2). Of all the people considered at high risk of DM ($n = 1045$), 158

(15.1%) actively declined to participate. Moreover, 89 people (8.5%) were found not eligible; 67 prior to the randomization and 22 during the baseline measurement because of suspected DM. The net participation rate in the trial was 56.1% (536 of those eligible (n = 956)).

The characteristics of the participants, as measured in the initial screening, were comparable with the characteristics of the nonparticipants, with the exception of family history of DM and education (Table 2). Participants more often reported having a family member with DM than nonparticipants (chi-squared test, $p = 0.043$). Moreover, the participants had a somewhat higher level of education than the nonparticipants (Mann–Whitney $U$ test, $p = 0.023$). Nonparticipants seemed to have a higher blood pressure and were more likely to be classed as having elevated fasting glucose, but this was not significant.

#### *Baseline characteristics of the participants*

The median age of the participants was 45 years [interquartile range (IQR): 37–52] and 265 participants (49.4%) were men (Table 3). Most participants had an intermediate education level and had relatives in the first or second degree with DM. The median body mass index (BMI) was 26.9 kg/m$^2$ (IQR: 24.6–29.7), and the mean waist circumference was 95.0 cm (SD: 10.9) for men and 90.7 cm (SD: 10.5) for women. Moreover, the mean FPG level was 5.3 mmol/l (SD: 0.5), the median 2-h postload plasma glucose level was 5.8 mmol/l (IQR: 4.8–6.9) and the median $Hb_{A1c}$ level was 5.7% (IQR: 5.4–5.9). No significant differences were found between the intervention group and the control group (Table 3).

### Intervention group: The design of the lifestyle intervention

The aim of the lifestyle intervention is to meet the current guidelines for diet and PA [34,35]. We used data from previous qualitative and quantitative research within the Department of Public Health to adjust the intervention design, which was based on the design of the intervention in the SLIM study, to the situation in The Hague and the Hindustani Surinamese population [36–38].

The intervention consists of culturally targeted lifestyle counseling and a supervised PA program (exercise on prescription). Trained dieticians will conduct the lifestyle counseling, which consists of individual sessions and an optional program comprising a family session and cooking classes. The combination of diet and PA is often used in intensive lifestyle programs and appears to be more effective in preventing DM than diet or PA alone [8,9,39]. To target the diabetes prevention program to the Hindustani Surinamese population, we have added both surface and deep structure adaptations to make the intervention attractive, appropriate, and ultimately more effective [14]. The colors and key figures in the leaflet are examples of using the superficial characteristics of a cultural group; they can be seen as surface-structure adaptations [14]. Deep structure adaptations are adaptations targeted to factors that influence health behavior [14]. Paying specific attention to social pressure and support is an example of a deep structure adaptation in this study.

#### *Individual lifestyle counseling*

The individual lifestyle counseling consists of six to eight sessions in the 1st 6 months, followed by 3 to 4 booster sessions in the next 1.5 years, and it aims at both diet and PA. We use motivational interviewing (MI) as a counseling strategy, taking into account dietary habits and cultural norms regarding diet and PA. Motivational interviewing can be defined as “a client-centered, directive method for enhancing intrinsic motivation to change by exploring and resolving ambivalence” [40]. Motivational interviewing is an upcoming intervention methodology, and the evidence for MI for lifestyle advice seems to be promising [41,42].

The six dieticians who will conduct the lifestyle counseling are familiar with the Hindustani Surinamese culture and dietary habits. The medical psychologist who trained the dieticians in MI is an expert in MI [43]. Special attention will be paid to possible barriers and stimulating factors that are specific to the Hindustani Surinamese population. As recommended, we will provide continued support for MI to achieve optimal results in appliance and effects [44].

#### *Family session and cooking classes*

Besides the individual sessions, participants will also be offered a family session with the dietician. The purpose of this session is to decrease the social pressure to eat unhealthily and to increase the social support for a healthful lifestyle for the family. This entails providing product information and discussing the importance of a healthful lifestyle and how to increase support.

The participants will be offered two cooking classes to increase their self-efficacy in skills that they can learn for adjusting traditional dishes in a more healthful way. They will be encouraged to bring spouses or close relatives with them to increase their knowledge and attitude towards healthful diet.

#### *Exercise on prescription*

The intervention group will be offered “exercise on prescription” (EoP), a 20-week supervised PA program, which has previously been described [45–47]. The participants can choose between aquarobics and fitness, offered in men-only or women-only groups [45,46]. Trained PA coaches will monitor and supervise the participation. At the end of the 20 weeks, a final evaluation

**Table 2 Determinants of participation for those eligible for the trial**

| | All eligible people | Participants | Nonparticipants | Significance* |
|---|---|---|---|---|
| | n = 956^ | n = 536 | n = 420 | |
| Median age in years | 44 (36–51) | 45 (37–52) | 44 (35–51) | 0.13 |
| Men (%) | 468 (49.0) | 265 (49.4) | 203 (48.3) | 0.73 |
| 18–34 years (%) | 212 (22.2) | 109 (20.3) | 103 (24.5) | |
| 35–44 years (%) | 280 (29.3) | 159 (29.7) | 121 (28.8) | |
| 45–60 years (%) | 464 (48.5) | 268 (50.0) | 196 (46.7) | |
| Education (%) | | | | 0.02** |
| Elementary[a] | 126 (13.6) | 61 (11.7) | 65 (16.1) | |
| Intermediate[b] | 645 (69.8) | 364 (70.0) | 281 (69.6) | |
| University or equivalent[c] | 153 (16.6) | 95 (18.3) | 58 (14.4) | |
| Relatives with DM (%)[d] | 685 (73.7) | 398 (76.2) | 287 (70.3) | 0.04** |
| Median weight in kg | 73.5 (64.8–82.0) | 73.3 (65.0–82.1) | 73.8 (64.0–82.1) | 0.90 |
| Median BMI in kg/m$^2$ | 26.8 (24.4–29.9) | 26.8 (24.5–29.7) | 26.7 (24.1–30.3) | 0.98 |
| Overweight: ≥ 23 kg/m$^2$ (%)[e] | 419 (44.9) | 246 (46.9) | 173 (42.3) | 0.17 |
| Obesity: ≥ 27.5 kg/m$^2$ (%)[e] | 412 (44.1) | 227 (43.2) | 185 (45.2) | 0.54 |
| Median SBP in mm Hg | 128.0 (119.0–38.0) | 127.0 (118.0–137.0) | 129.0 (120.0–0.0) | 0.07 |
| Median DBP in mm Hg | 83.0 (76.0–90.0) | 82.0 (76.0–90.0) | 84.0 (76.0–92.0) | 0.06 |
| Hypertension (%)[f] | 62 (38.4) | 196 (37.1) | 166 (40.1) | 0.34 |
| Median FPG (mmol/l) | 5.3 (4.9–5.7) | 5.3 (4.9–5.7) | 5.4 (5.0–5.7) | 0.61 |
| Elevated FPG[†](%) | 332 (34.8) | 174 (32.5) | 158 (37.9) | 0.08 |
| Median $Hb_{A1c}$ in percentage | 5.7 (5.4–6.0) | 5.7 (5.4–6.0) | 5.7 (5.4–5.9) | 0.36 |
| Elevated $Hb_{A1c}$: ≥ 6.0% (%) | 238 (25.1) | 138 (25.8) | 100 (24.2) | 0.57 |
| Median HOMA in mmol/mol | 3.2 (2.6–4.4) | 3.2 (2.5–4.3) | 3.3 (2.7–4.4) | 0.36 |
| HOMA-IR: ≥ 2.39 (%) | 800 (84.0) | 453 (84.5) | 347 (83.4) | 0.65 |

Determinants measured during initial screening only; only determinants measured in total population are shown Data are *n* (percentages, calculated excluding missings), means (SD) if normally distributed or medians (IQRs) if not normally distributed ^All people labeled as 'at high risk of diabetes' (*n* = 1045) minus people who were found not eligible for the trial (*n* = 67 prior to randomization and *n* = 22 because of suspected DM during baseline measurement) *$p$ calculated with chi-squared test (binary data), independent *t*-test (continuous data), and Mann–Whitney *U* test (ordinal/nominal data and not normal distributed data).
**$p < 0.05$ – chi-squared test (Relatives with DM)/Mann–Whitney *U* test (Education).
[a]Primary education or less.
[b]Low vocational training, lower secondary education, intermediate vocational training and higher secondary education.
[c]Higher vocational training or university.
[d]First and second grade.
[e]BMI of 23–27.5 kg/m$^2$ was considered overweight; a BMI of 27.5 kg/m$^2$ or more, obesity [48].
[f]SBP ≥140 mm Hg and/or of DBP ≥ 90 mm Hg or if hypertension medication.
[g]American Diabetes Association definition: fasting glucose value of 100–125 mg/dl (5.6–6.9 mmol/l).
*BMI*, body mass index; *DBP*, diastolic blood pressure; *DM*, type 2 diabetes mellitus; *FPG*, fasting plasma glucose; *$Hb_{A1c}$*, glycated hemoglobin; *HOMA-IR*, homeostasis model assessment of estimated insulin resistance; *IQR*, interquartile range; *SBP*, systolic blood pressure.

will take place, at which time the lifestyle advisor will provide information about low-budget sport locations.

### Control group

The participants in the control group will be invited to join two group sessions led by student dieticians (after baseline measurements and after 6 months). The student dieticians will provide generic information about diabetes, discuss the current guidelines for diet and PA, list methods for achieving the recommended PA, and present some cases. In the second session, one respondent will be given the opportunity to become a case study. In addition, the control group will receive two flyers (at 3 months and 9 months) with simple, generic lifestyle advice. Both flyers offer participants a chance to phone in during preset hours to obtain personal advice from a dietician.

### Data collection

For both the intervention group and the control group, the baseline ($T_0$) and subsequent measurements after 1 ($T_1$) and 2 years ($T_2$) of the trial include blood sampling [fasting and 2-hour OGTT (glucose load 75 g)], a structured face-to-face interview, and a physical examination.

**Table 3 Baseline characteristics of the participants**

| | Participants | Intervention group | Control group | Significance* |
|---|---|---|---|---|
| | ***n* = 536** | ***n* = 283** | ***n* = 253** | |
| Median age in years | 45 (37–52) | 45 (37–52) | 44 (37–51) | 0.49 |
| Men (%) | 265 (49.4) | 136 (48.1) | 129 (51.0) | 0.50 |
| Education (%) | | | | 0.36 |
| Elementary[a] | 61 (11.7) | 27 (9.9) | 34 (13.7) | |
| Intermediate[b] | 364 (70.0) | 192 (70.6) | 172 (69.4) | |
| University or equivalent[c] | 95 (18.3) | 53 (19.5) | 42 (16.9) | |
| Family with DM (%)[d] | 398 (76.2) | 214 (77.8) | 184 (74.5) | 0.37 |
| Median BMI in kg/m$^2$ | 26.9 (24.5–29.6) | 26.9 (24.7–29.5) | 27.0 (24.4–29.8) | 0.90 |
| Mean waist circumference in cm | | | | |
| Men | 95.0 (10.9) | 95.0 (10.5) | 95.0 (11.4) | 0.99 |
| Women | 90.7 (10.5) | 90.4 (10.6) | 91.1 (10.4) | 0.60 |
| Median SBP in mm Hg | 125.5 (117.0–37.3) | 125.7 (117.5–7.5) | 125.0 (116.5–36.5) | 0.53 |
| Mean DBP in mm Hg | 83.0 (10.0) | 83.3 (9.9) | 82.7 (10.2) | 0.51 |
| Hypertension (%)[el] | 200 (38.6) | 105 (38.3) | 95 (38.9) | 0.89 |
| Plasma glucose in mmol/l | | | | |
| Mean FPG | 5.3 (0.5) | 5.3 (0.6) | 5.3 (0.5) | 0.77 |
| Median 2-h postload glucose | 5.8 (4.8–6.9) | 5.9 (5.0–7.0) | 5.5 (4.8–6.9) | 0.12 |
| Median $Hb_{A1c}$ in % | 5.7 (5.4–5.9) | 5.7 (5.4–5.9) | 5.6 (5.4–5.9) | 0.31 |
| Median HOMA-IR | 3.1 (2.2–4.1) | 3.1 (2.2–4.2) | 3.0 (2.3–4.1) | 0.91 |
| Median insulin in pmol/l | 12.9 (9.6–17.3) | 13.4 (9.6–17.5) | 12.8 (9.6–17.1) | 0.86 |
| Mean LDL in mmol/l | 3.2 (0.9) | 3.2 (0.8) | 3.2 (0.9) | 0.83 |
| Median HDL in mmol/l) | 1.2 (1.0–1.4) | 1.2 (1.1–1.4) | 1.2 (1.0–1.4) | 0.27 |

Data are given as *n* (percentages, calculated excluding missings), means (SD) if normally distributed or medians (IQRs) if not normally distributed.
**p* is given as calculated with the chi-squared test (binary data), independent *t*-test (continuous data), or Mann–Whitney *U* test (ordinal/nominal data and not normally distributed data).
[a]Primary education or less.
[b]Low vocational training, lower secondary education, intermediate vocational training and higher secondary education.
[c]Higher vocational training and university.
[d]First and second grade.
[e]Systolic blood pressure ≥ 140 mm Hg and/or diastolic blood pressure ≥ 90 mm Hg or if hypertension medication.
*BMI*, body mass index; *DBP*, diastolic blood pressure; *DM*, type 2 diabetes mellitus; *FPG*, fasting plasma glucose; *$Hb_{A1c}$*, glycated hemoglobin; *HDL*, high-density lipoprotein; *HOMA-IR*, homeostasis model assessment of estimated insulin resistance; *IQR*, interquartile range; *LDL*, low-density lipoprotein; *SBP*, systolic blood pressure; *SD*, standard deviation.

These actions all took place, and will take place again, in one central health care center in The Hague. The participants will receive a questionnaire after 6 months as part of the process evaluation. Participants who do not fill in the questionnaire at 6 months will be asked to complete it at $T_1$ in the health care center. Table 1 shows the measures recorded at baseline and to be recorded again after 6 months, 1 year, and 2 years. Participants who were not reached and any participants who indicate that they intend to quit the trial at $T_1$ or $T_2$ will be offered a brief home visit instead of the more extensive screening at the health care center (Table 1).

### *Laboratory assessments*

Plasma glucose, insulin, and $Hb_{A1c}$ levels and other biomarkers will be assessed (Table 1). All laboratory assessments will be carried out according a standardized protocol at the General Practice Laboratory Foundation, Etten-Leur, The Netherlands. At different time points, material is stored at −80°C for future analyses (Table 1).

### *Physical examination*

Trained research staff carries out the physical examinations in a standardized protocol. A Seca mechanical scale (Seca 761, Seca, Hamburg, Germany) is used to weigh the participants (wearing light clothing) to the nearest 500 g. Height is recorded to the nearest 0.01 m on a Seca portable stadiometer (Seca 214, Seca, Hamburg, Germany), and waist and hip circumferences are determined to the nearest 0.01 m with a tape measure. A bioelectrical impedance analysis (OMRON BF500, Omron Healthcare, Hoofddorp, The Netherlands) is used to

determine body fat to the nearest 0.1%. All anthropometric measurements are obtained twice – we allowed differences between two measurements of 2 cm, 2 kg, or 2% – and the means will be used for analysis.

The participant is in the seated position when the blood pressure (Omron M5-1, Omron Healthcare, Hoofddorp, The Netherlands) is measured on the non-dominant arm supported at heart level. At most, five measurements are taken. We calculate the mean from the first two measurements with less than 5 mm Hg difference in both systolic and diastolic blood pressure [49]. We assess cardiorespiratory fitness with the Chester STEP test [28,29].

#### *Structured face-to-face interview*

**Behavioral outcomes** We will assess PA with the Short Questionnaire to Assess Health-enhancing Physical Activity (SQUASH), supplemented with ethnicity-specific activities [22,23]. Dietary intake will be determined with questions based on the national guideline for a healthful diet, supplemented with questions about specific dietary habits of the Hindustani Surinamese population [34,38]. We will assess fruit, vegetables, rice, and bread consumption with multi-item measurements to determine the quantity and frequency ($n = 3$, $n = 2$, $n = 2$, and $n = 11$, respectively), and two single-item questions will address the use of breakfast and the regularity of the meals. We will also measure the current smoking and alcohol consumption (Table 1) [24,25].

**Intermediate outcome measures** Health-related quality of life will be measured with short form (SF)-12 and three extra emotional status questions from SF-36 [30,31]. We will measure distress with the 16 distress questions of the Four-Dimensional Symptom Questionnaire (distress, depression, anxiety, and somatization) [32].

We will measure personal vulnerability with a three-item personal vulnerability score [33] and risk perception with 12 statements about the participant's perceived risk of diabetes in connection with overweight, current smoking, and/or unhealthy eating. The statements are based on general risk factors and information from focus group discussions [37,50].

We will determine attitudes towards PA and diet directly with questions about importance and joy regarding several aspects of PA and diet (e.g., PA in leisure time; breakfast; fruit, vegetable and whole grain intake). We will assess attitude towards PA indirectly with seven items about the possible consequences of increasing PA (e.g., "If I increase my PA, I will look better").

We will ask whether the spouse, other family members, or close relatives encourage the respondent to be physically active ($n = 3$). Moreover, we will measure overall support towards the targeted aspects of diet ($n = 7$).

We will measure self-efficacy expectations towards diet change and dealing with specific PA barriers with five items about PA and seven items about aspects of diet [focus group discussions; unpublished data].

The stage of change towards PA and diet will be measured with the algorithm described in the Transtheoretical Model [51]. This includes three scales regarding motivation to change, described as: unmotivated to change diet and PA within 6 months, motivated to change at least parts of the diet and PA within 6 months, and prepared to change within 1 month [52].

**Background characteristics** We will assess physical health, religion, and socioeconomic status (as indicated by educational level, source of income, and net family income) using questions similar to those of the SUNSET study [26,27].

### Activities to encourage participation in the data collection

We will send all participants the DH!AAN study newsletter twice a year in order to inform them of the progress of the study and to encourage continued participation. The participants were rewarded with a gift coupon of €5 to €10 for their participation in the baseline measurements; this reward will be repeated for subsequent measurements. Moreover, a prize in the form of a trip to Suriname will be raffled to one of the participants of the $T_1$ and $T_2$ measurements.

### Statistical analysis

#### *Size of the study population and the power calculation*

The Online supplement (Additional file 1) describes the original power calculation. Here we calculate the power of the study to demonstrate differences in the main outcomes using the numbers of participants in the intervention and control groups.

A total of 536 participants were included in the RCT with a minimum of 253 participants per group. Previous studies found a difference in weight reduction of 1.5–3 kg (SD of 4) [10,53], and a difference in FPG of 0.2–0.35 mmol/l (SD of 0.7) [10,53]. Assuming a an alpha of 5%, and a dropout rate of 30%, we have a power of 86% or more to demonstrate a minimum difference in weight reduction of 1.25 kg and a change in glucose level of 0.2 mmol/l with our data.

#### *Effectiveness of the intensive lifestyle intervention*

Statistical analysis will be performed according to the intention-to-treat principle and, if necessary, corrected for differences in background characteristics between the intervention and control groups. To assess the effect

of the intervention program on the risk of diabetes and cardiovascular risk factors after 1 and 2 years, we will calculate differences between the intervention and control groups in general linear mixed models (GLMM). If we observe a difference between the intervention and control groups, we will examine whether the magnitude of the effect is most likely due to other cardiovascular risk factors or behavioral change. We will also use GLMM to examine the influence of baseline variables and the degree of participation (e.g., adherence and the actual carrying out of the components of the intervention) in weight reduction and behavioral changes. We will use multilevel analyses to distinguish group effects (e.g., effect of dietician's work) from individual effects.

#### *Process evaluation*

In the process evaluation we will investigate for each intervention component fidelity, dose received (exposure and satisfaction) and dose delivered [54]. Several measures will be used including a structured questionnaire at six months, semi-structured interviews with a sample of participants and non-participans, dieticians' files and audiotapes of the counseling sessions of participants with dieticians.

We will measure fidelity with questions in the questionnaire and semi-structured interview about relevance, understandability, and satisfaction with each component of both lifestyle interventions. We will determine the level of MI applied from the audiotapes using One Pass, which is based on the Motivational Interviewing Skill Code scoring list [55]. We will assess the level of cultural sensitivity during the MI sessions with a scoring list specially designed for this study. We based it on a combination of the Seeleman and colleagues' cultural sensitivity model and diverse existing scoring lists for cultural sensitivity [56–58].

We will measure measure the dose received (exposure) and the dose delivered by asking participants about their participation in each component of the intervention and analysing the dieticians' registration files.

#### *Evaluation of the costs*

We developed questions in collaboration with a health economist to determine the costs. We asked participants to note their travel time, mode of transportation, travel costs, and hours of work missed for the screening in the recruitment phase and at $T_0$, but will only ask about any change in transportation at $T_1$ and $T_2$ (Table 1). We will measure costs for the intervention program in the questionnaire at 6 months; for example, the time invested, the cost of the change of diet, and the cost of participating in an exercise program (e.g., sportswear; Table 1). Moreover, to measure whether the screening and intervention affected the use of primary health care, we will ask participants about their use of certain health care, laboratory assessments, and medication at $T_0$, $T_1$, and $T_2$ (Table 1).

## Discussion

Our study is unique in terms of population (South Asians in industrialized countries), age (young population), the mode of recruitment, and the culturally targeted design of the intervention. No studies have reported on the feasibility and potential effectiveness of an intensive lifestyle intervention in this population, while South Asians in industrialized countries are at high risk of DM and are therefore an important target group. Given this high risk of DM, we used broad criteria for inclusion. For instance, in contrast with previous studies, we did not restrict participation to those with overweight. We selected participants on the basis of high levels of $Hb_{A1c}$ and HOMA-IR as these markers have been independently associated with diabetes risk [19,20,59,60]. Using $Hb_{A1c}$ as a marker for DM and people at high risk of DM is in line with recent recommendations [19,61].

This strategy is illustrative for the design of our trial, which incorporates both pragmatic and explanatory elements according to the definitions of Roland and Togerson [62]. Because of this, we expect to be able to provide information how and why the intervention works, but also insight in whether this intervention may work in real life conditions.

Despite the lack of specific evidence, results from earlier studies suggest that DM screening and intervention among South Asians – with a culturally sensitive approach – may be effective and lead to a decrease in the burden of DM and risk of cardiovascular disease [7,13,63,64]. For instance, one study in India has reported an effect of a lifestyle intervention on the incidence of DM. However, given the substantial differences in context, their results are not generalizable to South Asians living in industrialized countries [64].

### Limitations

Our study has some limitations that should be mentioned. First, participation in the initial screening and at baseline was relatively low, despite our intensive and targeted recruitment strategy. The participation rate is often low in studies among ethnic minority groups in western countries [7,12,65,66]. Our initial screening had a higher participation rate than another study that was partially targeted to this population [7].

The low participation may be associated with selection bias. We cannot rule this out in our study. As in previous studies, participation in the initial screening was highest among women and older people [8,67,68]. Moreover, participation at baseline was greater among people with a

higher education level and among those who reported having family with diabetes than among those who did not. This may be related to the fact that well-educated people or people who have a family history of DM are more motivated to engage in preventive behavior [66–69]. At the same time, other background characteristics of the participants and nonparticipants were comparable. Nevertheless, we should keep in mind that selection bias may affect the generalizability of our findings.

Another limitation of the study is that, during its course, we were forced to shorten the duration of the follow-up to 2 years, instead of maintaining the originally intended 3 years. As a consequence, the focus on the incidence of type 2 diabetes shifted to other outcome measures. We acknowledge that this limits the assessment of the effectiveness for the prevention of incident DM. However, we believe that our findings will still provide valuable insight into feasibility and will indicate the potential health gain that can be achieved.

Changes in several relevant outcomes, e.g., diet and physical activity, will be assessed by means of questionnaires. However, self-reported behavior may be subject to recall bias and social desirability [70]. This can be partly overcome if we combine these self-reported data with an assessment of more objective measures intended to provide insight into the effectiveness [71].

In summary, this study will contribute to the evidence base for lifestyle interventions for the prevention of DM. Specifically, the trial will provide insight into the potential effectiveness of a targeted intensive lifestyle intervention among 18 to 60-year-old South Asians in an industrialized country who form a population at high risk of DM. During the 2-year study we will assess the changes in a broad range of relevant outcomes. Moreover, we expect that the evaluation of the process and costs will provide important information about the feasibility of a culturally targeted lifestyle intervention among South Asians via general practices and other health care providers.

## Additional file

**Additional file 1: Online supplement.** Changes to the original protocol of the randomized controlled trial [2,8,36,53,64].

### Competing interests
The authors declare that they have no competing interests.

### Authors' contributions
EV analyzed the data, contributed to the interpretation, and drafted the manuscript. IV, VN, and KS contributed to the design of the study and the interpretation of the data. They also reviewed and edited the manuscript. BM and MN gave advice for the design and interpretation and reviewed the manuscript. All authors read and approved the final manuscript.

### Acknowledgements
The Netherlands Organization for Health Research and Development (ZonMw) funded the trial with grant (projectnumber ZonMw: ZonMw 120620023).

### Author details
[1]Department of Public Health, Academic Medical Center, University of Amsterdam, Amsterdam, The Netherlands. [2]Department of Public Health and Primary Care, Leiden University Medical Center, Leiden, The Netherlands. [3]Public Health Service, The Hague, The Netherlands.

Received: 22 March 2012 Accepted: 2 April 2012
Published: 23 May 2012

### References
1. Wild S, Roglic G, Green A, Sicree R, King H: **Global prevalence of diabetes: estimates for the year 2000 and projections for 2030.** *Diabetes Care* 2004, **27:**1047–1053.
2. Kanaya AM, Wassel CL, Mathur D, Stewart A, Herrington D, Budoff MJ, *et al*: **Prevalence and correlates of diabetes in South asian indians in the United States: findings from the metabolic syndrome and atherosclerosis in South asians living in america study and the multi-ethnic study of atherosclerosis.** *Metab Syndr Relat Disord* 2010, **8:**157–164.
3. Gholap N, Davies M, Patel K, Sattar N, Khunti K: **Type 2 diabetes and cardiovascular disease in South Asians.** *Prim Care Diabetes* 2011, **5:**45–56.
4. Bindraban NR, van Valkengoed IG, Mairuhu G, Holleman F, Hoekstra JB, Michels BP, *et al*: **Prevalence of diabetes mellitus and the performance of a risk score among Hindustani Surinamese, African Surinamese and ethnic Dutch: a cross-sectional population-based study.** *BMC Public Health* 2008, **8:**271.
5. Bos V, Kunst AE, Keij-Deerenberg IM, Garssen J, Mackenbach JP: **Ethnic inequalities in age- and cause-specific mortality in The Netherlands.** *Int J Epidemiol* 2004, **33:**1112–1119.
6. Mangalmurti SS, Paley A, Gany F, Fisher EA, Hochman JS: **South Asians and risk of cardiovascular disease: current insights and trends.** *Ethn Dis* 2010, **20:**474–478.
7. Webb DR, Gray LJ, Khunti K, Srinivasan B, Taub N, Campbell S, *et al*: **Screening for diabetes using an oral glucose tolerance test within a western multi-ethnic population identifies modifiable cardiovascular risk: the ADDITION-Leicester study.** *Diabetologia* 2011, **54:**2237–2246.
8. Knowler WC, Barrett-Connor E, Fowler SE, Hamman RF, Lachin JM, Walker EA, *et al*: **Reduction in the incidence of type 2 diabetes with lifestyle intervention or metformin.** *N Engl J Med* 2002, **346:**393–403.
9. Tuomilehto J, Lindstrom J, Eriksson JG, Valle TT, Hamalainen H, Ilanne-Parikka P, *et al*: **Prevention of type 2 diabetes mellitus by changes in lifestyle among subjects with impaired glucose tolerance.** *N Engl J Med* 2001, **344:**1343–1350.
10. Roumen C, Corpeleijn E, Feskens EJ, Mensink M, Saris WH, Blaak EE: **Impact of 3-year lifestyle intervention on postprandial glucose metabolism: the SLIM study.** *Diabet Med* 2008, **25:**597–605.
11. Wandell PE, Carlsson A, Steiner KH: **Prevalence of diabetes among immigrants in the Nordic countries.** *Curr Diabetes Rev* 2010, **6:**126–133.
12. Mason S, Hussain-Gambles M, Leese B, Atkin K, Brown J: **Representation of South Asian people in randomised clinical trials: analysis of trials' data.** *BMJ* 2003, **326:**1244–1245.
13. Hawthorne K, Robles Y, Cannings-John R, Edwards AG: **Culturally appropriate health education for type 2 diabetes mellitus in ethnic minority groups.** *Cochrane Database Syst Rev* 2008, **:**CD006424.
14. Resnicow K, Soler R, Braithwaite RL: **Cultural sensitivity in substance use prevention.** *Journal of community psychology* 2000, **28:**271–290.
15. Choenni C, Harmsen C: **Place of birth and ethnic composition of the Surinamese in the Netherlands [in Dutch].** *Bevolkingtrends* 2007, **1:**74–78.
16. Beune EJ, Haafkens JA, Agyemang C, Schuster JS, Willems DL: **How Ghanaian, African-Surinamese and Dutch patients perceive and manage antihypertensive drug treatment: a qualitative study.** *J Hypertens* 2008, **26:**648–656.
17. Beune EJ, Haafkens JA, Agyemang C, Bindels PJ: **Inhibitors and enablers of physical activity in multiethnic hypertensive patients: qualitative study.** *J Hum Hypertens* 2010, **24:**280–290.
18. **Diagnosis and classification of diabetes mellitus.** *Diabetes Care* 2007, **30** (Suppl 1):S42–S47.

19. International Expert Committee report on the role of the A1C assay in the diagnosis of diabetes. *Diabetes Care* 2009, **32**:1327–1334.
20. Song Y, Manson JE, Tinker L, Howard BV, Kuller LH, Nathan L, *et al*: **Insulin sensitivity and insulin secretion determined by homeostasis model assessment and risk of diabetes in a multiethnic cohort of women: the Women's Health Initiative Observational Study.** *Diabetes Care* 2007, **30**:1747–1752.
21. Matthews DR, Hosker JP, Rudenski AS, Naylor BA, Treacher DF, Turner RC: **Homeostasis model assessment: insulin resistance and beta-cell function from fasting plasma glucose and insulin concentrations in man.** *Diabetologia* 1985, **28**:412–419.
22. Wendel-Vos GC, Schuit AJ, Saris WH, Kromhout D: **Reproducibility and relative validity of the short questionnaire to assess health-enhancing physical activity.** *J Clin Epidemiol* 2003, **56**:1163–1169.
23. de Munter JS, van Valkengoed IG, Agyemang C, Kunst AE, Stronks K: **Large ethnic variations in recommended physical activity according to activity domains in amsterdam, the netherlands.** *Int J Behav Nutr Phys Act* 2010, **7**:85.
24. Stivoro: **Meetinstrumenten voor onderzoek naar roken en stoppen met roken.** 2006, Ref Type: Online Source.
25. Centraal Bureau voor Statistiek: **Gezondheidsenquete POLS 2008.** 2008, Ref Type: Online Source.
26. Dijkshoorn H, van Dijk TK, Janssen AP: *Eindrapport Amsterdamse Gezondheidsmonitor 2008*. Ref Type: Online Source; 2009.
27. ROSE GA: **The diagnosis of ischaemic heart pain and intermittent claudication in field surveys.** *Bull World Health Organ* 1962, **27**:645–658.
28. Sykes K, Roberts A: **The Chester step test-a simple yet effective tool for the prediction of aerobic capacity.** *Physiotherapy* 2004, **90**:183–188.
29. Buckley JP, Sim J, Eston RG, Hession R, Fox R: **Reliability and validity of measures taken during the Chester step test to predict aerobic power and to prescribe aerobic exercise.** *Br J Sports Med* 2004, **38**:197–205.
30. Mols F, Pelle AJ, Kupper N: **Normative data of the SF-12 health survey with validation using postmyocardial infarction patients in the Dutch population.** *Qual Life Res* 2009, **18**:403–414.
31. Aaronson NK, Muller M, Cohen PD, Essink-Bot ML, Fekkes M, Sanderman R, *et al*: **Translation, validation, and norming of the Dutch language version of the SF-36 Health Survey in community and chronic disease populations.** *J Clin Epidemiol* 1998, **51**:1055–1068.
32. Terluin B, van Marwijk HW, Ader HJ, de Vet HC, Penninx BW, Hermens ML, *et al*: **The Four-Dimensional Symptom Questionnaire (4DSQ): a validation study of a multidimensional self-report questionnaire to assess distress, depression, anxiety and somatization.** *BMC Psychiatry* 2006, **6**:34.
33. Claassen L, Henneman L, van der Weijden T, Marteau T, Timmermans D: *Being at risk for cardiovascular disease: perceptions and preventive behaviour in people with and without known genetic predisposition*. Amsterdam: Vrije Universiteit; 2011.
34. Health Council of the Netherlands: *Guidelines for a healthy diet: the ecological perspective*. The Hague: Health Council of the Netherlands; 2011.
35. Kemper HCG, Ooijendijk WTM, Stiggelbout M: **Concensus over de Nederlandse norm voor gezond bewegen.** *Tijdschrift voor Gezondheidswetenschappen (TSG)* 2000, **78(3)**:180–183.
36. Mensink M, Feskens EJM, Saris WHM, de Bruin TW, Blaak EE: **Study on Lifestyle Intervention and Impaired Glucose Tolerance Maastricht (SLIM): preliminary results after one year.** *Int J Obes Relat Metab Disord* 2003, **27**:377–84.
37. Kohinor MJ, Stronks K, Nicolaou M, Haafkens JA: **Considerations affecting dietary behaviour of immigrants with type 2 diabetes: a qualitative study among Surinamese in the Netherlands.** *Ethn Health* 2011, **16**:245–258.
38. Nicolaou M, van Dam RM, Stronks K: **Acculturation and education level in relation to quality of the diet: a study of Surinamese South Asian and Afro-Caribbean residents of the Netherlands.** *J Hum Nutr Diet* 2006, **19**:383–393.
39. Greaves CJ, Sheppard KE, Abraham C, Hardeman W, Roden M, Evans PH, *et al*: **Systematic review of reviews of intervention components associated with increased effectiveness in dietary and physical activity interventions.** *BMC Public Health* 2011, **11**:119.
40. Miller WR, Rollnick S: *Motivational Interviewing, preparing people to change addictive behavior*. New York: The Guildford Press; 1991.
41. Rubak S, Sandbaek A, Lauritzen T, Christensen B: **Motivational interviewing: a systematic review and meta-analysis.** *Br J Gen Pract* 2005, **55**:305–312.
42. Brug J, Spikmans F, Aartsen C, Breedveld B, Bes R, Fereira I: **Training dietitians in basic motivational interviewing skills results in changes in their counseling style and in lower saturated fat intakes in their patients.** *J Nutr Educ Behav* 2007, **39**:8–12.
43. Broers S, Smets E, Bindels P, Evertsz' FB, Calff M, De HH: **Training general practitioners in behavior change counseling to improve asthma medication adherence.** *Patient Educ Couns* 2005, **58**:279–287.
44. van Eijk-Hustings YJ, Daemen L, Schaper NC, Vrijhoef HJ: **Implementation of Motivational Interviewing in a diabetes care management initiative in The Netherlands.** *Patient Educ Couns* 2011, **84**:10–15.
45. Schmidt M, Absalah S, Nierkens V, Stronks K: **Which factors engage women in deprived neighbourhoods to participate in exercise referral schemes?** *BMC Public Health* 2008, **8**:371.
46. Stichting Precura: **Exercise on Prescription [Bewegen op Recept].** 2010. Ref Type: Online Source.
47. Hosper K, Deutekom M, Stronks PK: **The effectiveness of "Exercise on Prescription" in stimulating physical activity among women in ethnic minority groups in the Netherlands: protocol for a randomized controlled trial.** *BMC Public Health* 2008, **8**:406.
48. **Appropriate body-mass index for Asian populations and its implications for policy and intervention strategies.** *Lancet* 2004, **363**:157–163.
49. The Working Group on Revised Hypertension Guidelines. Techniques of office blood pressure measurement. Revised Hypertension Guidelines. 37–38: *The Dutch Institute for Healthcare Improvement and the Dutch Heart Foundation*. Report: Ref Type; 2000.
50. Lindstrom J, Tuomilehto J: **The diabetes risk score: a practical tool to predict type 2 diabetes risk.** *Diabetes Care* 2003, **26**:725–731.
51. Prochaska JO, Johnson S, Lee P, Shumaker SAE, Schron EBE, Ockene JKE, *et al*: **The transtheoretical model of behavior change. The handbook of health behavior change**. In. 2nd edition Edited by. New York: Springer Publishing Co; 1998:59–84.
52. DiClemente CC, Prochaska JO, Fairhurst SK, Velicer WF, Velasquez MM, Rossi JS: **The process of smoking cessation: an analysis of precontemplation, contemplation, and preparation stages of change.** *J Consult Clin Psychol* 1991, **59**:295–304.
53. Lindstrom J, Eriksson JG, Valle TT, Aunola S, Cepaitis Z, Hakumaki M, *et al*: **Prevention of diabetes mellitus in subjects with impaired glucose tolerance in the Finnish Diabetes Prevention Study: results from a randomized clinical trial.** *J Am Soc Nephrol* 2003, **14**:S108–S113.
54. Saunders RP, Evans MH, Joshi P: **Developing a process-evaluation plan for assessing health promotion program implementation: a how-to guide.** *Health Promot Pract* 2005, **6**:134–147.
55. Resnicow K: **1-Pass coding system for motivational interviewing: introduction and scoring. 1–7.** In Edited by. Atlana: Rollins School of Public Health, Emory University; 2002. Ref Type: Report.
56. Seeleman C, Suurmond J, Stronks K: **Cultural competence: a conceptual framework for teaching and learning.** *Med Educ* 2009, **43**:229–237.
57. Goody CM, Drago L: **Using Cultural Competence Constructs to Understand Food Practices and Provide Diabetes Care and Education.** *Diabetes Spectrum* 2009, **22**.
58. Harris-Davis E, Haughton B: **Model for multicultural nutrition counseling competencies.** *J Am Diet Assoc* 2000, **100**:1178–1185.
59. Bennett CM, Guo M, Dharmage SC: **HbA(1c) as a screening tool for detection of Type 2 diabetes: a systematic review.** *Diabet Med* 2007, **24**:333–343.
60. Selvin E, Steffes MW, Zhu H, Matsushita K, Wagenknecht L, Pankow J, *et al*: **Glycated hemoglobin, diabetes, and cardiovascular risk in nondiabetic adults.** *N Engl J Med* 2010, **362**:800–811.
61. **Diagnosis and classification of diabetes mellitus.** *Diabetes Care* 2010, **33** (Suppl 1):S62–S69.
62. Roland M, Togerson DJ: **Understanding controlled trials: What are pragmatic trials?** *BMJ* 1998, **316**:285.
63. Douglas A, Bhopal RS, Bhopal R, Forbes JF, Gill JM, Lawton J, *et al*: **Recruiting South Asians to a lifestyle intervention trial: experiences and lessons from PODOSA (Prevention of Diabetes & Obesity in South Asians).** *Trials* 2011, **12**:220.
64. Ramachandran A, Snehalatha C, Mary S, Mukesh B, Bhaskar AD, Vijay V: **The Indian Diabetes Prevention Programme shows that lifestyle modification and metformin prevent type 2 diabetes in Asian Indian subjects with impaired glucose tolerance (IDPP-1).** *Diabetologia* 2006, **49**:289–297.

65. Bartlett C, Doyal L, Ebrahim S, Davey P, Bachmann M, Egger M, *et al*: **The causes and effects of socio-demographic exclusions from clinical trials.** *Health Technol Assess* 2005, **9:**iii–x.
66. El FF, Bruijnzeels MA, Foets MM, Hoes AW: **Different distribution of cardiovascular risk factors according to ethnicity: a study in a high risk population.** *J Immigr Minor Health* 2008, **10:**559–565.
67. Greenland P, Hildreth NG, Maiman LA: **Attendance patterns and characteristics of participants in public cholesterol screening.** *Am J Prev Med* 1992, **8:**159–164.
68. Blackwell CS, Foster KA, Isom S, Katula JA, Vitolins MZ, Rosenberger EL, *et al*: **Healthy Living Partnerships to Prevent Diabetes: recruitment and baseline characteristics.** *Contemp Clin Trials* 2011, **32:**40–49.
69. Claassen L, Henneman L, Nijpels G, Dekker J, Marteau T, Timmermans D: *Perceived control over diabetes risk and preventive behaviour: the role of family history and self-malleability*. Amsterdam: Vrije Universiteit; 2011:73–82.
70. Shephard RJ: **Limits to the measurement of habitual physical activity by questionnaires.** *Br J Sports Med* 2003, **37:**197–206.
71. Kremers SP, Visscher TL, Seidell JC, Van MW, Brug J: **Cognitive determinants of energy balance-related behaviours: measurement issues.** *Sports Med* 2005, **35:**923–933.

doi:10.1186/1471-2458-12-371
**Cite this article as:** Vlaar *et al.*: Feasibility and effectiveness of a targeted diabetes prevention program for 18 to
60-year-old South Asian migrants: design
and methods of the DH!AAN study. *BMC Public Health* 2012 **12**:371.

**Submit your next manuscript to BioMed Central and take full advantage of:**

- **Convenient online submission**
- **Thorough peer review**
- **No space constraints or color figure charges**
- **Immediate publication on acceptance**
- **Inclusion in PubMed, CAS, Scopus and Google Scholar**
- **Research which is freely available for redistribution**

Submit your manuscript at
www.biomedcentral.com/submit

BioMed Central
