# Supplementary material for: Intensive Lifestyle Intervention in General Practice to Prevent Type 2 Diabetes among 18 to 60-Year-Old South Asians: 1-Year Effects on the Weight Status and Metabolic Profile of Participants in a Randomized Controlled Trial
Source: PLoS One. 2013 Jul 22;8(7):e68605. doi: 10.1371/journal.pone.0068605 (PMC3718785; doi:10.1371/journal.pone.0068605)
Supplement: Protocol S3 — Full trial protocol before start study (in Dutch, 2008). (DOC) [file pone.0068605.s004.doc]

**De effectiviteit van een aangepast diabetes preventie programma voor Hindostaanse Surinamers, bestaande uit een screening gevolgd door een leefstijlinterventie**

***Verkorte titel: Diabetes preventie studie bij Hindostaanse Surinamers***

**Onderzoeksprotocol**

**Februari 2009**

**Versie 3**

E.M.A. Vlaar (1), MSc, dr. I.G.M. van Valkengoed(1), dr. V. Nierkens(1), Prof. dr. B. Middelkoop(2), Prof. Dr. K. Stronks(1),

*1. afdeling Sociale Geneeskunde, Academisch Medisch Centrum van de Universiteit van Amsterdam.*

*2. GGD Den haag, Leids Universitair Medisch Centrum*

**Inhoudsopgave**

[1. Inleiding 4](#__RefHeading___Toc221349488)

[2. Onderzoeksdoel en vraagstellingen 6](#__RefHeading___Toc221349489)

[2.1 Vraagstellingen 6](#__RefHeading___Toc221349490)

[3. Inhoud programma 7](#__RefHeading___Toc221349491)

[3.1 Screening 7](#__RefHeading___Toc221349492)

[3.1.1 Benadering voor eerste screening 7](#__RefHeading___Toc221349493)

[3.1.2 Uitnodiging voor tweede screening ter bevestiging van diagnose prediabetes 10](#__RefHeading___Toc221349494)

[3.1.3 Uitnodiging voor effectonderzoek 10](#__RefHeading___Toc221349495)

[3.2 Leefstijlinterventie 11](#__RefHeading___Toc221349496)

[3.2.1 Doel van de leefstijlinterventie 11](#__RefHeading___Toc221349497)

[3.2.2 Inhoud leefstijlinterventie 11](#__RefHeading___Toc221349498)

[3.2.2.a Beweeginterventie 12](#__RefHeading___Toc221349499)

[3.2.2.b Voedingsinterventie 14](#__RefHeading___Toc221349500)

[3.2.3 Vervolgcontacten 15](#__RefHeading___Toc221349501)

[3.2.4 Training en intervisie diëtisten 15](#__RefHeading___Toc221349502)

[3.3 Controle groep 15](#__RefHeading___Toc221349503)

[4. Design 17](#__RefHeading___Toc221349504)

[4.1 Onderzoeksdesign screening 17](#__RefHeading___Toc221349505)

[4.2 Onderzoeksdesign leefstijlinterventie 18](#__RefHeading___Toc221349506)

[4.3 Onderzoekspopulatie 18](#__RefHeading___Toc221349507)

[4.3.1 Onderzoekspopulatie screening 18](#__RefHeading___Toc221349508)

[4.3.2 Onderzoekspopulatie leefstijlinterventie 19](#__RefHeading___Toc221349509)

[4.4 Trajectdoorloop patiënten in de studie 20](#__RefHeading___Toc221349510)

[4.4.1 Natraject nieuw gedetecteerde diabeten 21](#__RefHeading___Toc221349511)

[4.5 Statistische power 21](#__RefHeading___Toc221349512)

[5. Dataverzameling screening en effectiviteitsonderzoek 22](#__RefHeading___Toc221349513)

[5.1 Eerste screening 22](#__RefHeading___Toc221349514)

[5.1.1 Vragenlijst 22](#__RefHeading___Toc221349515)

[5.1.2 Lichamelijk onderzoek 23](#__RefHeading___Toc221349516)

[5.1.3 OGTT 23](#__RefHeading___Toc221349517)

[5.2 Tweede screening, nameting na 12 maanden en 36 maanden 25](#__RefHeading___Toc221349518)

[5.2.1 Bepalingen in het lab 25](#__RefHeading___Toc221349519)

[5.2.2 Vragenlijst 25](#__RefHeading___Toc221349520)

[6. Analyse screening en effectonderzoek 27](#__RefHeading___Toc221349521)

[6.1 Screening 27](#__RefHeading___Toc221349522)

[6.2 Interventie 27](#__RefHeading___Toc221349523)

[6.3 Programma evaluatie 27](#__RefHeading___Toc221349524)

[6.3.1 Kosten 27](#__RefHeading___Toc221349525)

[Referenties 30](#__RefHeading___Toc221349526)

# 1. Inleiding

Type 2 diabetes is in Nederland een van de meest voorkomende chronische ziekten. In Nederland is de prevalentie van type 2 diabetes bij allochtonen hoger dan bij autochtonen. Vooral bij Hindostaanse Surinamers is de prevalentie veel hoger (1). Het SUNSET (Surinamese in the Netherlands – Study on health and EThnicity) onderzoek, recent uitgevoerd door het Academisch Medisch Centrum (AMC), heeft de risicofactoren voor hart- en vaatziekten in kaart gebracht bij Surinaamse Creolen, Hindostanen en autochtone Nederlanders. In de leeftijdsgroep van 45 tot 60 jaar heeft maar liefst 40% van de Hindostanen diabetes (2). Deze prevalentie ligt 3-6 keer hoger vergeleken met de autochtone bevolking. Ook in de leeftijdsgroep van 35-44 jaar had al 20% van de Hindostanen diabetes (2). Daarnaast blijkt uit andere internationale onderzoeken dat de gemiddelde leeftijd van Hindostanen jonger is als ze diabetes ontwikkelen (3), maar ook dat ze een groter risico hebben op het ontwikkelen van hart- en vaatziekten (4).

Patiënten met type 2 diabetes hebben een verhoogd risico op complicaties zoals, retinopatie, nefropatie, neuropatie en hart- en vaatziekten. Complicaties worden veroorzaakt door langdurig hoge bloedglucose waarden. Verhoogde bloedglucose waarden zijn gemiddeld al 10 jaar voordat de diagnose diabetes wordt gesteld aanwezig (5). Dit kan onherstelbare schade veroorzaken. Het doel van de behandeling bij diabetes is het verlagen van de bloedglucose waarden. Daarnaast zal ook een te hoge bloeddruk of cholesterolgehalte worden behandeld om het risico op hart- en vaatziekten te verlagen (6).

Op basis van deze gegevens lijkt systematische screening om type 2 diabetes vroeg op te sporen en te behandelen relevant. De Nederlandse gezondheidsraad concludeerde echter in een rapport dat screening mogelijk effectief is maar dat er nog te weinig bewijs is om algemene screening aan te bevelen (5). Voor screening op type 2 diabetes in hoog risicogroepen is het aannemelijk dat er gezondheidswinst te behalen valt (5). Zo wordt de hoge prevalentie van diabetes onder de Hindostaanse populatie in Nederland erkend als een groot algemeen gezondheidsprobleem.

Het verder onderzoeken van mogelijkheden om Hindostanen te screenen op diabetes type 2 lijkt van groot belang. De gezondheidswinst die de screening kan opleveren lijkt relatief groot te zijn gezien de jonge leeftijd waarop diabetes ontstaat en resulteert in een verhoogde risico op hart- en vaatziekten. Wel is het dan van belang om Hindostanen op een jongere leeftijd te screenen.

Screening op type 2 diabetes is er op gericht om de ziekte in een vroeg stadium op te sporen om zo complicaties te voorkomen of te verminderen. Bovendien kunnen tijdens de screening patiënten worden opgespoord die prediabetes (een voorstadium van diabetes) hebben. Bij deze personen is sprake van een verhoogd nuchter bloedglucose gehalte (IFG, impaired fasting glucose) of een vermindere glucose tolerantie (IGT, impaired glucose tolerance). Een vermindere glucose tolerantie kan gemeten worden met behulp van een orale glucose tolerantietest (OGTT). Ruim 30% van deze personen ontwikkelt binnen 5 jaar diabetes (7). Personen met prediabetes zouden zorg aangeboden moeten krijgen om het ontwikkelen van diabetes te voorkomen of uit te stellen. Het bevorderen van gezonde voeding en lichaamsbeweging zou hier een onderdeel van moeten zijn.

Secundaire preventie (het opsporen van mensen met prediabetes, een risicofactor voor het ontstaan van diabetes) heeft in eerdere preventieprogramma’s laten zien dat het effectief is in het voorkomen of uitstellen van diabetes (8, 9). Zowel het Amerikaanse Diabetes Preventie programma als het Finse Diabetes Preventie onderzoek resulteerde in een risicoverlaging van 58% op het ontwikkelen van diabetes (10, 11). Deze resultaten worden ook ondersteunt door de SLIM studie (12). Echter eerdere interventiestudies zijn alleen uitgevoerd onder een overwegend Caucasische populatie. Daarnaast laat eerder onderzoek zien dat interventies minder effectief zijn als zij niet zijn toegespitst op de betreffende doelgroep (13). De vraag is daarom of een leefstijlinterventie afgestemd op de Hindostaanse populatie even effectief in het verlagen van het risico op diabetes.

Maar ook de etiologie van diabetes in de Hindostaanse populatie vraagt de aandacht.

Een resultaat uit het SUNSET onderzoek is namelijk dat de verhoogde kans op diabetes niet geheel lijkt te kunnen worden verklaard door bekende risicofactoren zoals overgewicht en een verhoogde middelomtrek (2). Dit benadrukt het belang van een meer specifieke aanpak.

Tegen deze achtergrond is in een eerdere haalbaarheidsstudie een screeningsprogramma ontwikkeld dat specifiek gericht is op de kenmerken van de Hindostaanse populatie en op haalbaarheid onderzocht. Uit de evaluatie is gebleken dat een dergelijke screening haalbaar is en zowel diabetes als prediabetes kan worden opgespoord. Tevens is aan de hand van kwalitatieve en kwantitatieve onderzoeksgegevens een leefstijlinterventie ontwikkeld voor personen met prediabetes, die is aangepast op de Hindostaanse populatie.

In het huidige onderzoek zal de effectiviteit van dit programma, bestaande uit een de aangepaste screening en de aangepaste leefstijlinterventie, worden onderzocht.

Het onderzoek zal worden uitgevoerd onder de 18-60 jarige Hindostaans Surinaamse populatie in Den Haag.

Deze effectstudie zal worden uitgevoerd door de afdeling Sociale Geneeskunde van het AMC. Binnen de projectgroep wordt samengewerkt met andere afdelingen van het AMC en met de Gemeentelijke Gezondheidsdienst (GGD) in Den Haag en het Leids Universitair Medisch Centrum (LUMC). Met de verkregen informatie uit dit onderzoek zal zowel inzicht worden verkregen in de effectiviteit van een screening gevolgd door een leefstijlinterventie, als aanbevelingen worden gedaan voor de eventuele implementatie van een screening op diabetes onder Hindostanen gevolgd door een leefstijlinterventie.

# 2. Onderzoeksdoel en vraagstellingen

Het doel van dit onderzoek is het evalueren van de effectiviteit van een preventieprogramma speciaal afgestemd op de Hindostaanse populatie om prediabetes en type 2 diabetes in een vroeg stadium op te sporen en bij personen met prediabetes het ontstaan van type 2 diabetes mogelijk te voorkomen of uit te stellen. Het programma bestaat uit een screening bij Hindostanen in de leeftijd van 18-60 jaar welke gevolgd zal worden door een leefstijlinterventie om diabetes te voorkomen of uit te stellen. In dit hoofdstuk worden de onderzoeksvragen verder uitgewerkt.

## Vraagstellingen

Bovenstaand doel valt uiteen in de volgende subvragen:

1. Wat is de effectiviteit ten aanzien van het voorkomen of uitstellen van diabetes bij personen met prediabetes?

- Hoeveel personen met prediabetes of diabetes worden geïdentificeerd?
- Wat is de aan te bevelen leeftijdsrange voor screening?
- Wat is het aan te bevelen screeningsinterval (afhankelijk van de prevalentie van prediabetes en diabetes tijdens de screening en de incidentie van prediabetes en diabetes bij personen met normoglycemie bij de eerste screening)?
- Kunnen criteria gebaseerd op (biomedische) parameters gebruikt worden om personen met diabetes of prediabetes te identificeren?
- Wat is de effectiviteit van de leefstijlinterventie m.b.t. het voorkomen of uitstellen van diabetes bij personen met prediabetes?
- Wat is het effect van de leefstijlinterventie op intermediaire uitkomsten, zoals motivatie, voeding en lichamelijke activiteit?
- Wat is het effect van de interventie op secundaire uitkomstmaten, zoals bloeddruk, lipiden profiel en gewicht?
- Wat zijn de determinanten van het effect van de interventie op de primaire, intermediaire en secundaire uitkomsten?

1. Wat zijn de kosten versus de opbrengsten van het programma?

- Wat zijn de opbrengsten van het programma in termen van voorkomen gevallen van diabetes?
- Wat zijn de kosten per voorkomen case?
- Wat zijn de organisatorische vereisten van het programma?

1. Wat zijn de potentiële neveneffecten van het programma?

- Wat is het effect van de interventie op de kwaliteit van leven (o.a. ervaren stress) van personen met normoglycemie, prediabetes en diabetes?
- Is er een toename in het aantal huisartsbezoeken n.a.v. het programma?

# 3. Inhoud programma

In dit hoofdstuk zal de inhoud van het programma worden beschreven. Het eerste deel zal gaan over de screening. Het tweede deel gaat over de leefstijlinterventie.

## 3.1 Screening

Het doel van de screening is het opsporen van mensen met diabetes en met een verhoogde kans op diabetes (prediabetes). Om het screenings programma zo goed mogelijk aan te laten sluiten bij de Hindostaanse populatie wordt rekening gehouden met bepaalde aspecten die erg belangrijk zijn bij de ontwikkeling van diabetes bij Hindostanen. Zo zijn Hindostanen gemiddeld jonger op het moment dat diabetes gediagnosticeerd wordt. Uit het SUNSET onderzoek bleek dat de prevalentie van diabetes in de leeftijdscategorie van 35-45 jaar hoger is vergeleken met Nederlanders ouder dan 45 jaar (2). Daarbij zijn bloedglucose gehaltes gemiddeld al 10 jaar verhoogd voordat de diagnose diabetes wordt gesteld. Dit werd bevestigd door de uitkomsten van de haalbaarheidsstudie (niet gepubliceerd). Daarom zullen Hindostanen in de leeftijdscategorie van 18 tot en met 60 jaar geïncludeerd worden in de screening.

De verhoogde kans op het krijgen van diabetes bij Hindostanen kan niet worden toegeschreven aan risicofactoren zoals abdominaal overgewicht en leeftijd (2). Daarom is er voor gekozen dat iedereen gescreend wordt en er dus niet een preselectie plaatsvindt op basis van overgewicht en buikomvang. Dan bestaat de kans dat er patiënten gemist worden waardoor het effect van de screening minder wordt.

Het is van belang dat de respons en deelname aan programma zo hoog mogelijk is. Ervaringen uit het haalbaarheidsonderzoek laten zien dat een meer persoonlijke benadering nodig is om een goede respons te krijgen. Daarom wordt in deze vervolgstudie gebruik gemaakt een meer intensieve benadering, zoals ontwikkeld in de haalbaarheidsstudie.

### 3.1.1 Benadering voor eerste screening

*Uitnodigingsbrief*

Potentiële deelnemers ontvangen een uitnodigingsbrief namens de huisarts. Voor het versturen worden blanco enveloppen gebruikt. De uitnodiging bevat algemene informatie over het onderzoek en zal worden ondertekend door de huisarts, mede namens het onderzoeksteam. Tevens wordt een folder met informatie over het onderzoek, een aanbevelingsbrief van mw. R. Roopram (voormalig miss Nederland) en een antwoordkaartje toegevoegd. Respondenten worden gevraagd om binnen 2 weken een afspraak te maken om zich te laten screenen. Tevens staat er een telefoonnummer van het onderzoeksteam en een telefoonnummer van een onafhankelijk arts in de brief vermeld waar mensen naar toe kunnen bellen voor vragen.

Het antwoordkaartje kan worden ingestuurd indien men niet verder benaderd wenst te worden voor het onderzoek.

In de wachtkamer van de deelnemende huisarts zal een poster hangen om de patiënten te attenderen op het onderzoek. Op deze poster wordt ook aangegeven wat patiënten kunnen doen als zij niet mee willen doen.

*Herinneringsbrief*

Potentiële respondenten die niet binnen 2 weken reageren op de uitnodiging krijgen een herinneringsbrief toegestuurd, waarin nogmaals wordt gevraagd een afspraak te maken. Tevens wordt aangegeven dat het onderzoeksteam telefonisch contact zal proberen op te nemen. Dit gebeurt na ruim 1 week, wanneer de potentiële respondenten de gelegenheid hebben gehad om het antwoordkaartje te retourneren als zij niet benaderd willen worden. Indien geen telefoonnummer beschikbaar is, dan wordt een brief nagestuurd waarin de potentiële respondent wordt geïnformeerd dat het onderzoeksteam hem of haar niet kan bereiken. Ook potentiële respondenten die na 5 x telefonisch contact niet bereikt zijn zullen deze ‘niet-bereikt brief ontvangen.

*Afspraken*

Wanneer respondenten een afspraak hebben gemaakt krijgen ze een afsprakenbrief thuisgestuurd met de gegevens m.b.t. de afspraak en informatie over de gang van zaken bij de screening.

*Niet gekomen – nieuwe afspraak*

Wanneer een respondent op een geplande afspraak niet is komen opdagen zal de respondent gebeld worden om een nieuwe afspraak te maken. Wanneer de respondent niet telefonisch te bereiken is wordt er een brief gestuurd met een verzoek te bellen voor nieuwe afspraak.

*Uitslagenbrief*

De uitslagen van het onderzoek (glucosegehalte, gewicht, bloeddruk) zullen worden teruggerapporteerd aan de deelnemer en huisarts. Wanneer het relevant is dan zullen deelnemers aan de screening worden geadviseerd om contact op te nemen met de huisarts voor verdere diagnostiek en behandeling van diabetes een te hoge bloeddruk.

In figuur 1 is ter verduidelijking een stroomschema gemaakt van de benaderingsmethode.

1. Versturen uitnodigingsbrief

2. Respondent maakt afspraak

**Non respons**

Versturen herinneringsbrief

Versturen niet-bereikt brief

Respondent wordt gebeld

3. Afsprakenbrief versturen

4. SMS versturen

Respondent belt en wil mee doen

Respondent belt niet

Respondent stuurt antwoordkaart of respondent belt en doet niet mee

Respondent belt

**Non respons**

Respondent belt niet

Respondent bereikt en wil meedoen.

Respondent niet bereikt

Respondent belt niet

Respondent belt

Respondent belt niet

Respondent belt

Versturen 2e afsprakenbrief

Respondent verzet afspraak

5. Respondent wordt

gescreend

Respondent komt

Respondent komt niet

Respondent wordt gebeld

Versturen niet-bereikt

afsprakenbrief

**Non respons**

Respondent niet bereikt

Respondent belt niet

Respondent belt

Respondent maakt afspraak

2e Afsprakenbrief versturen

Respondent bereikt

SMS versturen

Respondent komt

Respondent komt niet

**Non respons**

6. Versturen uitslagbrief

Respondent bereikt, maar doet niet mee.

**Non respons**

Respondent stuurt antwoordkaart of respondent belt en doet niet mee

**Non respons**

*Figuur 1 Stroomschema benaderingsmethode respondent eerste screening*

### 3.1.2 Uitnodiging voor tweede screening ter bevestiging van diagnose prediabetes

*Uitnodiging*

Personen met een verhoogd glucosegehalte zullen worden uitgenodigd voor een tweede screening. Hiervoor is tijdens de eerste screening toestemming gevraagd. In de brief, gedrukt op briefpapier van het onderzoek, staat het doel en de inhoud van deze tweede screening toegelicht. Respondenten kunnen zelf contact opnemen voor een afspraak en worden tegelijk telefonisch benaderd met een verzoek tot deelname.

*Niet-bereikt brief*

Indien geen telefoonnummer beschikbaar of het nummer veranderd blijkt, dan wordt een brief nagestuurd waarin de potentiële respondent wordt geïnformeerd dat het onderzoeksteam hem of haar niet kan bereiken. Potentiële respondenten die niet binnen 2 weken reageren of telefonisch bereikt worden krijgen ook een niet-bereikt brief toegestuurd, waarin nogmaals wordt gevraagd een afspraak te maken.

*Afspraken*

Wanneer respondenten een afspraak hebben gemaakte krijgen ze een afsprakenbrief thuisgestuurd met de gegevens m.b.t. de afspraak en informatie over de gang van zaken bij de tweede screening.

*Niet gekomen – nieuwe afspraak*

Wanneer een respondent op een geplande afspraak niet is komen opdagen zal de respondent gebeld worden om een nieuwe afspraak te maken. Wanneer de respondent niet telefonisch te bereiken is wordt er een brief gestuurd met een verzoek te bellen voor nieuwe afspraak.

*Uitslagenbrief*

De uitslagen van het onderzoek (glucosegehalte, gewicht, bloeddruk, lipiden) zullen worden teruggerapporteerd aan de deelnemer en huisarts. Wanneer het relevant is dan zullen deelnemers aan de screening worden geadviseerd om contact op te nemen met de huisarts voor verdere diagnostiek en behandeling van diabetes of een te hoge bloeddruk.

### 3.1.3 Uitnodiging voor effectonderzoek

*Uitnodiging*

Personen die op basis van het gemiddelde van twee metingen (eerste en tweede screening) een verhoogd glucosegehalte in het bloed hebben, worden uitgenodigd voor een vervolgonderzoek. Hiervoor is tijdens de eerste screening toestemming gevraagd. In de uitnodiging staat het doel en de inhoud van het vervolgonderzoek toegelicht. Respondenten kunnen deelnemen door de bijgevoegde toestemmingsverklaring in te vullen en op te sturen. De deelnemers worden aan de hand van de opgestuurde informed consent willekeurig (enkel blind) toegewezen aan de controlegroep of de interventiegroep. Vervolgens worden zij telefonisch benaderd voor het maken van een afspraak met de diëtiste en beweegcoach (interventiegroep) of met de leefstijlconsulent (controlegroep). Respondenten die geen informed consent hebben opgestuurd worden gebeld voor hun mondelinge toestemming, waarna een huisbezoek volgt om de toestemmingsverklaring af te halen.

*Niet-bereikt brief*

Potentiële respondenten die niet binnen 2 weken bereikt worden krijgen een niet-bereikt brief toegestuurd, waarin nogmaals wordt gevraagd een afspraak te maken.

*Afspraken*

Wanneer respondenten een afspraak hebben gemaakt krijgen ze een afsprakenbrief thuisgestuurd met de gegevens m.b.t. de afspraak voor de intake bij de dietist en beweegcoach en informatie over de gang van zaken bij het vervolgonderzoek (metingen) en de interventie behorend bij het programma waar zij bij ingedeeld zijn.

*Niet gekomen – nieuwe afspraak*

Wanneer een respondent op de geplande intakes niet is komen opdagen zal de respondent gebeld worden om een nieuwe afspraak te maken. Wanneer de respondent niet telefonisch te bereiken is wordt er een brief gestuurd met een verzoek te bellen voor nieuwe afspraak.

## 3.2 Leefstijlinterventie

### 3.2.1 Doel van de leefstijlinterventie

De algemene doelen van de leefstijlinterventie zijn overeenkomstig aan de doelen van eerdere interventiestudies, zoals het Finse Diabetes Preventie Programma en de SLIM studie in Maastricht (8, 14). De doelen van de interventie zijn specifiek gemaakt voor de Hindostaanse populatie, gebaseerd op analyses uit het SUNSET onderzoek en gegevens uit de haalbaarheidsstudie (2, niet gepubliceerd). Voor de afkapwaarden voor de middelomtrek en BMI wordt de norm voor de Zuid-Aziatische populatie gehanteerd, volgens de richtlijnen van de WHO (15). De doelen van de interventie zijn als volgt:

1. Reductie van het lichaamsgewicht
   1. 5-7% gewichtsverlies voor deelnemers met een BMI >23 kg/m^2^ . Het doel voor deelnemers met een BMI ≤ 23 kg/m^2^ is het handhaven van een gezond gewicht.
   2. 5% verlaging van de middelomtrek voor vrouwen met een middelomtrek > 0.8 m en voor mannen met een middelomtrek van >0.9 m. Voor personen met een lagere middelomtrek geld het doel om de middelomtrek binnen de norm te houden.
2. Verbeteren van de kwaliteit van de voeding

Het doel is om te eten volgens de richtlijnen goede voeding met aandacht voor verzadigd vet, groente, fruit en vezels. Aanbevelingen zijn een goede balans tussen energie-inname en energieverbruik. Hierbij hoort een vetintake van 20-40 en% waarvan maximaal 10 en% verzadigd vet, tenminste 400 gram groente en fruit en een vezelinname van tenminste 3.4 gram per MJ (16).

1. Verhogen van de lichaamsbeweging

Alle deelnemers worden aangemoedigd om ten minste 30 min per dag, 5 dagen per week gematigd intensief te bewegen. Dit is conform de Nederlandse richtlijnen (17).

Resultaten uit het SUNSET onderzoek en de haalbaarheidsstudie laten zien dat deze interventie doelen relevant zijn voor de Hindostaanse populatie. Een groot deel van de Hindostaanse populatie voldoet niet aan de richtlijnen van gezonde voeding en beweging (2, 18, 19, niet gepubliceerd).

### 3.2.2 Inhoud leefstijlinterventie

Eerdere leefstijlinterventies zijn over het algemeen voor de blanke populatie ontwikkeld, zoals de U.S. Diabetes Preventie programma, het Finse diabetes preventie onderzoek en de Nederlandse SLIM studie (10-12). De haalbaarheidsstudie heeft laten zien dat de inhoud van deze interventies maar deels aansluiten bij de Hindostaanse populatie (niet gepubliceerd). Om de leefstijlinterventie specifiek te maken voor de Hindostaanse populatie moet er rekening worden gehouden met o.a. het verschil in risicofactoren op diabetes, voedingsgewoonten en voorkeuren voor bepaalde soorten lichamelijke beweging. Daarnaast zijn ook aandacht voor motivatie en determinanten van motivatie zoals attitude en eigen effectiviteit van belang bij de ontwikkeling van de interventie.

In de haalbaarheidsstudie is, rekening houdend met al deze factoren, onderstaande aangepaste interventie ontwikkeld voor deze doelgroep.

##

### 3.2.2.a Beweeginterventie

***Lichamelijke activiteit: ‘Bewegen op Recept’***

Iedere deelnemer die ingedeeld is in de interventiegroep wordt uitgenodigd voor het “Bewegen op Recept” programma (BOR), dat wordt georganiseerd door het STIOM (Stichting ter Ondersteuning van de Gezondheidszorg en Maatschappelijke dienstverlening in Den Haag) en gefinancierd door de gemeente in samenwerking met verzekeraars (20-22). Het programma is opgezet om lichamelijke activiteit te bevorderen onder bewoners van achterstandswijken in Den Haag.

Het hoofddoel van het programma is het verhogen van de betrokkenheid/empowerment van de deelnemer, het aanbieden van aangepaste activiteiten en het verhogen van de motivatie door persoonlijke begeleiding. Dit zijn belangrijke factoren die geassocieerd zijn met gedragsverandering (23). Daarnaast is aangetoond dat dit programma geschikt is voor de Hindostaanse doelgroep (22).

*Intake*

BOR is opgebouwd uit 20 sessies met lichamelijke activiteit onder begeleiding (24), die in een periode van 6 maanden plaatsvinden.

Tijdens de intake, documenteert de beweegcoach de doelen en de persoonlijke voorkeuren van de deelnemer m.b.t. lichamelijke activiteit. Op basis hiervan wordt vervolgens de instroom van de deelnemer in het programma geregeld.

*Bewegen onder begeleiding*

Na de intake stroomt de deelnemer in bij een cursus die aansluit bij zijn/haar voorkeuren. Bij aanvang van de cursus wordt een eigen bijdrage van 40 euro gevraagd. Na afloop van het programma ontvangt de deelnemer een gedeelte retour indien hij/zij tenminste aan 85% van de sessies heeft deelgenomen. Deelname wordt gemonitord door de beweegcoaches, die volgens protocol contact opnemen met een deelnemer indien hij/zij bij een sessie verstek laat gaan.

Deze werkwijze (met een eigen bijdrage) wordt beschouwd als een essentieel onderdeel van het succes van het programma. Eerdere evaluaties hebben laten zien dat door deze bijdrage de motivatie en toewijding van de deelnemer toeneemt.

De deelnemer kan bij BOR kiezen uit diverse activiteiten zoals Fitness, Aerobics and aquarobics, die zowel in gemengde als niet-gemengde groepen worden aangeboden (24).

Deze activiteiten sluiten goed aan bij de voorkeuren van de doelgroep (SUNSET, niet gepubliceerd).

Om de motivatie en deelname te vergroten wordt rekening gehouden met de belangrijkste punten die naar voren zijn gekomen uit de focusgroep discussies en de eerdere procesevaluatie van BOR (unpublished, 22). Bijvoorbeeld:

- De activiteiten worden aangeboden in gemengde en niet gemengde groepen (alleen vrouwen of alleen mannen)
- Flexibele roosters om de combinatie tussen sporten en school of sporten en werk te vereenvoudigen.
- Beschikbaarheid van goed bereikbare faciliteiten op loopafstand of eenvoudig bereikbaar per openbaar vervoer om de kosten (van vervoer) te beperken en tegemoet te komen aan eventuele gevoelens van onveiligheid, die met name in de avond een rol spelen.
- Lage eigen bijdrage, waarbij rekening gehouden wordt met beperkte financiële middelen van deelnemers.
- Erkenning van het belang van sociale steun. Eerder onderzoek laat zien dat deelnemers in BOR veel sociale steun ervaren. Daarnaast zal specifiek aandacht worden besteed aan steun van de partner en eventuele familieleden.
- De begeleiding zal zich daarnaast richten op empowerment van de deelnemer om hem/haar in staat te stellen om te gaan met negatieve reacties.
- Betrokkenheid van professionals bij het programma. Dit wordt bereikt doordat de formele verwijzing wordt ondertekend door de eigen huisarts en de betrokkenheid van speciaal opgeleide coaches en leefstijladviseurs.

De lichamelijke activiteit binnen het programma is er vooral op gericht om de cardiovasculaire fitheid te doen toenemen en in mindere mate de spierkracht.

De persoonlijke begeleiding binnen BOR bestaat uit 2 fases. De eerste fase bestaat uit 10 sessies die gericht zijn op het motiveren van de deelnemer door mensen de voordelen/positieve effecten van sporten te laten ervaren. De daaropvolgende 10 sessies zijn gericht de deelnemers op zodanige wijze te stimuleren (empowerment) dat de gedragsverandering kan worden volgehouden na afloop van het programma.

Gedurende het begeleidingstraject zal de deelnemer worden geadviseerd hoe hij/zij het doel van 30 minuten beweging gedurende tenminste 5 dagen per week kan bereiken. Dit wordt gedaan door het stimuleren van dagelijkse beweging (zoals vaker lopen of wandelen en dansen tijden sociale bijeenkomsten) en het stimuleren van deelname aan georganiseerde sportactiviteiten (zoals de BOR cursus of cursussen via lokale sportverenigingen).

Tevens wordt aandacht besteed aan ervaren sociale steun, de houding t.a.v. sporten en bewegen en manieren om om te gaan met (negatieve) feedback vanuit de omgeving. Deze begeleiding en coaching wordt uitgevoerd door speciaal getrainde coaches onder toezicht van de beweegcoach.

*Nabespreking en verdere verwijzing*

Na afloop van de 20 sessies, vindt een nagesprek plaats met de deelnemer.

Samen met de beweegcoach worden de bereikte doelen en ervaringen in het programma geëvalueerd. Tegelijk wordt de deelnemer doorverwezen naar het vervolgprogramma ‘Bewegen zonder recept’ (BZR) (24). BZR is opgezet om tegemoet te komen aan een punt dat van belang bleek onder Hindostaanse deelnemers in de eerdere proces evaluatie van BOR: deelnemers gaven aan moeite te hebben met het voortzetten van de positieve gedragsverandering na afloop van BOR. Binnen BZR wordt de transitie van bewegen onder begeleiding naar zelfstandig bewegen gefaciliteerd door middel van een motiverende sessie waarin de deelnemer wordt gestimuleerd het gedrag voort te zetten en geleerd de activiteiten actief te plannen. Het proces wordt verder ondersteund door reguliere uitnodigingen voor gratis deelname aan proeflessen bij lokale sportverenigingen, sportscholen en buurtcentra (bijvoorbeeld Lady Sport Fitness Centers and Zwemclub CWP). Tevens is er dan de mogelijkheid om, tegen gereduceerd tarief, een abonnement of lidmaatschap aan te gaan.

***Overige lichamelijke activiteit***

Indien de deelnemer niet wenst mee te doen met het reguliere BOR aanbod, zal in overleg met de beweegcoach worden gezocht naar een andere geschikte beweegcursus of activiteit.

### 3.2.2.b Voedingsinterventie

De voedingsinterventie zal worden uitgevoerd door getrainde diëtisten met kennis van de Hindostaanse cultuur. Het algemene doel is om een voedingspatroon te bereiken conform de ‘Richtlijnen goede voeding” (25). Uit ons eerdere onderzoek is gebleken dat dit voor de Hindostaanse populatie met name vertaald kan worden naar: 1) dagelijks ontbijten, 2) een regelmatig maaltijdpatroon, 3) meer fruit en groente and 4) verhoogde vezelinname (2,22, unpublished).

De begeleiding is gebaseerd op een combinatie van ‘motivational interviewing’ en het ‘Transtheoretical Model’, aangepast aan de Hindostaanse populatie (26-29). Deze combinatie van het ‘Transtheoretical model’ met ‘motivational interviewing’ wordt erkend als een effectieve methode om gedragsverandering te bewerkstelligen (27-28).

Voor aanvang van de interventie zal de diëtist getraind worden in het gebruik van deze technieken. Daarnaast is er, ter referentie, een protocol beschikbaar van de procedures binnen de interventie en gedetailleerde informatie over de relevante determinanten in de doelgroep.

*Individuele begeleiding*

Tijdens de eerste 6 maanden van de interventie zijn er 6-8 individuele consulten gepland met de diëtist. Deze consulten kunnen onderverdeeld worden in 2 fases.

Fase 1 (2-3 sessies):

Tijdens deze fase beoordeelt de diëtist de motivatie en fase van gedragsverandering van de deelnemer m.b.t. verandering van de voeding en bewustzijn van de associatie tussen voeding en het risico op diabetes (28). Indien de motivatie voor verandering laag is, zal de dietist pogen (volgens de methode motivational interviewing) de intrinsieke motivatie van de deelnemer te verhogen door de deelnemer discrepanties te laten onderzoeken en op te laten lossen (29).

Op basis van de focusgroep discussies die hebben plaatgevonden verwachten wij bijvoorbeeld dat deelnemers overtuigd zullen moeten raken van voordelen van het nuttigen van een ontbijt voor hun prediabetes, of dat een ontbijt uit verschillende soorten voedingsmiddelen kan bestaan (niet gepubliceerd).

Daarnaast worden de deelnemers gevraagd hun dieet te monitoren met als doel het bewustzijn van de discrepanties tussen de eigen voeding en de voedingsrichtlijnen te verhogen.

Fase 2 (4-6 sessies):

De tweede fase start zodra de deelnemer de ‘preparation stage’ heeft bereikt; d.w.z. dat de deelnemer besloten heeft zijn/haar gedrag te willen veranderen.

Op dat moment zal de deelnemer samen met de diëtist een persoonlijk plan samenstellen, waarbij rekening gehouden wordt met de persoonlijke omstandigheden. De diëtist zal de deelnemers aanmoedigen om doelen te stellen (bijv. Om de aspecten te veranderen die naar voren zijn gekomen uit de monitoring van de voeding (30-31)) en om de eigen effectiviteit te verhogen.

Gedurende fase 2 zullen de deelnemers werken aan hoe zij meer vertrouwen kunnen krijgen in het kunnen bereiden van fruit en hoe groenten op gezondere wijze bereid kunnen worden zonder verlies van smaak en het kunnen weerstaan van de druk van derden om traditioneel te koken. Sommige van deze aspecten/vaardigheden kunnen extra geoefend worden in de hieronder beschreven optionele sessies.

Alle tijdens de consulten besproken onderwerpen (motivationele factoren, barrières en deelname/steun van vrienden en familieleden) en persoonlijke doelen zullen worden gedocumenteerd.

*Optionele sessies: huisbezoek en groepssessies*

Voor iedere deelnemer worden 1 of 2 huisbezoeken georganiseerd met familieleden en/of andere leden van het huishouden. Het doel van deze bezoeken is het verminderen van de ervaren sociale druk en het verhogen van de sociale steun voor een gezondere voeding.

Dit zijn belangrijke barrières in de doelgroep, hetgeen bijvoorbeeld wordt geïllustreerd door het feit dat deelnemers aan de focusgroep discussies aangaven een hoge sociale druk te voelen om traditionele, minder gezonde maaltijden te bereiden (niet gepubliceerd).

Naast de huisbezoeken worden twee optionele sessies aangeboden die gericht zijn op onder andere het verhogen van de eigen effectiviteit en het aanleren van vaardigheden die de deelnemers in staat zullen stellen om traditionele maaltijden aan te passen aan de richtlijnen voor goede voeding. Een sessie zal bestaan uit een bezoek aan een lokale supermarkt en een sessie uit een kookles waarin deelnemers de gelegenheid krijgen om een gezondere versie van een traditionele maaltijd te bereiden en te proeven.

### 3.2.3 Vervolgcontacten

Na afloop van het de voedingsinterventie en het beweegprogramma (6 maanden), zullen de deelnemers eerst na 3 maanden en daarna elke 6 maanden worden benaderd voor een korte evaluatie van de leefstijl en verhoging van de motivatie. Tijdens al deze contacten is het doel om de deelnemers te helpen terugval te voorkomen of de deelnemers te helpen bij het opstellen van een plan, indien er terugval is opgetreden.

### 3.2.4 Training en intervisie diëtisten

Alle bij het project betrokken diëtisten zullen een training krijgen in motivational interviewing. Tevens zullen er gedurende het traject intervisie bijeenkomsten worden gehouden voor bespreking van lastige situaties en het verbeteren van de counselingvaardigheden.

## 3.3 Controle groep

In de controlegroep zal een leefstijlconsulent de algemene componenten van de intake van BOR en de diëtist volgen, zoals het in kaart brengen van de voedingsgewoonten en de voorkeuren t.a.v. lichamelijke activiteit. Op basis van deze informatie zal de leefstijlconsulent de deelnemers voorzien van een eenvoudig, persoonlijk leefstijladvies.

Het advies is persoonlijk in de zin dat, afhankelijk van de kenmerken van de deelnemer, het advies zich zal toespitsen op voeding, beweging of overgewicht.

Inhoudelijk is het advies echter van algemene aard, dat wil zeggen, conform de Nederlandse richtlijn voor gezond bewegen, de richtlijn gezonde voeding en de internationale richtlijn voor overgewicht bij Zuidoost-Aziatische populaties (BMI onder de 23 kg/m2).

Na 6 maanden en na 18 maanden is er wederom een afspraak met de leefstijlconsulent gepland. Daarnaast worden de respondenten uit de controlegroep in de gelegenheid gesteld om in bepaalde periodes zelf telefonisch advies te vragen bij de leefstijlconsulent voor advies. Deze momenten worden in de nieuwsbrieven die zij ontvangen aangekondigd.

# 4. Design

## 4.1 Onderzoeksdesign screening

De screening zal plaatsvinden in Den Haag bij Hindostaanse Surinamers. Potentiële deelnemers zullen geselecteerd worden uit registraties van huisartsenpraktijken in Den Haag. In totaal zullen er 6000 Hindostanen in de leeftijd van 18 t/m 60 jaar worden uitgenodigd om deel te nemen aan de screening.

Van de deelnemers die benaderd zijn zullen redenen voor het niet willen deelnemen aan de studie worden genoteerd.

*Eerste screening*

De dataverzameling tijdens de eerste screening bestaat uit een korte vragenlijst en een lichamelijk onderzoek. De dataverzameling zal plaatsvinden op een locatie van de Stichting Huisartsenlaboratorium in Den Haag. De deelnemers zullen worden gevraagd om nuchter te zijn op het tijdstip van de screening. Na ontvangst zullen deelnemers gevraagd worden het informed consent te ondertekenen. Vervolgens wordt met een vingerprik de nuchtere glucosewaarde bepaald. Wanneer het nuchter bloedglucosegehalte <8.5 mmol/l dan wordt er een orale glucose tolerantie test (OGTT) uitgevoerd. Hiervoor wordt er nuchter een veneuze bloedsample afgenomen. Dan worden respondenten gevraagd om 75 g glucose opgelost in water op te drinken. Vervolgens wordt 2 uur na inname van de glucosedrank een tweede veneuze bloedsample afgenomen. In de wachttijd worden respondenten gevraagd een korte vragenlijst in te vullen betreffende risicofactoren voor diabetes, zoals leeftijd, geslacht, etniciteit, voorkomen van diabetes in de familie. Tevens vindt er een aanvullend lichamelijk onderzoek plaats. Hier zullen antropometrische gegevens bepaald worden en zal de bloeddruk gemeten worden. In het laboratorium zullen het nuchter plasma glucosegehalte, HbA1C, insuline en het en 2-uurs glucosegehalte bepaald worden.

Resultaten van de screening zullen worden terug gerapporteerd naar de respondent en naar de huisarts van de respondent. De terugrapportage naar de huisarts zal ook adviezen bevatten voor eventuele verdere actie. De huisarts zal worden gevraagd contact op te nemen met respondenten bij wie tijdens de eerste screening diabetes gediagnosticeerd is. Hierbij geldt het advies om verdere diagnostiek en behandeling in te stellen, conform de geldende NHG standaard.

*Tweede screening*

Personen die tijdens de eerste screening een verhoogd nuchter en/of 2-uurs glucose gehalte hadden, zullen worden uitgenodigd voor een tweede screening. Deze tweede screening zal plaatsvinden op dezelfde locatie als de eerste screening. De deelnemers zullen worden gevraagd om nuchter te zijn op het tijdstip van de screening. De dataverzameling bestaat uit een meer uitgebreide vragenlijst, een lichamelijk onderzoek en een nieuwe orale glucose tolerantie test (OGTT). Tevens wordt de deelnemer gevraagd een urine monster mee te nemen.

De vragenlijst gaat over (determinanten van) bewegen, voedingsgewoonten, en over risicofactoren zoals roken en alcoholconsumptie. Tijdens het lichamelijk onderzoek zullen antropometrische gegevens worden bepaald en zal er een extra nuchter bloed diabetesmonster worden afgenomen. Tevens wordt een cardiovasculaire fitheids test afgenomen (de Chester step test) en vindt een meting van het lichaamsvet (bio-impedantie meting) plaats.

De metingen zullen worden uitgevoerd door een getrainde onderzoeksassistent.

In het laboratorium zal naast het nuchter plasma glucosegehalte, HbA1C, insuline en 2-uurs glucose gehalte, ook het lipiden profiel gemeten worden. Uit het urine monster wordt het albumine versus creatinine gehalte berekend.

Voor deelnemers met prediabetes, vastgesteld op basis van de gemiddelde glucose waardes uit de eerste en tweede screening, die daarna doorstromen naar de interventiestudie, vormen de verzamelde gegevens uit de tweede screening tevens de baseline meting voor de trial.

*Herscreening*

Personen met normale glucose zullen na 3 jaar worden uitgenodigd voor een herhaalde screening. Hiervoor wordt tijdens de eerste screening toestemming gevraagd. De inhoud van deze screening zal overeen komen met de tweede screening.

## 4.2 Onderzoeksdesign leefstijlinterventie

Om de effectiviteit van de leefstijlinterventie te onderzoeken zal een gerandomiseerde trial (enkelblind) worden uitgevoerd. Alle personen met prediabetes die hebben aangegeven deel te willen nemen aan de leefstijlinterventie studie zullen, na formele toestemming (informed consent), gerandomiseerd worden naar de controlegroep of de interventiegroep. De personen in de interventiegroep krijgen een leefstijlinterventie. De inhoud van deze interventie staat beschreven in hoofdstuk 3. De controlegroep krijgt een eenvoudige leefstijlbegeleiding, zoals eveneens is omschreven in hoofdstuk 3.

*Baseline*

De gegevens uit de tweede screening zullen worden gebruikt als baseline voor de trial.

*Nametingen*

Na 12 maanden en na 36 maanden zal zowel in de interventiegroep als de controlegroep een nameting plaatsvinden. De opzet van deze meting is gelijk aan de opzet van de meting tijdens de tweede screening: een vragenlijst, orale glucose tolerantie test, urineonderzoek, en een lichamelijk onderzoek bestaande uit meting van de antropometrie, bloeddruk, cardiovasculaire fitheid en hoeveelheid lichaamsvet.

De tweede nameting na 36 maanden valt samen met de herscreening voor personen bij wie normoglycemie geconstateerd is tijdens de eerste screening.

*Uitval*

Indien deelnemers aan de trial hun deelname wensen te beëindigen, zullen de redenen voor uitval worden gedocumenteerd.

## 4.3 Onderzoekspopulatie

### 4.3.1 Onderzoekspopulatie screening

De screening zal plaatsvinden in de regio Den Haag. In totaal zullen 6000 Hindostaanse Surinaamse mannen en vrouwen uitgenodigd worden voor de screening. De personen moeten tussen de 18 en 60 jaar zijn en hebben nog geen gediagnosticeerde diabetes. Potentiële deelnemers zullen geselecteerd worden uit registraties van huisartsenpraktijken in Den Haag. Wanneer er in de huisartsenregistratie geen informatie beschikbaar is over de etniciteit van de patiënten dan zullen, evenals tijdens de haalbaarheidsstudie, patiënten van Hindostaanse afkomst geïdentificeerd worden door middel van naam analyses. Voor deze procedure is een lijst beschikbaar met veelvoorkomende Hindostaanse namen.

Om afhankelijkheid tussen deelnemers zoveel mogelijk te voorkomen, zal slechts 1 persoon per huishouden (op basis van het geregistreerde huisadres) worden benaderd. Vrouwen die zwanger zijn zullen gevraagd worden of zij na de zwangerschap nogmaals benaderd willen worden.

### 4.3.2 Onderzoekspopulatie leefstijlinterventie

De personen waarbij tijdens de screening op basis van de gemiddelde waarde van de eerste en tweede OGTT prediabetes (IFG of IGT) is vastgesteld worden benaderd om deel te nemen aan de leefstijlinterventie trial. Het gebruik van de gemiddelde waarde is een wetenschappelijk geaccepteerde methode en wordt bijvoorbeeld ook gebruikt binnen de Nederlandse HOORN studie.

De definitie van prediabetes (IFG en IGT) staan beschreven in tabel 1. Door de screening ontdekte diabetes patiënten en proefpersonen met een chronische aandoening of medicatiegebruik die samenhangt met glucose-intolerantie worden niet benaderd voor de interventiestudie. Personen die door psychische of lichamelijke aandoening worden belemmerd in hun lichamelijke activiteit worden eveneens uitgesloten voor het onderzoek. Dit geldt tevens voor proefpersonen die al deelnemen aan andere leefstijlprogramma’s of intensieve leefstijlcounselling.

*Tabel 1. Criteria voor de diagnose van (pre)diabetes )*

| Normoglycemia | Prediabetes  IFG of IGT | Diabetes |
| --- | --- | --- |
| **FPG < 5.6 mmol/l**  **2-h PG < 7.8 mmol/l** | **FPG ≥ 5.6 - < 7.0 mmol/l (IFG)**  **2-h PG ≥ 7.8 and < 11.1 mmol/l (IGT)** | **FPG ≥ 7.0 mmol/l**  **2-h PG ≥ 11.1 mmol/l** |

From: Diagnosis and classification of diabetes mellitus. Diabetes Care 2007;30 Suppl 1:S42-S47.

FPG = Fasting Plasma Glucose

2-h PG = 2-hour post-load glucose (glucosewaarde 2 uur na inname van 75 gram glucose opgelost in water)

IFG = Impaired Fasting Glucose

IGT = Impaired Glucose Tolerance

## 4.4 Trajectdoorloop patiënten in de studie

6000 Hindostanen geselecteerd uit huisartsen registraties

Eerste screening, eventueel gevolgd door 2e screening (bij verhoogd bloedglucose)

Patiënten met normoglycemie

Patiënten met prediabetes

Patiënten met diabetes

Terugrapportage naar huisarts en deelnemer. Oproep voor herscreening na 3 jaar.

Terugrapportage naar huisarts en deelnemer. Verdere begeleiding vindt plaats via de huisarts

Interventiegroep (leefstijlinterventie programma voor 3 jaar).

Controle groep

(algemene informatie over gewichtsreductie, lichamelijke activiteit en gezonde voeding.

*Figuur 2: Diagram studiedoorloop*

### 4.4.1 Natraject nieuw gedetecteerde diabeten

Van sommige respondenten zal tijdens het onderzoek blijken dat ze diabetes, een verhoogde bloeddruk of een ongunstige verdeling van het lipidenprofiel (tijdens tweede screening of nametingen) hebben zonder dat ze hier zelf van op de hoogte waren. Deze personen krijgen het advies om contact op te nemen met de huisarts zodat de metingen herhaald kunnen worden en verder beleid kan worden ingesteld conform de geldende NHG standaarden. Dit zal voorafgaand aan het onderzoek ook met de huisarts worden besproken.

## 4.5 Statistische power

Het doel van deze studie is om de effectiviteit van het preventieprogramma ten aanzien van het voorkomen van incidente diabetes aan te tonen. Er is een powerberekening uitgevoerd om te kunnen bepalen wat de omvang van de onderzoekspopulatie moet zijn om relevante verschillen in de incidentie van diabetes na 3 jaar met statistische significantie aan te kunnen tonen. Op basis van deze berekening moeten 6000 personen worden uitgenodigd voor de studie om ca. 500 personen met prediabetes te kunnen insluiten in de trial (250 per arm). Met deze aantallen kan een 50% risicoreductie door de interventie worden aangetoond (de reductie was 28-58% in eerdere studies, afhankelijk van de intensiteit van de interventie (32-35)).

Bij de berekeningen is verder uitgegaan van de volgende aannames:

- een power van 0.80 en een significantie van 0.05
- 85% ‘eligible’ deelnemers, met een respons van 43% voor de eerste screening (haalbaarheidsstudie, niet gepubliceerd)
- een prevalentie van prediabetes van 40% (haalbaarheidsstudie, niet gepubliceerd)
- een participatie van 60% in de trial
- een cumulatieve incidentie van diabetes bij personen met prediabetes van 25% in 3 jaar (20-55% (32-35))
- drop out rate van 20%.

Verder moeten ca. 20% met normoglycemie bij baseline na 3 opnieuw gescreend worden om een incidentie van diabetes van 5% (betrouwbaarheidsinterval 3-8%) aan te kunnen tonen (36).

# **5. Dataverzameling screening en effectiviteitsonderzoek**

## 5.1 Eerste screening

De dataverzameling tijdens de screening omvat een vragenlijst, een lichamelijk onderzoek en een orale glucose tolerantie test (OGTT). In verband met bloedafname zullen respondenten gevraagd worden om nuchter te zijn (zie ook Figuur 3).

### 5.1.1 Vragenlijst

*Eerste screening*

Bij de eerste screening zal deelnemers gevraagd worden om een korte vragenlijst in te vullen betreffende determinanten van diabetes. De vragenlijsten die gebruikt zullen worden zijn in deze paragraaf beschreven. De meeste vragen zijn ook gebruikt in het SUNSET onderzoek.

*- Sociaal demografische gegevens*

Voor de meting van leeftijd, geslacht en burgerlijke staat/samenlevingsvorm zijn de vragen overgenomen uit het SUNSET onderzoek.

*- Etnische herkomst*

Om onderscheid te maken in etnische groepen binnen de Surinaamse populatie wordt aangeraden om gebruik te maken van zelfidentificatie (37). Eerst zal het geboorteland van de respondent en van zijn vader en moeder worden nagevraagd. Vervolgens zal worden nagevraagd uit welke bevolkingsgroep de vader/moeder oorspronkelijk afkomstig is.

*- Sociaal economische status*

Standaardindicatoren voor de meting van sociaaleconomische status in de autochtone bevolking zijn inkomens-, opleidings- en beroepsniveau (advies Programmacommissie Sociaal Economische gezondheidsverschillen, 1989). Naar verwachting zijn dit ook in de Hindostaans-Surinaamse populatie valide indicatoren ter meting van sociaaleconomische status. De opleidingsvraag zal moeten aangepast worden aan de Surinaamse situatie. Dit is overgenomen uit het eerder genoemde ISEO (o.a. Martens,1999) onderzoek.

*- Gezondheidstoestand en zorggebruik*

Zowel het voorkomen van diabetes, hypertensie en hart- en vaatziekten zal worden nagevraagd aan de hand van een vragenlijst. Tevens zal worden gevraagd naar recent zorggebruik in verband met deze gezondheidsklachten.

*- Familie historie*

Deze zal worden nagevraagd aan de hand van een vragenlijst, welke het voorkomen van diabetes meet bij eerste en tweedegraads familieleden voor de leeftijd van 60 jaar. Daarbij zal genoteerd worden om welke familieleden het gaat (kant van vader, moeder etc.). Deze vragen zijn overgenomen uit het SUNSET onderzoek.

*- Kosten*

Er zal nagevraagd worden of de deelnemers vrij hebben genomen van hun werk in verband met de screening. Tevens zal worden nagegaan of er kosten, zoals bijvoorbeeld reiskosten, zijn gemaakt in samenhang met de screening.

*- Mening over screening*

Aan de deelnemers zal worden gevraagd wat hun mening is over de screening.

### 5.1.2 Lichamelijk onderzoek

Tijdens het lichamelijk onderzoek zullen de volgende metingen worden verricht:

| Lengte | Met behulp van een meetlint zal de lengte worden bepaald. De lengte is het gemiddelde van 2 metingen. |
| --- | --- |
| Gewicht | Het gewicht wordt bepaald met een digitale weegschaal. Personen worden gewogen zonder schoenen en jas. |
| Middelomtrek | De middelomtrek wordt gemeten met een meetlint op het smalste deel van het middel tussen de onderste rib en de bovenkant van het heupbeen direct op de huid. De middelomtrek is het gemiddelde van 2 metingen. |
| Bloeddruk | De bloeddruk wordt gemeten met een sphygmanometer. De bloeddruk is het gemiddelde van 2 metingen. |
| Nuchter en 2 uurs bloedglucose gehalte, insuline en HbA1c | Door middel van een venapunctie in de arm zal er in het totaal maximaal 50 ml bloed worden afgenomen (35 ml voor en 7 ml na afloop van de OGTT). Hieruit zal ondermeer het glucosegehalte bepaald worden. |

### 5.1.3 OGTT

De OGTT bestaat uit drie onderdelen:

1. Nuchtere bloedafname

- Vingerprik: Bij alle respondenten wordt het nuchter bloedglucose gehalte bepaald met behulp van een vingerprik. Respondenten met een nuchter bloedglucose >8.5 mmol/l worden uitgesloten voor de OGTT. Dit geldt tevens voor respondenten die reeds behandeld worden voor diabetes met insuline of tabletten.

- Veneuze bloedafname

1. Drinken glucosedrank

De respondenten met een nuchter bloedglucose <8.5 (vingerprik) worden gevraagd een glucosedrank op te drinken. Deze bestaat uit 75 g glucose opgelost in 250 ml water.

1. Bepalen bloedglucose na 2 uur

Na 2 uur wordt opnieuw het bloedglucose gehalte bepaald met behulp een tweede veneuze bloedafname.


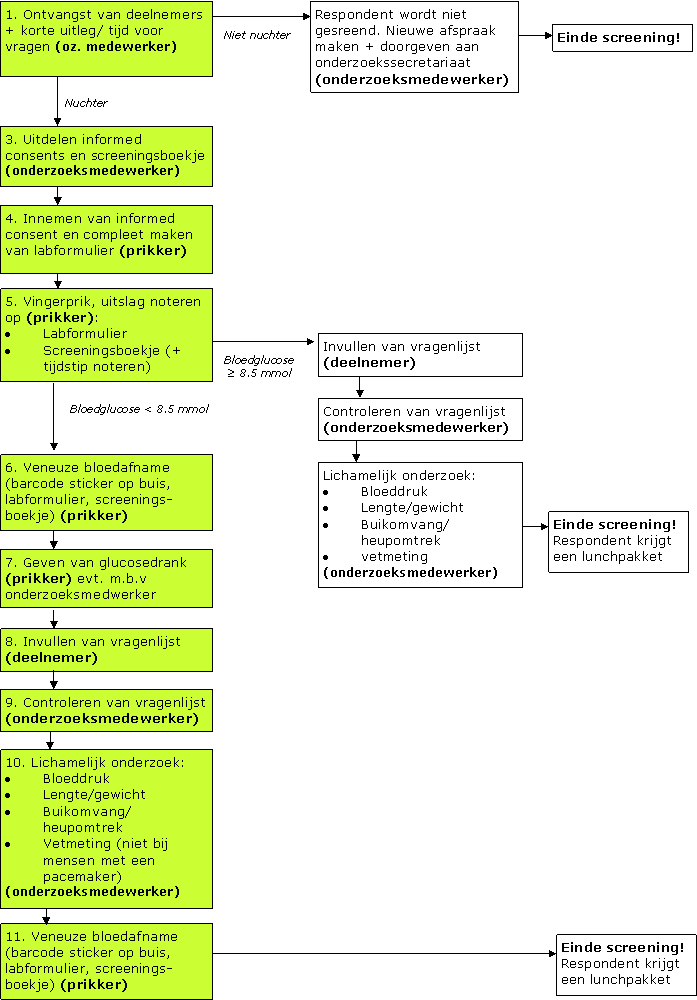


*Figuur 3. Opzet van de eerste screening*

## 5.2 Tweede screening, nameting na 12 maanden en 36 maanden

De tweede screening, tegelijk baseline meting van de leefstijlinterventie, bestaat uit een OGTT, lichamelijk onderzoek (inclusief bloedafname) en een aanvullende vragenlijst. In verband met bloedafname zullen respondenten gevraagd worden om nuchter te zijn. Tevens wordt aan de respondenten gevraagd om een potje ochtendurine mee te nemen. Het lichamelijk onderzoek en de OGTT staan beschreven in hoofdstuk 5.1. In dit hoofdstuk zullen de laboratorium bepalingen, het aanvullend lichamelijk onderzoek en de vragenlijsten worden beschreven.

### 5.2.1 Bepalingen in het lab

De volgende labbepalingen zullen worden gedaan.

| Monster | Bepaling |
| --- | --- |
| Nuchter bloedafname | - Nuchter bloedglucose gehalte - Insuline - HbA1C - Creatinine - Lipidenprofiel (LDL en HDL cholesterol, triglyceriden) - CRP |
| Urine | - Albumine en creatinine gehalte |

### 5.2.2 Vragenlijst

Bij de tweede screening en nametingen van de interventie zullen deelnemers gevraagd worden om een korte vragenlijst in te vullen betreffende risicofactoren van diabetes. De vragenlijsten die gebruikt zullen worden zijn in deze paragraaf beschreven. De meeste vragen zijn ook gebruikt in het SUNSET onderzoek.

*- Roken*

Voor de meting van rookgedrag zal gebruik worden gemaakt van een gevalideerde vragenlijst die onder andere in onderzoek van stichting volksgezondheid (stivoro) en het SUNSET onderzoek zijn gebruikt.

*- Voedingsgewoonten*

Er zullen een aantal belangrijke aspecten in de voeding worden gemeten zoals bijvoorbeeld: vetconsumptie, groente- en fruitconsumptie en ontbijtgedrag. Deze worden gemeten met behulp van een voedselfrequentie lijst.

*- Lichaamsbeweging*

De mate van lichaamsbeweging in werk, vrije tijd en sport zal worden bepaald aan de hand van voor autochtone Nederlanders gevalideerde vragenlijst (SQUASH). Op basis van de haalbaarheidsstudie is nagegaan dat de activiteiten die in deze lijst genoemd worden ook gelden voor de Hindostaanse populatie.

*- Gezondheidstoestand (alleen bij 12 en 36 maanden) en zorggebruik*

Zowel het voorkomen van diabetes, hypertensie en hart- en vaatziekten zal worden nagevraagd aan de hand van een vragenlijst. Tevens zal worden gevraagd naar recent zorggebruik in verband met deze gezondheidsklachten.

*- Kwaliteit van leven en stress*

Zowel de lichamelijke, als het psychische en sociale domeinen van kwaliteit van leven worden nagevraagd; lichamelijk functioneren, psychische klachten en de mate waarin afbreuk wordt gedaan aan de mogelijkheden om sociale rollen te vervullen.

*- Kosten*

De kosten voor deelname aan de interventie en voor deelname aan de metingen zullen in kaart gebracht worden, met name de directe kosten zoals transportkosten, (para-)medische begeleiding en kosten voor deelname aan de interventie (o.a. aanschaf sportkleding). Daarnaast zal gevraagd worden naar de geïnvesteerde tijd om de directe niet-medische kosten te kunnen bepalen, zoals (e.g. tijd voor deelname interventie) en indirecte kosten (o.a. gerelateerd werkverzuim).

*- Procesmaten*

Hierbij zullen ondermeer de volgende punten in kaart gebracht worden: therapietrouw (gemiste bezoeken, gestelde en behaalde doelen), factoren die de therapietrouw beïnvloeden, tevredenheid met de interventie en de mate waarin die aansluit bij de deelnemers.

# 6. Analyse screening en effectonderzoek

## 6.1 Screening

De deelname aan de screening (respons op de uitnodiging en daadwerkelijke deelname aan screening), de prevalentie van diabetes en prediabetes tijdens de screening zullen worden beschreven.

Daarnaast zal de 3-jaars incidentie van prediabetes en diabetes onder personen met een normale bloedglucose tijdens de eerste screening berekend worden om een indruk te verkrijgen van het gewenste screeningsinterval.

Alle analyses zullen gestratificeerd worden naar leeftijd, geslacht en andere relevante determinanten. Er zal ook gekeken worden naar eventuele verschillen tussen huisartsenpraktijken.

Door middel van logistische regressie analyse zal een combinatie van (biomedische) parameters worden gezocht die voorspellend zijn voor het hebben van pre-diabetes of diabetes zoals geïdentificeerd m.b.v. de OGTT. De diagnostische waarde van deze combinatie zal worden geëvalueerd aan de hand van conventionele maten zoals de sensitiviteit, specificiteit, positief predictieve waarde en oppervlakte onder de ROC curve).

## 6.2 Interventie

De deelname aan de screening zal worden berekend.

Voorts zal de incidentie van diabetes (de primaire uitkomst) en het verschil in incidentie tussen de interventie- en controlegroep na 3 jaar worden berekend, zowel met behulp van reguliere methodes (e.g. Kaplan-Meier curves, Cox proportional hazards) als met multilevel analyses om groepseffecten te kunnen onderscheiden van individuele effecten. Tevens zal het effect van de interventie op de secundaire en intermediaire maten geëvalueerd worden, evenals de neveneffecten van de screening (o.a. het verschil in de kwaliteit van leven).

Al deze analyses zullen uitgevoerd worden volgens het intention-to-treat principe. Waar nodig, zal er gecorrigeerd worden voor verschillen in achtergrondvariabelen.

Tot slot, zal binnen de interventiegroep een multivariate regressie worden uitgevoerd om de invloed van de baseline variabelen en de mate van deelname aan de interventie (o.a. de therapietrouw en welke thema’s behandeld zijn in de interventie) op de incidentie van diabetes en de veranderingen in de secundaire en intermediaire uitkomsten te bestuderen.

## 6.3 Programma evaluatie

### 6.3.1 Kosten

Het primaire eindpunt van de economische evaluatie is een (voorkómen) case van diabetes. De analyse wordt opgezet als een zogenaamde cost-benefit analyse, waarin de additionele kosten per voorkómen geval van diabetes worden berekend (38).

Verschillen in de kosten zullen worden berekend volgens het maatschappelijk perspectief. Binnen dit perspectief wordt de waarde van alle investeringen in het programma meegewogen, zowel de daadwerkelijke kosten voor ontwikkeling en implementatie van het programma als de aan het programma gerelateerde kosten.

Kosten van het programma (mankracht, materialen etc.) zullen geregistreerd worden en aangevuld met gegevens uit de vragenlijsten. Gegevens over de tijdsinvestering van betrokken huisartsen en overige professionals zullen worden verzameld met behulp van een interview.

Gegevens over de frequentie van deelname, prevalentie en incidentie van diabetes kunnen geschat worden op basis van de tijdens de studie verzamelde data. Gegevens over de test frequentie en diagnoses bij afwezigheid van een preventieprogramma (‘usual care’) zullen worden geschat op basis van data over het zorggebruik in het jaar voorafgaand aan de screening uit de huisartsenregistraties.

Daarnaast zullen de door professionals geïnvesteerde uren worden meegewogen, evenals de overige uit registers verzamelde informatie. Zo zullen – om inzicht te krijgen in het zorggebruik- gegevens over huisartsbezoeken voor en na aanvang van het programma verzameld worden uit de huisartsenregistraties.

Zoals aangegeven, zullen op basis van bovenstaande processen de directe kosten, de directe niet-medische kosten en de indirecte kosten worden berekend. Waar mogelijk zullen kosten berekend worden aan de hand van geldende Nederlandse richtlijnen en tarieventabellen.

Additionele kosten ten gevolge van comorbiditeit zullen niet worden meegenomen.

Bij de berekeningen zal, waar van toepassing, tegen 3% worden verdisconteerd (46). Om rekening te houden met onbekende factoren en extrapolatie naar eventuele implementatie te vergemakkelijken, zullen univariate en zogenaamde probabilistische sensitiviteitsanalyses worden uitgevoerd.

**6.3.2. Processen, organisatie en ervaringen**

*Procesevaluatie*

Aan de huisarts en de praktijkondersteuner (onderzoeksassistenten) zal gedurende de screening 2 maal een kort telefonisch interview over de ervaringen in de screening worden afgenomen. Dit interview is bedoeld om inzicht te verkrijgen in de tijd die geïnvesteerd is in het geven van algemene informatie over diabetes, het beantwoorden van vragen over de screening en het testen en management van nieuw gediagnosticeerde diabeten. Tevens zal hun mening worden gevraagd over het proces, mogelijke belemmerende factoren voor het niet deelnemen aan de screening, beperkingen in het programma en er is ruimte voor verdere suggesties en feedback.

Ten aanzien van de processen en organisatie van het programma, zullen de volgende gegevens worden geanalyseerd?:

- De bereidheid tot deelname van huisartsen zal worden bepaald aan de hand van het aantal deelnemende artsen t.o.v. het aantal benaderde artsen en de redenen voor de eventuele weigering tot deelname.
- De aantallen geïdentificeerde deelnemers, de uitval door exclusie criteria, de respons en deelname en de doorstroming naar de interventie (inclusief de redenen voor niet deelnemen en aandacht voor eventuele selectieve deelname).
- Is het programma uitgevoerd zoals bedoeld? Wat was de uitval en therapietrouw tijdens de interventie? Gegevens over de therapietrouw (gemiste bezoeken, gestelde en behaalde doelen) zullen worden betrokken uit de registraties van de beweegcoaches, leefstijlconsulenten en diëtisten. Dit zal aangevuld worden met de gegevens uit de vragenlijsten.
- de organisatorische vereisten van het programma op basis van de eisen die worden gesteld aan de logistiek, organisatie en management van het programma.

De ervaringen van deelnemers (via de vragenlijsten) en professionals (op basis van de interviews) in de screening en interventie zullen in kaart gebracht worden door een analyse te maken van de perceptie van en tevredenheid met het programma, de persoonlijke investering in het programma (voor professionals bijvoorbeeld de tijdsinvestering, administratieve ballast, interacties met deelnemers), de perceptie van potentiële barrières, belemmerende en bevorderende factoren voor het deelnemen aan de interventie, factoren die de therapietrouw beïnvloeden, tevredenheid met de interventie en de mate waarin die aansluit bij de deelnemers. De leden van het interventieteam zullen geïnterviewd worden over hun rol in de interventie, de uitvoerbaarheid, voorspellingen en verwachtingen, mogelijke belemmeringen etc. Enkele vragen zullen ook in de vragenlijst worden opgenomen.

# Referenties

1. Baan, C.A., Poos, M.J.J.C. (2005). Hoe vaak komt diabetes mellitus voor en hoeveel mensen sterven eraan? In: Volksgezondheid Toekomst Verkenning, Nationaal Kompas Volksgezondheid.Bilthoven: RIVM. [http://www.nationaalkompas.nl](http://www.nationaalkompas.nl/).

2. Bindraban, N.R. et al. (2008). **Prevalence of diabetes mellitus and the performance of a risk score among Hindustani Surinamese, African Surinamese and ethnic Dutch: a cross-sectional population-based study.** BMC public health. 8():271.

3. Balarajan R. Ethnicity and variations in mortality from coronary heart disease. Health Trends 1996;28:45-51

4. Bos V et al. Ethnic inequalities in age- and cause-specific mortality in The Netherlands. Int.J.Epidemiol. 2004;33:1112-9.

5. Health Council of the Netherlands. Screening for type 2 diabetes. 2004. Ref Type: Report

6. UKPDS Group. Effect of intensive blood-glucose control with metformin on complications in overweight patients with type 2 diabetes (UKPDS 34). Lancet 1998;352:854-65.

7. Vegt de F et al. Relation of impaired fasting and postload glucose with incident type 2 diabetes in a Dutch population: The Hoorn Study. JAMA 2001;285:2109-13

8. (No Authors listed). The Diabetes Prevention Program. Design and methods for a clinical trial in the prevention of type 2 diabetes. Diabetes Care 1999;22:623-34.

9. Lindstrom J et al. Prevention of diabetes mellitus in subjects with impaired glucose tolerance in the Finnish Diabetes Prevention Study: results from a randomized clinical trial. J.Am.Soc.Nephrol. 2003;14:S108-S113.

10. Knowler WC et al. Reduction in the incidence of type 2 diabetes with lifestyle intervention or metformin. N.Engl.J.Med. 2002;346:393-403.

11. Tuomilehto J et al. Prevention of type 2 diabetes mellitus by changes in lifestyle among subjects with impaired glucose tolerance. N.Engl.J.Med. 2001;344:1343-50.

12. Mensink M et al. Study on Lifestyle Intervention and Impaired Glucose Tolerance Maastricht (SLIM): preliminary results after one year. International Journal of Obesity 2003;27:377-84.

13. Kreuter MW et al. Cultural tailoring for mammography and fruit and vegetable intake among low-income African-American women in urban public health centers. Prev.Med. 2005;41:53-62.

14. Mensink M et al. Study on lifestyle-intervention and impaired glucose tolerance Maastricht (SLIM): design and screening results. Diabetes Research and Clinical Practice 2003;60:49-58.

15. Steering Committee of the Western Pacific Region of the World Health Organization. The Asia-Pacific-Perspective: Redefining Obesity and its Treatment. 2000. Ref Type: Report

16. Gezondheidsraad. Richtlijnen Goede Voeding 2006. 2006.
Ref Type: Report

17. Kemper HGC, Ooijendijk WTM, Stiggelbout M. Consensus over de Nederlandse Norm voor Gezond Bewegen. Tijdschrift voor Sociale Gezondheidszorg 78, 180-183. 2000. Ref Type: Magazine Article

18. Nicolaou M, van Dam RM, Stronks K. Acculturation and education level in relation to quality of the diet: a study of Surinamese South Asian and Afro-Caribbean residents of the Netherlands. Journal of Hum.Nutr.Diet. 2006;19:383-93.

19. van Dam RM, Nicolaou M, Stronks K. Study of the diet of the Surinamese Amsterdammers. Nederlands tijdschrift voor Dietisten 60, 98-102. 2005. Ref Type: Magazine Article

20. Hagen M.A.M. van. Projectplan BOR fase 3 en 4: Werken aan verankering. Den Haag: STIOM,

2005.

21. Morgan O. Approaches to increase physical activity: reviewing the evidence for exercise-referral

schemes. Public Health 2005 May;119(5):361-70.

in Den Haag. Amsterdam, 2006.

22. Schmidt M et al. Wat beweegt de deelnemers? Een evaluatie van het project ‘Bewegen Op Recept’

23. Koelen MA et al. Health Education and Health Promotion. Wageningen Academic Publishers, 2004.

24. BOR protocol. STIOM, Den Haag, 2004.

25. Gezondheidsraad. Richtlijnen goede voeding 2006. A06/08. 2006. Den Haag, Gezondheidsraad. Ref

Type: Report

26. Kreuter et al. Achieving cultural appropriateness in health promotion programs: Targeted and

tailored approaches. Health Education & Behavior 30(2), 133-146.

27. Prochaska et al. Stages and Processes of Self-Change of Smoking: Toward an Integrative Model of

Change. Jounral of Consulting and Clinical Psychology 51 390-395.

28. DiClemente et al. Motivational Interviewing and the Stages of Change. In Miller W.R., & Rollnick S.

(Eds.), Motivational Interviewing. Preparing People for Change (pp. 201-216). New York: The Guilford

Press.

29. Miller W.R., & Rollnick S. (2002). Motivational Interviewing. Preparing people for change. New York:

The Guilford Press.

30. Boutelle KN et al. Further support for consistent self-monitoring as a vital component of successful

weight control. Obes Res 1998 May;6(3):219-24.

31. de Vries et al. Motives of Belgian adolescents for using sunscreen: The role of action plans. Cancer

Epidemiology Biomarkers & Prevention 15(7), 1360-1366.

32. Lindstrom J et al. Prevention of diabetes mellitus in subjects with impaired glucose tolerance in the

Finnish diabetes prevention study: results from a randomized clinical trial. J Am Soc Nephrol

2003;14:S108-13.

33. Knowler WC et al. Reduction in the incidence of type 2 diabetes with lifestyle intervention or

metformin. N Engl J Med 2002;346:393-403.

34. Mensink M et al. Study on Lifestyle Intervention and Impaired Glucose Tolerance Maastricht (SLIM):

preliminary results after one year. Int J Obes Relat Metab Disord 2003;27:377-84.

35. A. Ramachandran et al.The Indian Diabetes Prevention Programme shows that lifestyle modification

and metformin prevent type 2 diabetes in Asian Indian subjects with impaired glucose tolerance

(IDPP-1). Diabetologia. 2006 Feb;49(2):289-97.

36. Davey Smith G et al. Multiple Risk Factor Intervention Trial Research Group. Incidence of type 2

diabetes in the randomized multiple risk factor intervention trial. Ann Intern Med 2005;142:313-22.

37.Stronks K, Glasgow IK, Klazinga N. The identification of ethnic groups in health research, additional to the country of birth classification. Academic Medical Center, University of Amsterdam, Department of Social Medicine. 2004. Ref Type: Report

38. Marthe R. Gold et al. Cost-Effectiveness in Health and Medicine. Oxford Univ Press 1996..
