# Supplementary material for: Intensive Lifestyle Intervention in General Practice to Prevent Type 2 Diabetes among 18 to 60-Year-Old South Asians: 1-Year Effects on the Weight Status and Metabolic Profile of Participants in a Randomized Controlled Trial
Source: PLoS One. 2013 Jul 22;8(7):e68605. doi: 10.1371/journal.pone.0068605 (PMC3718785; doi:10.1371/journal.pone.0068605)
Supplement: Protocol S4 — Copy trial protocol approval by ethics committee before changes to the protocol part 1. (PDF) [file pone.0068605.s005.pdf]

Academisch Medisch Centrum
Universiteit van Amsterdam

Mw.prof.dr. K. Stronks
Sociale geneeskunde
J2-216

gecorrigeerde versie

Amsterdam, 15 april 2009
uw kenmerk:
ons kenmerk: MEC 08/371 # 09.17.0577
betreft:

Medisch Ethische Commissie
E2-236
doorkiesnummer: 566 7389/566 5880
fax: 5669015

Positief oordeel m.b.t. het project MEC 08/371:
De effectiviteit van een aangepast diabetes preventie programma voor Hindostaanse Surinamers, bestaande uit een screening gevolgd door een leefstijl interventie.

Geachte mevrouw Stronks,

Naar aanleiding van uw reactie d.d. 30 maart 2009 op onze brief van 18 maart jl. is bovengenoemd project, ons ter beoordeling voorgelegd op 22 december 2008, in onze vergadering van 9 april jl. nogmaals besproken.
Wij delen u gaarne mee dat onze commissie
- tot oordelen bevoegd krachtens artikel 2, tweede lid, onder a, van de Wet medisch-wetenschappelijk onderzoek met mensen (WMO);
- werkzaam volgens de ICH-GCP richtlijnen;
- op grond van de haar voorgelegde stukken als hierna vermeld en van de met uw brief van 30 maart 2009 verstrekte aanvullende informatie;
- gelet op artikel 3 van de WMO;
- vastgesteld hebbend dat aan de beoogde proefpersonen op adequate wijze informatie wordt gegeven over het uit te voeren onderzoek,

heeft besloten tot een positief nader oordeel over deze studie en de uitvoering daarvan door het AMC en door de Stichting Huisartsen Laboratorium.

Voorts hebben wij, overwegend dat aan deelname aan deze studie geen risico's verbonden zijn, besloten u op grond van artikel 4 lid 1 van het Besluit verplichte verzekering bij medisch-wetenschappelijk onderzoek met mensen ontheffing te verlenen van de verzekeringsplicht.

In de beoordeling betrokken documenten:
- protocol versie 2 d.d. februari 2009;
- overzicht documenten;
- uitnodigingsbrief 1e screening versie 2 d.d. 2 februari 2009;
- herinneringsbrief 1e screening versie 2 d.d. 2 februari 2008;
- niet-bereikt brief 1e screening versie 1 d.d. 18 december 2008;
- afsprakenbrief 1e screening versie 2 d.d. 2 februari 2009;
- vergeetbrief afspraak 1e screening versie 1 d.d. 18 december 2008;
- uitslagbrief 1e screening versie 1 d.d. 18 december 2008;
- uitnodigingsbrief 2e screening versie 1 d.d. 18 december 2008;
- afsprakenbrief 2e screening – gelijk aan afsprakenbrief 1e screening;
- uitslagbrief 2e screening versie 1 d.d. 18 december 2008;
- uitnodigingsbrief interventieonderzoek versie 1 d.d. 18 december 2008;
- deelnamebrief interventiegroep versie 1 d.d. 18 december 2008;
- deelnamebrief controlegroep versie 1 d.d. 18 december 2008;
- informed consent screening versie 2 d.d. 2 februari 2009;
- informed consent vervolgonderzoek versie 2 d.d. 2 februari 2009;
- ABR-formulier nr. 25383, versie 03, d.d. 2 maart 2009;
- AMC-appendix;
- wervingstekst versie 2 d.d. 2 februari 2009;

Meibergdreef 9 Postbus 22660 1100 DD Amsterdam Telefoon (020) 566 9111 Fax (020) 566 4440

13270(1)
