# Supplementary material for: Intensive Lifestyle Intervention in General Practice to Prevent Type 2 Diabetes among 18 to 60-Year-Old South Asians: 1-Year Effects on the Weight Status and Metabolic Profile of Participants in a Randomized Controlled Trial
Source: PLoS One. 2013 Jul 22;8(7):e68605. doi: 10.1371/journal.pone.0068605 (PMC3718785; doi:10.1371/journal.pone.0068605)
Supplement: Protocol S5 — Copy trial protocol approval by ethics committee before changes to the protocol part 2. (PDF) [file pone.0068605.s006.pdf]

amc

Academisch Medisch Centrum

Universiteit van Amsterdam

- wervingsbrochure versie 2 d.d. 2 februari 2009;
- lokale uitvoerbaarheidverklaring d.d. 27 februari 2009 van de raad van bestuur van de Stichting Huisartsen Laboratorium Etten-Leur.

Wij verzoeken u onze commissie op de hoogte te stellen van de daadwerkelijke start van het onderzoek, van de (al dan niet voortijdige) beëindiging daarvan, en van tijdens de studie optredende onverwachte complicaties. Voorts dienen eventuele protocolwijzigingen ter beoordeling aan onze commissie te worden voorgelegd.

Wij wijzen u erop dat op grond van artikel 23 van de Wet medisch-wetenschappelijk onderzoek met mensen degene wiens belang rechtstreeks bij een besluit van de MEC is betrokken, daartegen binnen zes weken na de dag waarop het besluit bekend is gemaakt, een administratief beroepschrift kan indienen bij de Centrale Commissie Mensgebonden Onderzoek. Een dergelijk administratief beroepschrift dient geadresseerd te worden aan: CCMO, Postbus 16302, 2500 BH Den Haag.

Tenslotte brengen wij onder uw aandacht dat dit besluit zijn geldigheid verliest als de studie niet binnen twee jaar na dagtekening van deze brief is gestart.

Ten tijde van de beoordeling van dit project was de commissie als volgt samengesteld:

prof. dr. R.T. Krediet	:	voorzitter, hoogleraar nefrologie
prof.dr. P.M.M. Bossuyt	:	plv. lid, hoogleraar klinische epidemiologie
dr. H.H.F. Derkx	:	kindergastro-enteroloog
mw. mr. M.L.M. van der Hulst	:	secretaris, jurist
dr. R.E. Jonkers	:	longarts/klinisch farmacoloog
mw.dr. J.C. Korevaar	:	plv. lid, klinisch epidemioloog
dr. G.A. van Montfrans	:	internist
mw.dr. W.M.C. Mulder	:	plv. lid, klinisch farmacoloog
dr. J.B. Reitsma	:	plv. lid, klinisch epidemioloog
dr. J.F.M. Slors	:	chirurg
prof. dr. J.G.P. Tijssen	:	hoogleraar klinische epidemiologie van hart- en vaatziekten
mw.dr. M.D. Trip	:	internist
drs. A. Vyth	:	ziekenhuisapotheker
mw. C. Webeling	:	beoordeelt onderzoek specifiek vanuit de invalshoek van de patiënt
prof.dr. D.L. Willems	:	hoogleraar medische ethiek.

Met vriendelijke groet,
namens de Medisch Ethische Commissie,

Mw.mr. M.L.M. van der Hulst,
secretaris

c.c. CCMO – **registratienummer NL25383.018.08**
c.c. AMC Medical Research BV

114184(1)
