# Supplementary material for: Intensive Lifestyle Intervention in General Practice to Prevent Type 2 Diabetes among 18 to 60-Year-Old South Asians: 1-Year Effects on the Weight Status and Metabolic Profile of Participants in a Randomized Controlled Trial
Source: PLoS One. 2013 Jul 22;8(7):e68605. doi: 10.1371/journal.pone.0068605 (PMC3718785; doi:10.1371/journal.pone.0068605)
Supplement: Protocol S6 — Copy trial protocol approval by ethics committee after changes to the protocol part 1. (PDF) [file pone.0068605.s007.pdf]

Academisch Medisch Centrum

Universiteit van Amsterdam

mw.mr. Z.K. Ottovay	:	plv. lid, jurist
mw.mr. M.C. Ploem	:	jurist
dr. J.B. Reitsma	:	plv. lid, klinisch epidemioloog
prof.dr. J.G.P. Tijssen	:	hoogleraar klinische epidemiologie van hart- en vaatziekten
mw. C. Webeling	:	beoordeelt onderzoek vanuit de invalshoek van de proefpersoon
prof.dr. D.L. Willems	:	hoogleraar medische ethiek
mw.dr. M.C.B. van Zwieten	:	plv. lid, medisch ethicus.

Met vriendelijke groet,
namens de Medisch Ethische Commissie,

Mw. dr. Y.E. Donselaar,
ambtelijk secretaris

c.c. CCMO – **registratienummer NL25383.018.08**

Meibergdreef 9 Postbus 22660 1100 DD Amsterdam Telefoon (020) 566 9111 Fax (020) 566 4440

132700(1)

8 cc Vera, Charlotte

Academisch Medisch Centrum

Universiteit van Amsterdam

Mw.prof.dr. K. Stronks
Sociale geneeskunde
J2-216

Amsterdam, 4 februari 2010
uw kenmerk:
ons kenmerk: MEC 08/371 # 10.17.0155
betreft:

Medisch Ethische Commissie
E2-236
doorkiesnummer: 566 7389/566 5880
fax: 5669015

Positief nader oordeel m.b.t. het project MEC 08/371:
De effectiviteit van een aangepast diabetes preventie programma voor Hindostaanse Surinamers, bestaande uit een screening gevolgd door een leefstijl interventie.

Geachte mevrouw Stronks,

In vervolg op de ontvangst van uw reactie d.d. 18 januari 2010 op onze brief van 23 decem[illegible] jl. delen wij u inzake het amendement op bovengenoemd project, ons ter beoordeling voorgel[illegible] op 7 december 2009, gaarne mee dat onze commissie

- tot oordelen bevoegd krachtens artikel 2, tweede lid, onder a, van de Wet medisch-wetenschappelijk onderzoek met mensen (WMO);
- werkzaam volgens de ICH-GCP richtlijnen;
- op grond van de haar voorgelegde stukken als hierna vermeld;
- gelet op artikel 3 van de WMO;
- vastgesteld hebbend dat aan de beoogde proefpersonen op adequate wijze informatie wordt gegeven over het uit te voeren onderzoek,

heeft besloten tot een positief nader oordeel over deze studie en de uitvoering daarvan in het AMC.

In de beoordeling betrokken stukken:

A1	aanbiedingsbrief d.d. 7 december 2009 en 18 januari 2010;
C1	protocol versie 5 d.d. januari 2010;
E3	uitnodigingsbrief 1e check versie 3, d.d. 7 december 2009;
E3	uitnodigingsbrief interventieonderzoek versie 2, d.d. 7 december 2009;
E3	folder dhiaan versie 3, d.d. 4 november 2009;
E3	poster vrijwilligers versie 1, 4 november 2009;
F4	uitslagbrief 1e check, versie 2, 7 december 2009.

Wij wijzen u erop dat op grond van artikel 23 van de Wet medisch-wetenschappelijk onderzoek met mensen degene wiens belang rechtstreeks bij een besluit van de MEC is betrokken, daartegen binnen zes weken na de dag waarop het besluit bekend is gemaakt, een administratief beroepschrift kan indienen bij de Centrale Commissie Mensgebonden Onderzoek. Een dergelijk administratief beroepschrift dient geadresseerd te worden aan: CCMO, Postbus 16302, 2500 BH Den Haag.

Ten tijde van de beoordeling was de commissie als volgt samengesteld:

prof.dr. R.T. Krediet	:	voorzitter, hoogleraar nefrologie
prof.dr. P.M.M. Bossuyt	:	plv. lid, hoogleraar klinische epidemiologie
mw. J.M.M. Dijkstra	:	plv. lid dat onderzoek beoordeelt vanuit de invalshoek van de proefpersoon
dr. R.E. Jonkers	:	longarts/klinisch farmacoloog
mw.dr. E.M. Kemper	:	ziekenhuisapotheker
mw.dr. J.C. Korevaar	:	plv. lid, klinisch epidemioloog
mw.prof.dr. L. Lie A Huen	:	plv. ziekenhuisapotheker/klinisch farmacoloog
dr. G.A. van Montfrans	:	internist
mw.dr. W.M.C. Mulder	:	plv. lid, klinisch farmacoloog

Meibergdreef 9 Postbus 22660 1100 DD Amsterdam Telefoon (020) 566 9111 Fax (020) 566 4440

1327(01)
