# Supplementary material for: Intensive Lifestyle Intervention in General Practice to Prevent Type 2 Diabetes among 18 to 60-Year-Old South Asians: 1-Year Effects on the Weight Status and Metabolic Profile of Participants in a Randomized Controlled Trial
Source: PLoS One. 2013 Jul 22;8(7):e68605. doi: 10.1371/journal.pone.0068605 (PMC3718785; doi:10.1371/journal.pone.0068605)
Supplement: Protocol S7 — Copy trial protocol approval by ethics committee after changes to the protocol part 2. (PDF) [file pone.0068605.s008.pdf]

Academisch Medisch Centrum

Universiteit van Amsterdam

Aan mevrouw prof.dr. K. Stronks
sociale geneeskunde
J2-216

Medisch Ethische Toetsingscommissie
E2-236
telefoon: 020 56 67389
fax: 020 5669015

Amsterdam, 16 februari 2012
ons kenmerk: 2008_371#B201276
betreft: **Positief nader oordeel**
NL25383.018.08
The effectiveness of a targeted diabetes prevention programme far Hindustani Surinamese, consisting of screening followed by a lifestyle intervention

Geachte mevrouw Stronks,

De METC AMC heeft zich, op grond van artikel 2, lid 2, sub a van de Wet medisch-wetenschappelijk onderzoek met mensen (WMO) beraden over het amendement behorend bij bovengenoemd onderzoeksdossier.
Wij delen u gaarne mee dat onze commissie
- tot oordelen bevoegd krachtens artikel 2, tweede lid, onder a, van de Wet medisch-wetenschappelijk onderzoek met mensen (WMO);
- werkzaam volgens de ICH-GCP richtlijnen;
- op grond van de haar voorgelegde stukken als hierna vermeld;
- gelet op artikel 3 van de WMO;
- gelet op artikel 5 en 6,

heeft besloten tot een positief nader oordeel over deze studie en de uitvoering daarvan in het AMC.

In de beoordeling betrokken documenten:
A1 aanbiedingsbrief d.d. 2 februari 2012
C1 protocol versie 6 d.d. 2 februari 2012 TC
E5 uitslagbrief nameting na 2 jaar versie d.d. 2 februari 2012 TC

Het amendement, aan ons ter beoordeling voorgelegd op 6 februari 2012 en in behandeling genomen is vervolgens aan de orde geweest in de vergadering(en) van het dagelijks bestuur van onze commissie van 14 februari 2012.

Wij wijzen u erop dat op grond van artikel 23 van de Wet medisch-wetenschappelijk onderzoek met mensen degene wiens belang rechtstreeks bij een besluit van de METC is betrokken, daartegen binnen zes weken na de dag waarop het besluit bekend is gemaakt, een administratief beroepschrift kan indienen bij de Centrale Commissie Mensgebonden Onderzoek. Een dergelijk administratief beroepschrift dient geadresseerd te worden aan: CCMO, Postbus 16302, 2500 BH Den Haag.

Wellicht ten overvloede wijst de METC erop dat de verplichtingen die bij het oorspronkelijke positieve besluit zijn vermeld, onverminderd van kracht zijn

Ten tijde van de beoordeling was de commissie als volgt samengesteld:

mw.dr. M.D. Trip	:	voorzitter, internist
dr. D.K. Bosman	:	kinderarts
prof.dr. P.M.M. Bossuyt	:	plv. lid, hoogleraar klinische epidemiologie
dr. M.G.W. Dijkgraaf	:	plv lid, methodoloog
mw. J.M.M. Dijkstra	:	plv. lid dat onderzoek beoordeelt vanuit de invalshoek van de proefpersoon
prof.dr. R.C.M. Hennekam	;	hoogleraar kindergeneeskunde en klinische genetica

dr. R.E. Jonkers	:	longarts/klinisch farmacoloog
mw.dr. E.M. Kemper	:	ziekenhuisapotheker, plv. klinisch farmacoloog
dr. M.J.W. Koelemay	:	vaatchirurg
prof.mr.dr. J. Legemaate	:	plv lid, hoogleraar gezondheidsrecht
prof. dr. R.A.A. Mathôt	:	plv. Ziekenhuisapotheker/ klinisch farmacoloog
dr. G.A. van Montfrans	:	internist
mw.dr. W.M.C. Mulder	:	plv. lid, klinisch farmacoloog
mw.mr.dr. M.C. Ploem	:	gezondheidsjurist
prof.dr. J.G.P. Tijssen	:	hoogleraar klinische epidemiologie van hart- en vaatziekten
mw. C. Webeling	:	beoordeelt onderzoek vanuit de invalshoek van de proefpersoon
mw dr A.M. Westermann	:	internist-oncoloog
prof.dr. M. Vermeulen	:	hoogleraar neurologie
prof.dr. D.L. Willems	:	hoogleraar medische ethiek
mw.dr. M.C.B. van Zwieten	:	plv. lid, medisch ethicus
prof.dr. A.H. Zwinderman:		plv. lid hoogleraar biostatistiek

Voor de exacte samenstelling van de commissie tijdens de vergadering waarin het besluit is genomen, kunt u contact opnemen met het secretariaat van de commissie.

Met vriendelijke groet,
namens de Medisch Ethische Toetsingscommissie,

Mw dr C.L. van der Wilt,
ambtelijk secretaris

c.c. CCMO (pdf per e-mail)
c.c. pdf per e-mail E.M.A. Vlaar
c.c. CRU (pdf per e-mail)
