# Supplementary material for: Intensive Lifestyle Intervention in General Practice to Prevent Type 2 Diabetes among 18 to 60-Year-Old South Asians: 1-Year Effects on the Weight Status and Metabolic Profile of Participants in a Randomized Controlled Trial
Source: PLoS One. 2013 Jul 22;8(7):e68605. doi: 10.1371/journal.pone.0068605 (PMC3718785; doi:10.1371/journal.pone.0068605)
Supplement: Table S1 — Difference in baseline characteristics between participants with and without follow-up data available after 1 year. (DOC) [file pone.0068605.s009.doc]

**Supplemental Table S1**. Difference in baseline characteristics between participants with and without follow-up data available after 1 year

| **Characteristic** | **Total group**  n=536 | **Follow-up** n=335 | **No follow-up**  n=201 | **Between group difference (95%CI)^a^** | **P value** **^a^** |
| --- | --- | --- | --- | --- | --- |
| **Age in years** | 43.5 (10.4) | 44.9 (10.1) | 41.4 (10.6) | 3.5 (1.7, 5.3) | <0.01 |
| **Men in %** | 271 (50.6) | 170 (50.7) | 101 (50.2) | 0.5 (-8.3, 9.3) | 0.93 |
| **Educational level in %** **^b^** |  |  |  |  |  |
| Low | 61 (11.4) | 39 (11.6) | 22 (11.4) | 0.2 (-6.1, 5.4) | 0.89 |
| Middle | 364 (67.9) | 226 (67.5) | 138 (70.6) | 3.1 (-1.9, 14.4) | 0.13 |
| High | 95 (17.7) | 69 (20.7) | 26 (13.4) | 7.3 (-0.2, 13.6) | 0.06 |
| **Weight status** |  |  |  |  |  |
| Body weight in kg | 75.2 (13.7) | 75.1 (13.5) | 75.3 (14.2) | 0.2 (-2.2, 2.7) | 0.86 |
| BM I in kg/m^2^ | 27.7 (4.1) | 27.7 (3.9) | 27.7 (4.4) | 0 (-0.7, 0.8) | 0.86 |
| Waist circumference in cm | 93 (11) | 93 (10) | 92 (12) | 1 (-1, 3) | 0.42 |
| Hip circumference in cm | 98 (9) | 98 (9) | 99 (12) | 1 (-2, 2) | 0.86 |
| Fat mass in % | 36.1 (9.4) | 36.2 (9.3) | 35.9 (9.5) | 0.3 (-1.5, 1.9) | 0.80 |
| **Glucose metabolism** |  |  |  |  |  |
| HbA1c in mmol/mol | 38 (4) | 39 (4) | 38 (5) | 1 (0.1, 1.7) | 0.02 |
| HbA1c in % | 5.6 (0.4) | 5.7 (0.4) | 5.6 (0.5) | 0.1 (0, 0.2) | 0.02 |
| Fasting plasma glucose in mmol/l | 5.3 (0.5) | 5.3 (0.5) | 5.3 (0.5) | 0 (-0.1, 0.2) | 0.14 |
| 2-h post-load glucose in mmol/l | 6.0 (1.7) | 6.1 (1.7) | 5.8 (1.6) | 0.3 (-0.02, 0.6) | 0.07 |
| Fasting plasma insulin in mmol/l | 14 (8) | 14 (9) | 15 (7) | 1 (-1, 2) | 0.55 |
| **Blood pressure** |  |  |  |  |  |
| Systolic pressure in mm Hg | 129 (17) | 130 (18) | 128 (14) | 2 (-1, 5) | 0.26 |
| Diastolic pressure in mm Hg | 83 (10) | 83 (11) | 83 (10) | 0 (-2, 2) | 0.90 |
| **Lipid profile** |  |  |  |  |  |
| Total cholesterol in mmol/l | 5.04 (0.96) | 5.01 (0.96) | 5.08 (0.96) | 0.07 (-0.10, 0.23) | 0.45 |
| HDL cholesterol in mmol/l | 1.25 (0.30) | 1.27 (0.31) | 1.22 (0.29) | 0.05 (0.00, 0.11) | 0.05 |
| LDL cholesterol in mmol/l | 3.24 (0.89) | 3.19 (0.89) | 3.32 (0.89) | 0.13 (-0.02, 0.29) | 0.10 |
| Triglycerides in mmol/l | 1.33 (0.94) | 1.34 (0.98) | 1.32 (0.88) | 0.02 (-0.15, 0.18) | 0.87 |

BMI = Body mass index, HbA1c = haemoglobin A1c, HDL = high-density lipoprotein, LDL = low-density lipoprotein

Data in parentheses are standard deviations following means or percentages following n

^a^ P values and 95% confidence intervals for differences between control group and intervention group were determined with independent sample *t*-tests for continuous measures and chi-square tests for categorical measures

^b^ Educational level was determined as the highest education achieved and was grouped as: low = secondary primary education or less; middle = low vocational training, lowers secondary education, intermediate vocational training, and higher secondary education; and high = higher vocational training or university
