# Supplementary material for: Intensive Lifestyle Intervention in General Practice to Prevent Type 2 Diabetes among 18 to 60-Year-Old South Asians: 1-Year Effects on the Weight Status and Metabolic Profile of Participants in a Randomized Controlled Trial
Source: PLoS One. 2013 Jul 22;8(7):e68605. doi: 10.1371/journal.pone.0068605 (PMC3718785; doi:10.1371/journal.pone.0068605)
Supplement: Table S2 — Comparison of change in metabolic parameters between those with high and low weight loss, defined by the highest versus lowest quartiles of weight change. (DOC) [file pone.0068605.s010.doc]

**Supplemental Table S2.** Comparison of change in metabolic parameters between those with high and low weight loss, defined by the highest versus lowest quartiles of weight change

|  | **Highest quartile of weight change**  n=84 ^a^ | **Lowest quartile of weight change**  n=82 ^a^ | **Between group differences** | |
| --- | --- | --- | --- | --- |
|  | Change (SD) | Change (SD) | 95% CI | P value ^b^ |
| **Anthropometrics** |  |  |  |  |
| Waist Circumference in cm | -2 (4) | +4 (5) | 6 (5, 8) | <0.01 |
| Hip Circumference in cm | -2 (4) | +1 (4) | 3 (2, 5) | <0.01 |
| Fat mass in % | -2.6 (2.6) | +0.4 (2.8) | 3.0 (2.1, 3.8) | <0.01 |
| **Glucose metabolism** |  |  |  |  |
| HbA1c in mmol/mol | 0 (3) | +1 (3) | 1 (1,2) | <0.01 |
| HbA1c in % | 0 (0.3) | +0.1 (0.3) | 0.1 (0.1, 0.2) | <0.01 |
| Fasting plasma glucose in mmol/l | -0.6 (0.7) | -0.5 (0.8) | 0.1 (-0.1, 0.4) | 0.27 |
| 2-h post-load glucose in mmol/l | -0.3 (1.6) | +0.7 (1.8) | 1.0 (0.4, 1.5) | <0.01 |
| Fasting plasma insulin in mmol/l | -4 (8) | 0 (7) | 4 (1, 5) | <0.01 |
| **Blood pressure** |  |  |  |  |
| Systolic pressure in mm Hg | -1 (10) | +1 (13) | 2 (-1, 6) | 0.15 |
| Diastolic pressure in mm Hg | -5 (7) | -2 (9) | 3 (1, 6) | 0.01 |
| **Lipids** |  |  |  |  |
| Total cholesterol in mmol/l | -0.03 (0.89) | +0.19 (0.86) | 0.22 (-0.05, 0.49) | 0.12 |
| HDL cholesterol in mmol/l | +0.11 (0.20) | +0.05 (0.17) | 0.06 (0.01, 0.12) | 0.03 |
| LDL cholesterol in mmol/l | -0.09 (0.80) | +0.11 (0.75) | 0.20 (-0.04, 0.44) | 0.11 |
| Triglycerides in mmol/l | -0.13 (0.38) | 0.00 (0.55) | 0.13 (-0.02, 0.28) | 0.09 |

BMI = Body mass index, HbA1c = haemoglobin A1c, HDL = high-density lipoprotein, LDL = low-density lipoprotein, SD = standard deviation

^a^ Mean BMI change in highest quartile of weight change: -1.5 kg/m^2^; Mean BMI loss in lowest quartile: +1.36 kg/m^2^

^b^ P values and 95% confidence intervals for differences between control group and intervention group were determined with independent sample *t*-tests
